# Tethered Domains and Flexible Regions in tRNase Z^L^, the Long Form of tRNase Z

**DOI:** 10.1371/journal.pone.0066942

**Published:** 2013-07-17

**Authors:** Christopher Wilson, Daryl Ramai, Dmitri Serjanov, Neema Lama, Louis Levinger, Emmanuel J. Chang

**Affiliations:** 1 Department of Biology, York College of The City University of New York, Jamaica, New York, United States of America; 2 Department of Chemistry, York College of The City University of New York, Jamaica, New York, United States of America; NCI-Frederick, United States of America

## Abstract

tRNase Z, a member of the metallo-β-lactamase family, endonucleolytically removes the pre-tRNA 3′ trailer in a step central to tRNA maturation. The short form (tRNase Z^S^) is the only one found in bacteria and archaebacteria and is also present in some eukaryotes. The homologous long form (tRNase Z^L^), exclusively found in eukaryotes, consists of related amino- and carboxy-domains, suggesting that tRNase Z^L^ arose from a tandem duplication of tRNase Z^S^ followed by interdependent divergence of the domains. X-ray crystallographic structures of tRNase Z^S^ reveal a flexible arm (FA) extruded from the body of tRNase Z remote from the active site that binds tRNA far from the scissile bond. No tRNase Z^L^ structures have been solved; alternative biophysical studies are therefore needed to illuminate its functional characteristics. Structural analyses of tRNase Z^L^ performed by limited proteolysis, two dimensional gel electrophoresis and mass spectrometry establish stability of the amino and carboxy domains and flexibility of the FA and inter-domain tether, with implications for tRNase Z^L^ function.

## Introduction

tRNAs are transcribed as precursors and processed by removal of a 5′ leader and a 3′ trailer (reviewed in [1]), among other reactions including modification, splicing and CCA addition. In a reaction central to tRNA maturation, tRNase Z endonucleolytically removes the 3′ trailer preparing OH^−^ on the discriminator (the unpaired nucleotide at the 3′ side of the acceptor stem) for CCA addition and aminoacylation.

Enzymes involved in general metabolism of tRNAs recognize shared features of tRNAs that are absent from most other RNAs, unlike the specificity of the aminoacyl tRNA synthetases. tRNA end-processing enzymes such as RNase P, tRNase Z and CCA-adding enzyme can utilize as substrate a half-tRNA minihelix consisting of the conserved coaxially stacked acceptor stem and T arm [2]–[4]. tRNase Z recognizes this feature using a distinctive flexible arm (FA) [5]–[12].

### Two forms of tRNase Z

tRNase Z is a member of the β-lactamase family of metal-dependent hydrolases, characterized by an αβ/βα sandwich fold with the two internal β-sheets flanked by α-helices and one or more metal binding sites [13], [14]. tRNase Z can be separately encoded in short (tRNase Z^S^) and long (tRNase Z^L^) forms, and is present, in one form or another, in all eukaryotes and archaebacteria and about half of bacteria [15]. tRNase Z^S^ is the only form in bacteria and archaebacteria. tRNase Z^L^ occurs exclusively in eukaryotes, and is the sole form in *S. cerevisiae*, *C. elegans* and *D. melanogaster*. *D. melanogaster* tRNase Z functions *in vivo* in both nuclear and mitochondrial pre-tRNA maturation [16].

Some eukaryotes including *H. sapiens* encode both tRNase Z^L^ and tRNase Z^S^. tRNase Z^L^ is the better candidate for an essential function in human pre-tRNA maturation due to its >1,000-fold higher reaction efficiency [17] and dual (nuclear and mitochondrial) localization [18]–[20]. The function of human tRNase Z^S^, which localizes to cytoplasm, is unknown.

### The flexible arm of tRNase Z

All tRNase Zs have a flexible arm (FA; see Supplementary Figures SF1, SF5) which recognizes the elbow that caps the coaxially stacked acceptor stem/T arm common to tRNAs^5–11^; no accessory proteins are required for pre-tRNA binding or cleavage [21]. Deleting the FA hand causes an almost 100-fold increase in *K*_m_ for *D. melanogaster* tRNase Z with little effect on *k*_cat_ [11], quantifying its recognition/binding function.

### Domain structure

**Figure 1:**
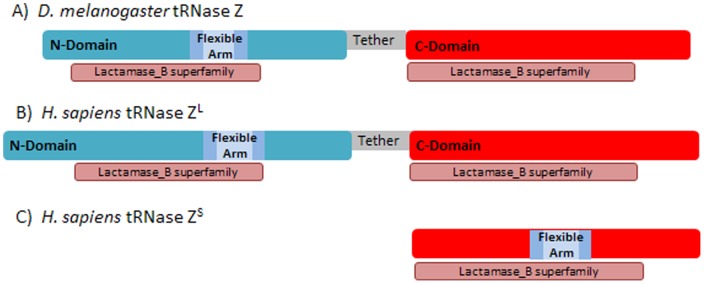
Evolution of tRNase Z^L^ from tRNase Z^S^ by tandem duplication/adaptation. A) *D. melanogaster* tRNase Z; B) *H. sapiens* tRNase Z^L^; C) *H. sapiens* tRNase Z^S^ adapted from Domain Search entries in NCBI. Pink regions below amino and carboxy domains, with annotated homology to β-Lactamase superfamily, are consistent with the evolution of tRNase Z^L^ from tRNase Z^S^ by tandem duplication/adaptation. *D. melanogaster* tRNase Z is accession # NP_724916, taken from discovery of a juvenile hormone inducible cDNA (JHI-1 [43]) that was later found to be *D. melanogaster* tRNase Z [15], . The entry used for *H. sapiens* tRNase Z^L^ is accession # NP_060597, which was reported to be a candidate human prostate cancer susceptibility gene (ElaC2^22^) and later shown to be human tRNase Z^L^ [15], [44]. *H. sapiens* tRNase Z^S^ is accession # NP_061166 (ElaC1 [22]). Pink rectangles below sequence tracks indicate the position and boundaries of the blocks that display the greatest sequence similarity to a proto-metallo-β-lactamase unit. The carboxy domain displays greater homology to the archetypal β-Lactamase superfamily than the amino domain. Additional annotations: the flexible arm (FA) is found only in the amino domain (N-Domain) of tRNase Z^L^ and N-Domain and C-Domain are linked by a flexible tether.

tRNase Z^L^ consists of two domains, both similar to a β-lactamase metalloenzyme unit^22^, tethered by a 70–85 residue spacer [23], suggesting that tRNase Z^L^ evolved from a tandem duplication of tRNase Z^S^ (Figure 1). The C-domain retained the functional metallo-β-lactamase unit and catalytic activity but lost the flexible arm. The N-domain retained the FA and thus functions in initial substrate recognition and binding; architectural features of a metallo-β-lactamase are still present, but the metal-binding residues and active site were lost. While tRNase Z^S^ is a functional homodimer, tRNase Z^L^ presumably acts as a monomer.

No experimental tRNase Z^L^ structures have been reported, and important functional characteristics of tRNase Z^L^, especially those of the more diverged amino domain and the tether, cannot be deduced from available tRNase Z^S^ structures. Alternative biophysical means for investigating tRNase Z^L^ are therefore needed.

### Investigation of tRNase Z^L^ by proteolysis, electrophoresis and mass spectrometry

Endoproteinases cleave adjacent to a specific amino acid residue at regions on the surface of a protein that are solvent-exposed and flexible enough to fit the active site geometry of the protease [24]–[26], typically 8 to 10 residue stretches of the native protein. Such flexible regions generally occur in loops or secondary structure elements capable of unfolding [24]–[28]. Initial cleavages, before tertiary structure of the protein deteriorates, occur in the flexible regions, and stable domains are more resistant to proteolysis. Flexible regions determined by limited proteolysis and mass spectrometry correlate with polypeptide disorder as determined by NMR [29], X-ray crystallographic B-factors [27], [30], fluorescence spectroscopy [31], circular dichroism spectroscopy [31] and computational molecular dynamics [27].

Limited proteolysis fragments can be analyzed using a combination of gel electrophoresis, amino acid analysis and N-terminal sequencing [27], [29], [31]. The advent of high-resolution protein mass spectrometry enabled precise protease site mapping [30]–[32]. Linear mode matrix-assisted laser desorption/ionization – Time-of-Flight (MALDI- TOF) analysis [33], [34] is well-suited for the mass determination of moderate to large polypeptides such as tRNase Z proteolysis products because of its practically unlimited mass range, and is complementary to gel electrophoresis because it produces accurate molecular weights of these proteolysis products with higher resolution and precision, while electrophoresis is better for quantification of relative polypeptide abundance.

To investigate domain structure and flexibility of tRNase Z^L^, we subjected *D. melanogaster* and *H. sapiens* tRNase Z^L^ to limited tryptic proteolysis and analyzed the products by one- and two-dimensional gel electrophoresis and MALDI-TOF mass spectrometry. 2D gels resolve roughly an order of magnitude more spots than 1D gels, relieving issues of heterogeneous bands (1D SDS-PAGE) and spectral crowding (MALDI-TOF).

Protease accessible regions fall within specific regions in tRNase Z^L^. Cleavage in the inter-domain tether unlinks the protein into stable domains, consistent with tandem duplication. The region in and around the flexible arm is accessible. Hydrophilic regions close to both termini are highly flexible. A similar domain structure is observed for *H. sapiens* tRNase Z^L^.

Theoretical tryptic digestion of tRNase Z produces 3,081 polypeptides. 2D electrophoresis resolves about 6 families averaging 5–10 spots of related polpypeptides each, resulting in ∼60 species, representing <2% of the potential. Limited proteolysis thus indeed generates stable domains from cleavage in flexible regions.

The analysis was extended by probing functionally deficient tRNase Z sequence variants and by using proteases with complementary specificity. Flexibility of the inter-domain tether, first observed here, may contribute to greater catalytic efficiency of tRNase Z^L^ over tRNase Z^S^.

## Materials and Methods

### Protein preparation

Fruit fly tRNase Z with an N-terminal 6× His tag was baculovirus-expressed and affinity purified using nickel-chelate resin (Qiagen) as previously described [21]. Protein was expressed from an internal methionine (M_24_AAT) that is taken to be the translation start for the nuclear form of fruit fly tRNase Z, two residues before the predicted amino end of the mitochondrial form after import and cleavage [16], and is thus numbered M_1_ throughout. Removing the His-tag using rTEV protease (Invitrogen) leaves a 7 residue leader (GAMDPEF) which was added to the tRNase Z for interpretation of masses in the spectra.

Approximately 500 µg of native tRNase Z was further purified by gel filtration using a Superose 6 10/300 GL column at 4°C on the Akta Purifier 10 platform (GE Life Sciences) using 250 mM KCl, 25 mM Tris-HCl pH 7, 1 mM DTT as column buffer. Ovalbumin, bovine serum albumin and alcohol dehydrogenase were used as size standards. Peak fractions were pooled and concentrated using a 10 kD NMWL microconcentrator (Millipore). Proteins were analyzed using 1D SDS-PAGE with a 10% polyacrylamide separating gel and a 4% stacking gel with Tris-Glycine buffers containing 0.1% SDS. Gels were stained with Sypro orange (Molecular Probes) and scanned with a Typhoon 9210 (GE Life Sciences).

### Processing analysis

**Figure 2:**
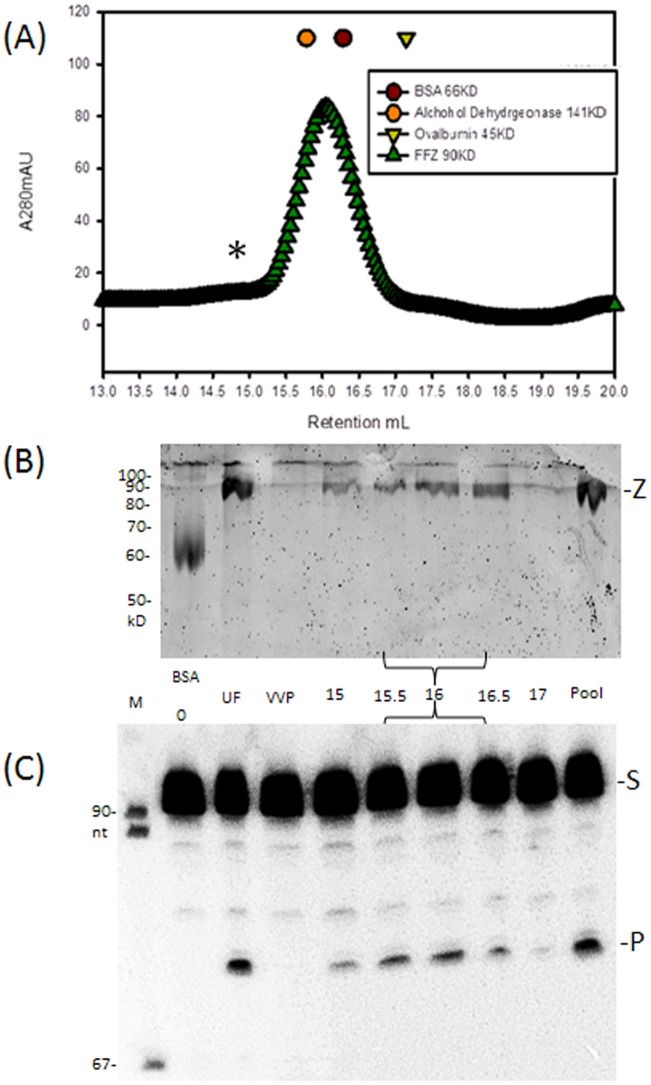
tRNase Z purifies as a monomer. (A) Affinity purified *D. melanogaster* tRNase Z was separated by SEC using a Superose 6 10/300 GL column on the Akta Purifier 10 platform (GE Life Sciences). Symbols above the absorbance profile indicate elution peaks for Ovalbumin (45 kD), BSA (66 kD) and Alcohol Dehydrogenase (141 kD). tRNase Z (green triangles) chromatograms as a monomer true to its ∼84 kD molecular weight. * identifies a shoulder in the chromatogram coinciding with expected position of the tRNase Z^L^ dimer which is apparently low in quantity and enzyme activity. Lines beneath axis indicate peak fractions. B) Protein gel. UF: Unfractionated tRNase Z. Pool – peak fractions were pooled and concentrated. C) Processing assay of three peak fractions compared with UF indicates that gel filtration improves enzyme specific activity. Each fraction was incubated with end-labeled substrate for 15 min. and electrophoresed on a denaturing polyacrylamide gel. S, P at right designate the 90 nt *D. melanogaster* pre-tRNA^Arg^ substrate and 73 nt product, respectively. Lane 0: no processing control.

The sample applied to the column, gel filtration fractions and the concentrated pooled eluate were assayed for tRNase Z activity using a 5′-endlabeled fruit fly pre-tRNA^Arg^ substrate [35]. Gel lanes (Figure 2C) were scanned with a Typhoon 9210 and peak areas were obtained using ImageQuant. Enzyme specific activity was estimated by dividing % product/minute of reaction by enzyme concentration (estimated from the protein gel lanes compared with the BSA standard; Figure 2B).

### Protease digestion, 1D electrophoresis and mass spectrometry

12 µg of pooled concentrated *D. melanogaster* tRNase Z SEC eluate was digested with 0.167 ng/mL trypsin (Promega; a 1,200∶1 tRNase Z: trypsin mass ratio) at 37 C in 60 µL of 50 mM ammonium bicarbonate pH 8. For *H. sapiens* tRNase Z^L^, twice the trypsin concentration was used. Reactions were sampled before addition of trypsin and after 1, 2, 5, 10, 15 and 30 min of reaction. At each time point, 3 µL samples were transferred to protein gel loading buffer for 1D SDS-PAGE and 2 µL samples were transferred to 1 µL of 1% TFA to inactivate trypsin. Samples for mass spectrometry were further mixed in a 1∶1 ratio with a saturated solution of recrystallized α-cyano-4-hydroxycinnamic acid (CHCA, Sigma) in 50% formic acid/33% isopropyl alcohol/17% water (v/v/v; Fisher, all HPLC grade or higher) and prepared for matrix-assisted laser desorption/ionization (MALDI)-time of flight (TOF) mass spectrometry using the ultra-thin method essentially as described [36], [37]. Procedures for protease digestion with endo LysC (Roche) and GluC (Roche or New England Biolabs) were the same as for trypsin except that GluC was used at 83 ng/µL.

Spectra from 10–90 kD were taken using a Waters MaldiMX with suppression set at 9,000. Bovine serum albumin (66,430 DA; Sigma) was used as a mass standard. Full length tRNase Z has a mass of 83,671 Da from the 7 residue leader through M_1_AAT (see above) to T_743_. Some tRNase Z is observed with ∼3 kDA larger mass (86,640 Da) due to incomplete rTEV digestion which leaves an additional 23 residue leader (MSYYHHHHHHDYDIPTTENLYFQ; 2986 Da) from the Fastbac vector. Protein Analysis Worksheet (PAWS; Genomic Solutions, Inc.) was used with observed masses from the spectra and known sequence to collect the interpreted cleavage products with their calculated masses.

### 2D Electrophoresis and mass spectrometry

*D. melanogaster* and *H. sapiens* tRNase Z^L^ samples were treated with trypsin and sampled at various times as in preparation of samples for 1D electrophoresis and MALDI-TOF. 2D electrophoresis was performed with first dimension isoelectric focusing (IEF) in a mixture of pH 3–10 and 5–8 ampholytes using a mini-tube gel system (BioRad). Second dimension SDS-PAGE was performed as for 1D SDS-PAGE. 2D gels were stained with Sypro Orange using Method 2[38] by fixing in 0.0005% SDS, 2% acetic acid, 40% ethyl alcohol, washing twice in 0.0005% SDS, 2% acetic acid, staining twice with Sypro Orange in 0.0005% SDS, 2% acetic acid, rinsing briefly in 0.0005% SDS, 2% acetic acid and scanning with a Typhoon 9210.

Representative polypeptide spots >30 kDa were excised from the 2D gels and subjected to exhaustive in-gel tryptic proteolysis, generally to peptides with molecular weights <4 kDa. These peptides can be analyzed by mass spectrometry using higher resolution single-stage MS to obtain peptide mass maps of each limited proteolysis product, as well as tandem MS (MS/MS) to confirm peptide sequence, both using MALDI-ion trap mass spectrometry. Sensitivity of the MALDI-ion trap mass spectrometry instrument in MS/MS mode [39], [40] allows identification of low abundance polypeptides at the boundaries of the limited proteolysis polypeptides, and the offline, stable nature of MALDI samples allows multiple rounds of interrogation for the manual interpretation of data.

## Results

The long form of tRNase Z (tRNaseZ^L^) from *D. melanogaster* and *H. sapiens* share a domain structure with the short form (tRNase Z^S^). Both amino and carboxy domains display homology to the β-lactamase family of metal-dependent hydrolases (Figure 1, adapted from NCBI Domain Search with additional annotation). It was suggested in 2001, and extensively supported since, that tRNase Z^L^ arose as a tandem duplication of tRNaseZ^S^ with subsequent divergence of the two domains [22]. The distinctive flexible arm of tRNase Z was retained by the amino domain [7] and the metal-binding residues and active site were retained by the carboxy domain.

### Native tRNase Z^L^ purifies as a monomer

While tRNase Z^S^ is a homodimer, domain structure of tRNase Z^L^ suggests that it functions as a monomer. Size exclusion chromatography (SEC) of native soluble tRNase Z^L^ shows a major absorbance peak at the position expected for monomeric tRNase Z^L^ (∼84 kDa) relative to size standards (Figure 2A) which coincides with the protein and processing activity peaks (Figure 2B, C). The precise molecular weight was confirmed by MALDI-TOF mass spectrometry (see below). A minor peak (∼168 kDA; * above chromatogram in Figure 2A) corresponds in position to a tRNase Z^L^ dimer. SEC fractions display greater specific activity than unfractionated tRNase Z, presumably due to removal of trace inhibitors. Aggregated tRNase Z in the small void volume peak is practically inactive.

### Stable domains and flexible regions detected by limited proteolysis

**Figure 3:**
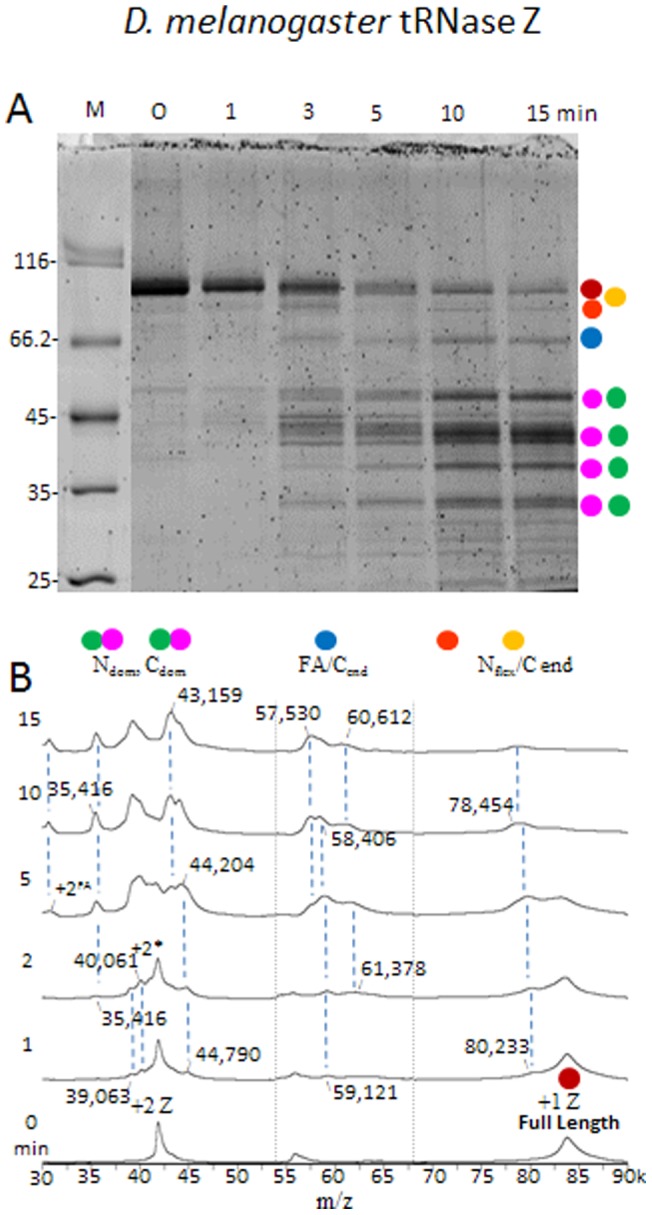
Trypsin digestion of purified tRNase Z followed by mass spectrometry shows the main stable domains and flexible regions. A) Protein gel lanes illustrate extent of digestion of *D. melanogaster* tRNase Z by trypsin. Colored dots (red, orange, blue, magenta and green) indicate full length tRNase Z and the tryptic fragments arising from cleavage at N_flex_, FA, N_dom_-tether and C_end_. B) Spectra obtained by mass spectrometry of the samples illustrated in (A). MALDI-TOF was performed using a Waters MaldiMX. Representative masses are presented on the spectra and the complete table (mass observed, mass calculated, ppm error and theoretical pI) is presented in Supplementary Table ST1.

**Figure 4:**
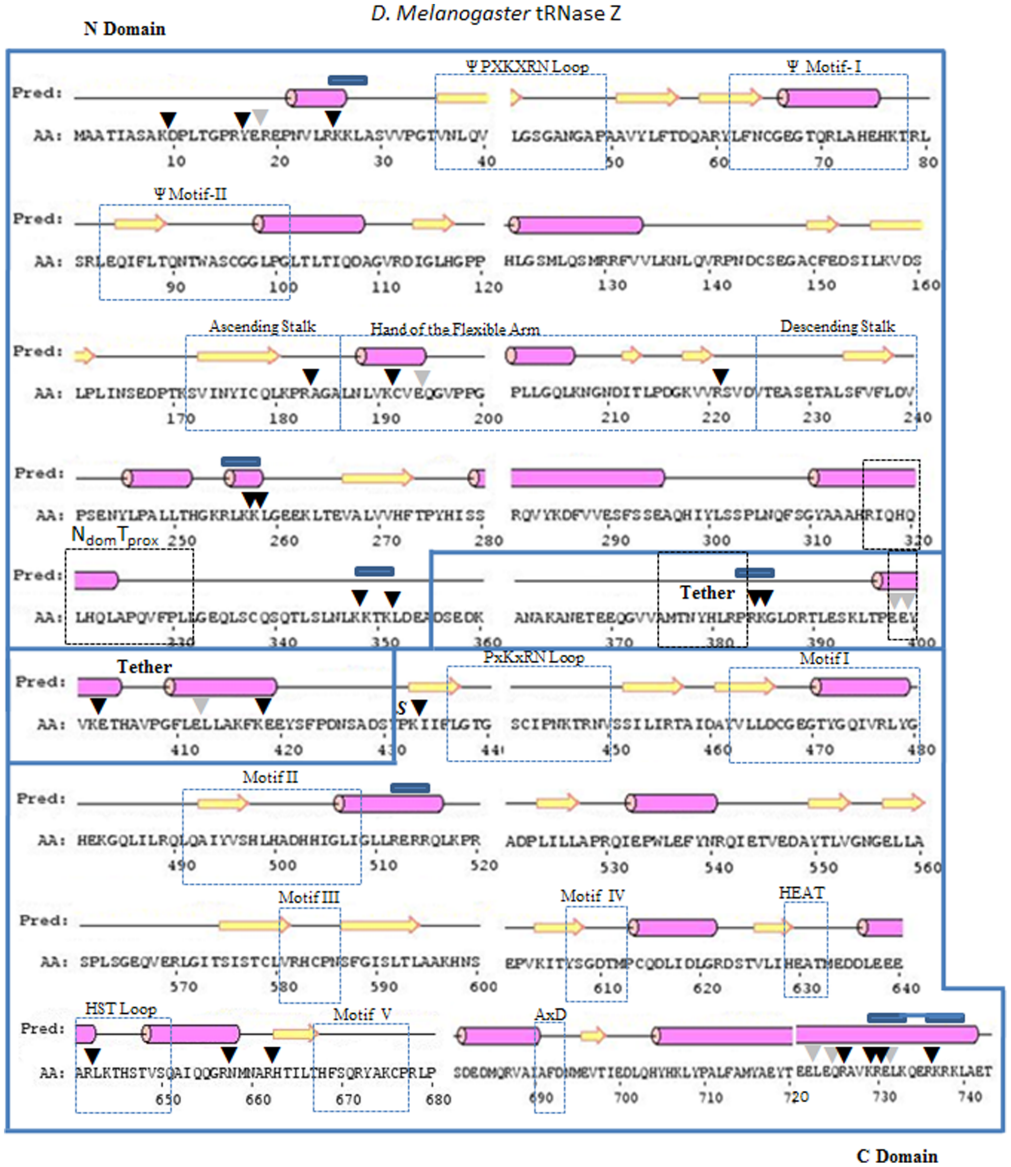
Sequence and predicted secondary structure of *D. melanogaster* tRNase Z (Psipred). Large rectangular enclosures indicate amino domain, tether and carboxy domain. Dashed rectangular enclosures indicate functionally characterized motifs (discussed in detail and further annotated with a multiple sequence alignment in Supplemental Figure SF2 in File S1).

**Figure 5:**
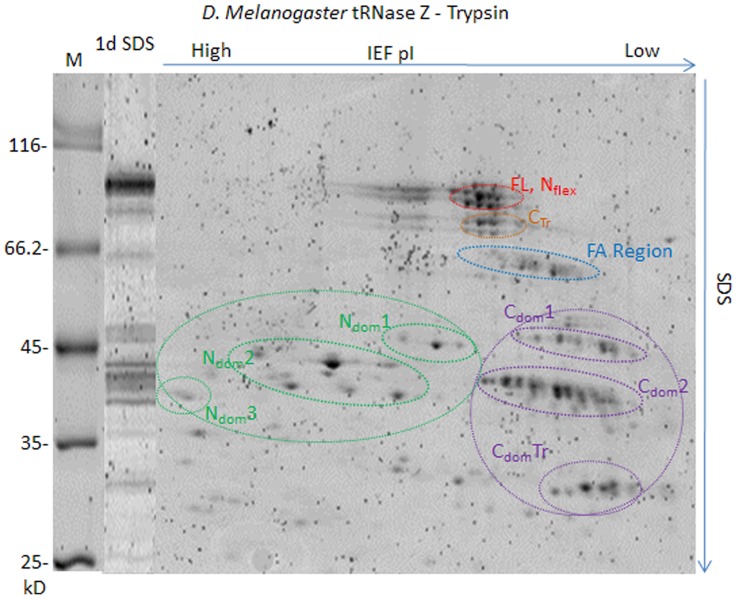
Two dimensional gel electrophoresis of *D. melanogaster* tRNase Z-trypsin polypeptides. A three min. digest like those illustrated in Figure 3 was electrophoresed on a first dimension isoelectric focusing tube gel. The second dimension SDS-PAGE was the same as in Figure 3.

**Figure 6:**
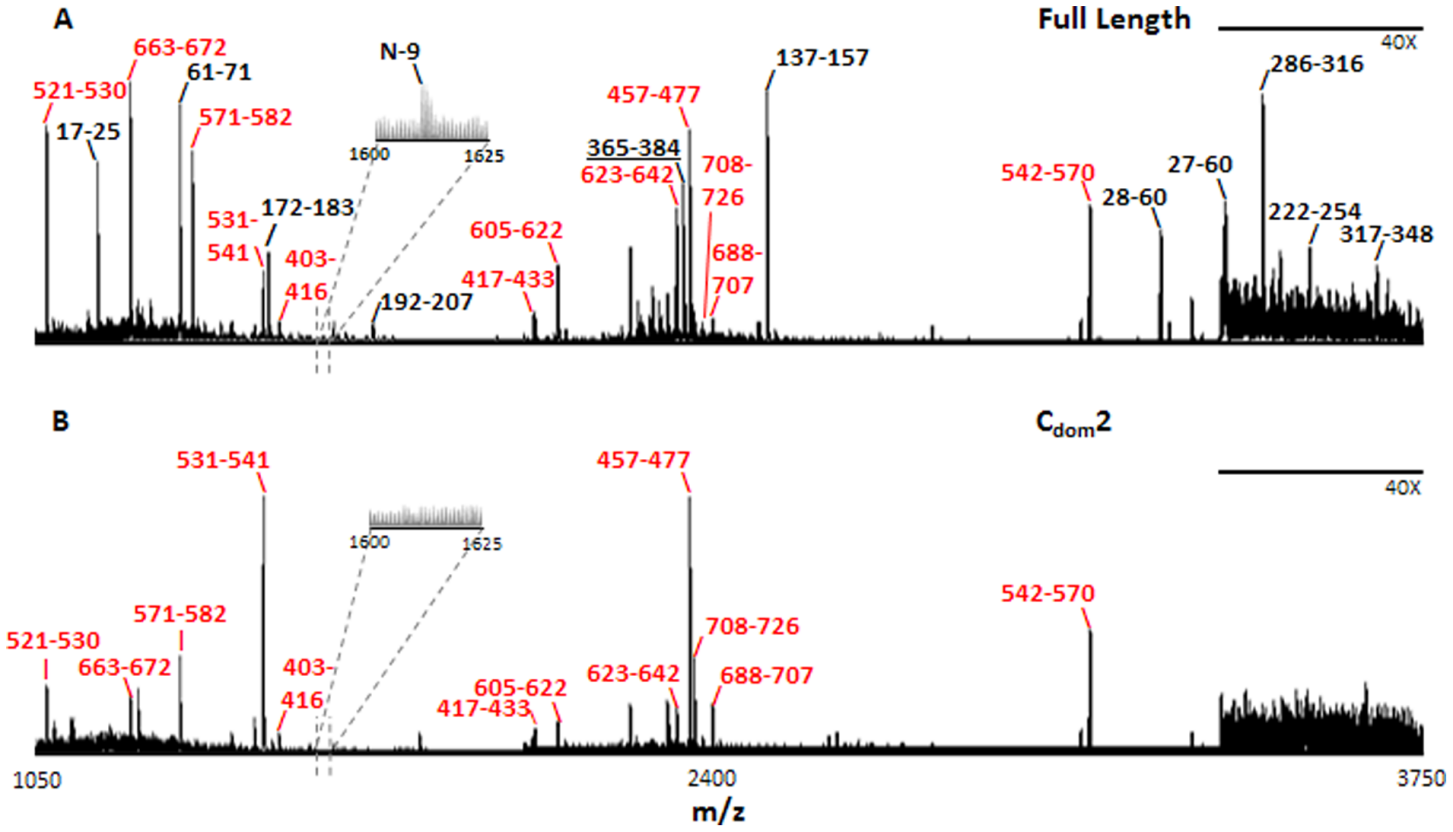
Maldi-ion trap mass spectra of exhaustive in-gel trypsin digest products. Spots were excised from the two dimensional gel (Figure 5). (A) Full length *D. melanogaster* tRNase Z. (B) A representative C_dom_2 partial proteolysis polypeptide. Start and end residue numbers are indicated for all peaks that can be positively assigned to tRNase Z^L^ tryptic peptides. See table 1 for details. Peptides present in both samples are labeled in black; those present in the full length protein but not C_dom_2 are labeled in red.

To investigate stable domains and flexible regions, peak fractions of monomeric *D. melanogaster* tRNase Z (brace below Figure 2B) were pooled, concentrated and subjected to limited proteolysis at a ∼1,200∶1 mass ratio of tRNase Z to trypsin (see Methods) followed by electrophoresis on protein gels and analysis by mass spectrometry (Figure 3). Sequence, domain structure, functional motifs [5]–[11], [13]–[16], [21]–[23], [35], [41], [44] and predicted secondary structure of *D. melanogaster* tRNase Z are presented in Figure 4 with trypsin cleavage sites indicated by vertical arrowheads and more fully described in Supplement (including the multiple sequence alignment in Supplemental Figure SF2 in File S1). MALDI-TOF results are complemented by 2D gel electrophoresis (isoelectric focusing, then SDS-PAGE; Figure 5) followed by mass spectrometric analysis of the gel spots (Figure 6, Table 1).

**Table 1:** *D. melanogaster* tRNase Z Peptide Analysis.

First	Last	MW	Sequence
**N** 1	**9**	**1609.8**	**GAMDPEFMAATIASAK**
**10**	**16**	**754.4**	**DPLTGPR**
**17**	**25**	**1174.6**	**YEREPNVLR**
**27**	**60**	**3383.8**	**KLASVVPGTVNLQVLGSGANGAPAAVYLFTDQAR**
**28**	**60**	**3255.7**	**LASVVPGTVNLQVLGSGANGAPAAVYLFTDQAR**
**61**	**71**	**1357.6**	**YLFNCGEGTQR**
**72**	**77**	**733.4**	**LAHEHK**
**83**	**112**	**3272.7**	**LEQIFLTQNTWASCGGLPGLTLTIQDAGVR**
**113**	**130**	**1945.0**	**DIGLHGPPHLGSMLQSMR**
**131**	**136**	**760.5**	**RFVVLK**
**132**	**136**	**604.3**	**FVVLK**
**137**	**157**	**2479.2**	**NLQVRPNDCSEGACFEDSILK**
**158**	**171**	**1526.8**	**VDSLPLINSEDPTK**
**172**	**183**	**1503.8**	**SVINYICQLKPR**
**192**	**207**	**1704.9**	**CVEQGVPPGPLLGQLK**
**222**	**254**	**3548.8**	**SVDVTEASETALSFVFLDVPSENYLPALLTHGK**
**286**	**316**	**3456.6**	**DFVVESFSSEAQHIYLSSPLNQFSGYAAAHR**
**317**	**348**	**3682.0**	**IQHQLHQLAPQVFPLLGEQLSCQSQTLSLNLK**
***365***	***384***	***2314.1***	***ANETEEQGVVAMTNYHLRPR***
403	416	1523.8	ETHAVPGFLELLAK
417	433	2022.9	FKEEYSFPDNSADSYPK
449	456	900.5	NVSSILIR
457	477	2327.2	TAIDAYVLLDCGEGTYGQIVR
490	512	2603.4	QLQAIYVSHLHADHHIGLGLL
515	520	796.5	RQLKPR
521	530	1077.7	ADPLILLAPR
531	541	1493.7	QIEPWLEFYNR
542	570	3116.6	QIETVEDAYTLVGNGELLASPLSGEQVER
571	582	1332.7	LGITSISTCLVR
583	597	1628.8	HCPNSFGISLTLAAK
605	622	2068.0	ITYSGDTMPCQDLIDLGR
623	642	2302.0	DSTVLIHEATMEDDLEEEAR
643	657	1652.9	LKTHSTVSQAIQQGR
645	657	1411.7	THSTVSQAIQQGR
658	662	604.4	NMNAR
663	672	1238.7	HTILTHFSQR
679	687	1089.5	LPSDEDMQR
688	707	2372.2	VAIAFDNMEVTIEDLQHYHK
708	726	2336.1	LYPALFAMYAEYTEELEQR

Tryptic Peptides from 2D gel spots were analyzed by MALDI-Ion trap MS. All peptides listed are found in full length tRNase Z.

Bold: N_dom_ and tether peptides are found in full length tRNase Z but not in C_dom_2 region.

Italic: Absence of peptide 365–384 from C_dom_2 defines the amino boundary of the C_dom_2 polypeptide.

1 First seven residues are leader following TEV site.

Before the addition of protease (0 min in Figure 3A), the major band in the tRNase Z preparation displays an apparent molecular weight relative to known markers above 85 kDa, consistent with anomalously slow migration observed earlier [11]. Heterogeneous groups of protein bands appear after 3 minutes and are maintained through 15–30 minutes of digestion (identified by colored circles at right in Figure 3A based on size and other evidence presented in detail below). MALDI-TOF analysis of tRNase Z proteolysis products (Figure 3B) yields major *m/z* peaks consistent with these size classes of polypeptides (Figure 3A).

Masses obtained by mass spectrometry are not subject to the discrepancy between apparent molecular weight of tryptic polypeptides relative to marker proteins observed in gel electrophoresis. Because of high mass resolution obtained by MALDI-TOF, peaks can be assigned to specific polypeptides (see Supplemental Table ST1 and Table 2 in File S1). Prior to trypsin addition (Figure 3B, 0 min), the mass spectrum contains two major peaks that correspond to the singly (1+) and doubly (2+) protonated mass-to-charge (*m/z*) values of full length *D. melanogaster* tRNase Z. Because BSA and its multiply protonated species were used for mass calibration, more reliable masses are sometimes achieved using the 2+ ion of tryptic polypeptides whose mass is greater than BSA (see Methods). The calculated mass of tRNase Z is 83,671 Da; the observed mass of the more intense 2+ peak is 83,708 Da. A small peak with *m/z*  = 55,945 Da, consistent with a triply ionized tRNase Z dimer, decreases in intensity with increasing time of reaction; while generally present, its intensity is lower with conservative pooling of SEC fractions.

**Table 2:** *D. melanogaster* tRNase Z – Trypsin Polypeptides.

	Observed Polypeptide	Cleavage Sites		Theoretical MW	Theoretical pI	MALDI-TOF Peaks^1^
		N-End	C-End			
***Full Length***	N – C_end_	N	K_729_ RELKQERKRKLAET	81,904–83,671	6.03–6.32	81,892–83,708
***Flexible Amino End***	N_end_ – C_end_	KD_10_-RY_17_-RE_20_	K_729_ RELKQERKRKLAET	80,066–81,904	6.03–6.32	80,121–81,892
	N_flex_ – C_end_	RK_26_ KL	K_729_ RELKQERKRKLAET	78,161–80,184	5.97–6.25	78,208–80,121
***Carboxy End Truncation***	N_end_ – C_Tr_	N	R_662_	73,918	6.15	73,960
	N_flex_ – C_Tr_	RK_26_ KL	R_662_	70,131–70,387	6.08–6.21	70,280
***Flexible Arm***	FA1 – C_end_	RA_184_	K_729_ RELKQERKRKLAET	61,220–62,987	5.78–5.96	60,487–61,378
	FA2 – C_end_	KC_192_	K_729_ RELKQERKRKLAET	60,453–62,220	5.72–5.90	60,487–61,616
	FA3 – C_end_	RS_222_	K_729_ RELKQERKRKLAET	57,356–59,123	5.71–5.89	57,471–59,121
***Amino End Truncation***	N_int_ – C_end_	KRLKKL_259_	K_729_ RELKQERKRKLAET	53,297–55,590	5.79–6.11	53,445
***Amino Domain DomainTether***	N_end_ – N_dom_1	RY_17_	K_402_	42,589	6.94	ND*
	N_flex_ – N_dom_2	RK_26_ KL	R_384_ KG	39,087–41,237	6.48–7.30	39,062–40,061
	N_flex_ – N_dom_3	RK_26_ KL	K_348_ KTKL	35,044–35,658	7.72–8.42	35,416–35,508
***Carboxy Domain***	C_dom_1 – C_end_	KK_349_TKL	K_729_ RELKQERKRKLAET	43,135–44,902	5.56–5.80	43,121–44,790
	C_dom_2 – C_end_	RK_385_G	K_729_ RELKQERKRKLAET	38,964–40,860	5.64–5.90	39,227–40,061
	C_dom_Tr	RK_385_G	R_642_LK, R_657_, R_662_	28,756–31,106	5.30–5.71	28,754–31,107

Interpretation of limited proteolysis products is based on gel electrophoresis (Figures 3A and 5) and mass spectrometry (Figures 3B and 6).

1 from Figure 3B and Supplemental Table ST1 in File S1.

* An amino end at D10 producing a polypeptide with mass 43,225 DA and pI 6.96 could be assigned from the mass 43,208 Da obtained by MALDI-TOF.

Peaks of lower mass in the 80, 60, 45, 40 and 35 kD regions increase at the expense of full length protein with trypsin incubation time, as in the gel lanes. Representative peak masses are labeled in Figure 3B; matching polypeptides are assigned in Supplemental Table ST1 in File S1 along with detailed interpretation in Supplemental text in File S1. The 52.5 – 67.5 kDa mass region (enclosed in dashed vertical lines in Figure 3B), where polypeptides originating from cleavage in the FA are expected, was expanded 2.5-fold on the y-axis.

tRNase Z polypeptides cannot be unambiguously identified by linear mode MALDI-TOF alone because of peak heterogeneity from theoretical molecular weights too close to be resolved and due to overlap with the +2z region. Interestingly, some peaks arise from a mixture of amino and carboxy domain polypeptides (green and magenta circles, respectively) with almost identical masses (see Supplemental Table ST1 in File S1 for examples).

The major size classes of limited proteolysis polypeptides are separated into clusters of spots (Figure 5) using two-dimensional gel electrophoresis (isoelectric focusing followed by orthogonal SDS-PAGE). Important ambiguities in the 1D SDS-PAGE and MALDI-TOF results (Figure 3, Supplemental Table ST1 in File S1) were resolved. In particular, N_dom_ polypeptides have a higher pI, and C_dom_ polypeptides have a lower pI, than full length tRNase Z (Figure 5, Supplemental Table ST1 in File S1). Additionally, smaller polypeptides including major N_dom_ and C_dom_ species in the 39–42 kDa range which overlap with doubly protonated, higher molecular weight species (Figure 3B) are readily visualized on 2D gels.

Closely spaced spots fall into practically horizontal tracks (enclosed in elongated ellipses in Figure 5), suggesting polypeptide families with incremental charge and mass differences produced by cleavages at clusters of basic residues at both ends. These families are shown below to be cleaved at or N-terminal to a basic patch close to the amino end (N_flex­_, red), from amino end or N_flex_ to a truncation site in the carboxy domain (C_Tr_, orange), from the flexible arm into the carboxy domain (FA region, blue), and specific to the amino (N_dom_, green) and carboxy (C_dom_, magenta) domains with varying amounts of tether attached.

The *D. melanogaster* C_dom_2 family (Figure 5) has more than 10 spots in a slightly oblique track (pI decreases with mass). This large family can be explained (Supplemental Table ST2 in File S1) by trypsin cleavage in tether (R_384_ KG; potential cleavage sites underlined) and in a basic patch close to the carboxy end (C_end_; K_729_ RELKQERKRKLAET_743_). The predicted spots with highest mass and pI (Supplemental Table ST2 in File S1) are K_385_–T_743_ (40,860 Da; 5.90) and K_385_–K_739_ (40,445 Da; 5.99); the lowest is G_386_–K_729_ (38,964 Da; 5.55).

The interpretation is supported by MALDI-trap peptide analysis of polypeptide spots extracted from full length *D. melanogaster* tRNase Z (with coverage illustrated in Supplemental Figure SF3 in File S1) and C_dom_2 spots (Figure 6, Table 1). The amino boundary of C_dom_2 polypeptides must not come before R_382_P_383_RK_385_ because the peptide A_365_NETEEQGVVAMTNYHLRPR_384_ observed in full length tRNase Z (underlined in Figure 6A, italic in Table 1) was not found in the C_dom_2 spots. Polypeptide analysis does not rule out amino ends at T_390_ and L_395_, however; faint parallel tracks observed below the main track of the C_dom_2 family (Figure 5) are consistent with the 500–1,000 Da reduced mass predicted for polypeptides that begin in tether several residues closer to the C-end.

Analytical methods described in detail for the C_dom_2 region were applied to other families consisting of intense resolved spots from the 2D gel (enclosed in colored ellipses in Figure 5) with results presented in Table 2 (additional detail provided in Supplement). Polypeptides are identified by the following procedures: (1) Preliminary assignments with high mass accuracy are made using MALDI-TOF spectra (Figure 3B, Supplemental Table ST1 in File S1). (2) Relative molecular weight and pI information from the 2D gel (Figure 5) is combined with predicted molecular weights and pIs (Supplemental Table ST1 in File S1) and by modeling as for C_dom_2 polypeptides (Supplemental Table ST2 in File S1) based on sequence. (3) Boundaries are established for polypeptides from the 2D gels by peptide analysis (Figure 6, Table 1, Supplemental Table ST3 in File S1and Supplemental Appendix S1).

The largest trypsin cleavage products (N–C_end_) arise from cleavage within a hydrophilic flexible region close to the carboxy end (C_end_) as described above for C_dom_2. The observed reduction in pI with mass (predicted 6.32–6.03; see Table 2) is due to C_end_ trimming of 3 more basic than acidic residues.

Shortening from the amino end of tRNase Z (KD_10_–RY_17_–RE_20_) culminates in a hydrophilic basic patch (R_25_ KKL) designated N_flex_ which, accompanied by C_end_ trimming, produces the N_flex_-C_end_ family. The predicted mass range (78,161–81,904 Da) is consistent with observation (Figures 3, 5 and Table 2).

Truncation within the carboxy domain (CTr) by cleavage at an internal site (R662) produces two polypeptides, from Nend and from Nflex, with masses around 74 and 70 kDa, respectively. While clearly resolved on gels (Figures 3A and 5), these species display low intensity in the spectra (Figure 3B). The characteristic ∼4 kDa spacing between Nend/Nflex, repeated below in two tracks of CTr polypeptides, suggests that a strong stop at Nflex (R_25_KKL) dominates the gradual trimming from the amino end (KD_10_–RY_17_–RE_20_–RK_26_).

The tRNase Z FA consists of a globular hand (two α helices connected by a GP-rich loop, two antiparallel β strands and a turn of 3/10 helix; α_4_α_5_GPLβ_10_β_11_η following the numbering of *B. subtilis* tRNase Z^S^ secondary structure elements [5]) extruded from the body of the enzyme by a structured stalk (Supplemental Figures SF1, SF5 in File S1; cf [5]). Three main cleavage sites were found in the FA region: RA_184_ in the ascending stalk and KC_192_ and RS_222_ in the FA hand (in α4 and just after β11, respectively; see Figure 4 and Supplement for annotation and additional structural detail). Heterogeneity of mass (from ∼57.5 to ∼61.5 kDa) and pI arises due to Cend trimming as described above. Taking into consideration the anomalously slow migration relative to known markers, the tracks in the FA family enclosed in ellipses (Figure 5) are consistent with the three main classes of peak masses observed in MALDI-TOF spectra (Figure 3B, Supplemental Table ST1 in File S1).

A weaker track of bands that appears at lower apparent molecular weight and higher relative pI just below the FA ellipse is consistent with the N_int_-C_end_ species (amino end K258KL) found in the MALDI-TOF spectra (peaks detected at 53,429–53,445–54,551 Da; theoretical pI around 5.8; Supplemental Table ST1 in File S1, Table 2). The lower intensity of these polypeptide species in both the gel and the MALDI-TOF spectra suggests these to be lower protease accessible/flexibility regions.

The amino domain (N_dom_) region displays N_dom_1, N_dom_2, N_dom_3 sub-families which increase in pI with decreasing mass, depending mainly on the length of retained tether. N_dom_3, the smallest stable species, begins at N_flex_ (R_25_ KKL) and ends at the N_dom_-tether boundary (K_348_ KTK), supported by polypeptide species K_27_–K_351_, K_26_–K_349_ and L_28_–K_351_ from MALDI-TOF in the 35,416–35,508 Da mass and 7.72–8.16 pI range (Figure 3B, Supplemental Table ST1 in File S1, Table 2) and by boundaries established by peptide analysis (Supplemental Table ST3 in File S1). N_dom_2, like N_dom_1, begins at N_flex_, but ends further into tether at R_384_K, coinciding with the amino end of C_dom_2 (Tables 1 and 2). Corresponding polypeptides obtained from MALDI-TOF (Figure 3B, Supplemental Table ST1 in File S1) are L_28_–R_384_ (39,062 Da, 6.48 pI), K_26_–R_384_ (39,227 and 39,372 Da, 6.93 pI) and K_27_–K385 (40,061 Da, 7.3 pI). Peptide analysis (Supplemental Table ST3 in File S1) establishes consistent boundaries.

The amino end of N_dom_1 is at Y_17_ and its carboxy end is between K_385_ and K_402_ based on relative pI and apparent molecular weight (Figure 5) and peptide analysis (Supplemental Table ST3 in File S1), with a predicted mass of 42,589 Da and pI of 6.94 for the Y_17_–K_402_ species (Table 2). A corresponding peak was not observed in the MALDI-TOF spectra, however. Likewise, masses of 43,208 and 44,790 Da observed by MALDI-TOF (Figure 3B, Supplemental Table ST1 in File S1), corresponding to N_dom_ with trypsin cleavages at K_416_, K_418_, and K_433_ closer to or at the carboxy end of tether, were not confirmed by peptide analysis of N_dom_1 polypeptides from 2D gel spots. Heterogeneity within the N_dom_1 family and peptides sometimes too faintly detected to establish firm boundaries may explain this discrepancy between characterization of N_dom_1 polypeptides by 1D and 2D methods.

Like N_dom_, the C_dom_ family is divided into sub-families based on the length of tether retained. The amino end of C_dom_1 is at the N_dom_-tether boundary (K_348_ KTKL). The amino end of C_dom_2 (characterized above; Figure 6, Table 1) is at the major flexible site within tether (R_384_ KG). C_dom_ species with little or no tether was not found; on the other hand, spots derived from C_dom_2 were truncated within the carboxy domain (C_dom_Tr) at three possible internal sites (R_642_LK, R_657_, R_662_). Smaller polypeptides observed on 1D and 2D gels were not analyzed, as stable domain polypeptides unfold with decreasing size, exposing secondary cleavage sites.

### *D. melanogaster* tRNase Z variants and use of other proteases

Deletion of the FA of *D. melanogaster* tRNase Z causes a nearly two order of magnitude increase in *K*_m_ for tRNA processing with little effect on *k*_cat_, and the single substitution L187A at the boundary between the ascending stalk and the globular hand of the FA has almost the same effect [11]. These variants were therefore analyzed by limited proteolysis with trypsin and MALDI-TOF (Supplemental Figure SF4 and Supplemental Tables ST3 and ST4 in File S1). Detailed effects of tRNA binding on the FA of *B. subtilis* tRNase Z^8, cf 5^ are illustrated in Supplemental Figure SF5 in File S1. Wild type *D. melanogaster* tRNase Z was digested with endoproteinase LysC, which cleaves after lysines, and GluC, which cleaves after glutamates, followed by MALDI-TOF analysis (Supplemental Figure SF6 and Tables ST6 and ST7 in File S1).

### *H. sapiens* tRNase Z^L^

**Figure 7:**
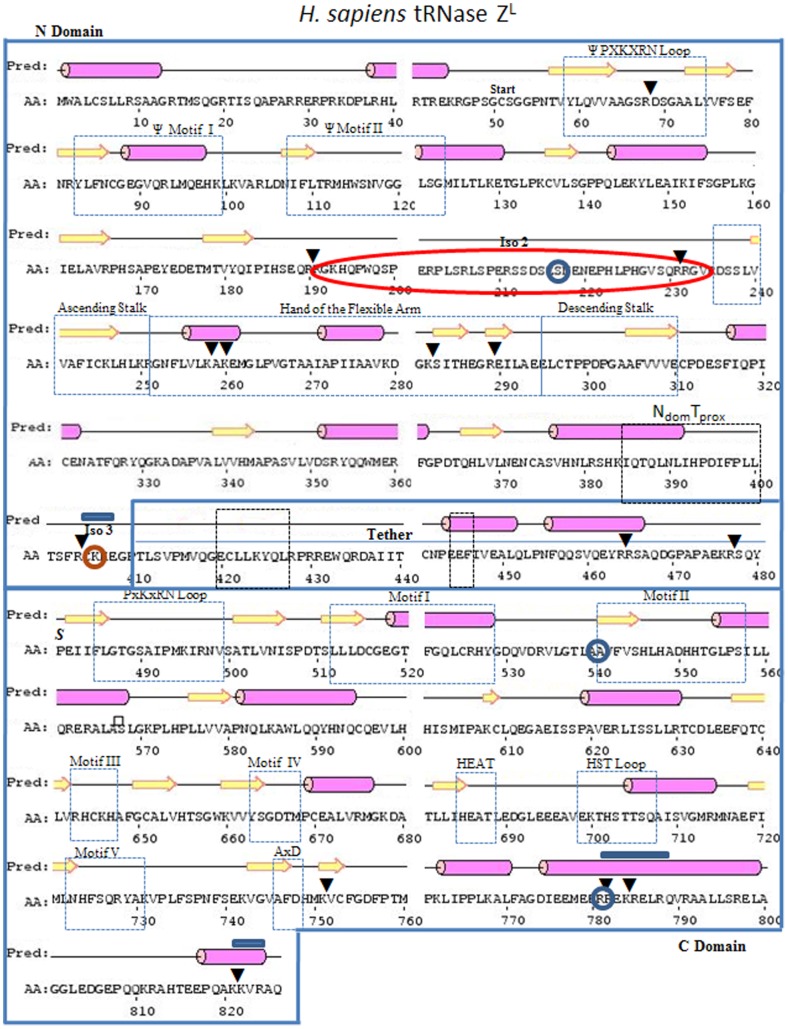
Sequence and predicted secondary structure of *H. sapiens* tRNase Z^L^ (Psipred). Enclosed regions N_dom_, Tether and C_dom_ and annotation of functional motifs are as in Figure 4. Polymorphisms and isoforms (identified by enclosure in blue circles and orange ellipses, respectively) are described in detail in Supplement.

**Figure 8:**
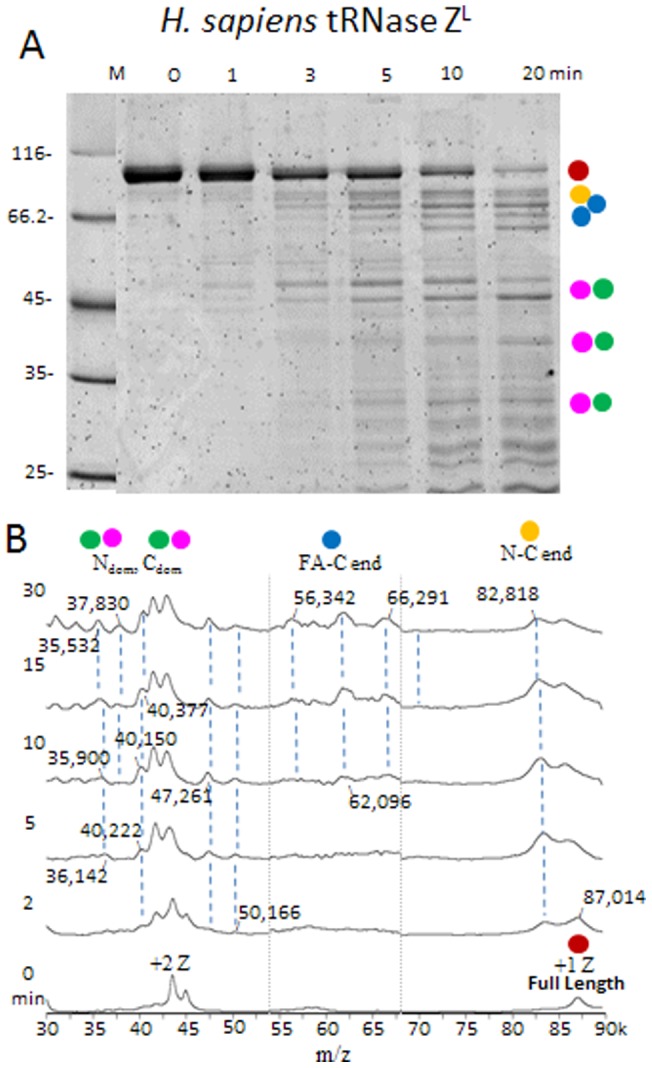
*H. sapiens* tRNase Z^L^ displays flexible regions like those in *D. melanogaster* tRNase Z. *H. sapiens* tRNase Z^L^ was expressed from G_50_ as previously described [17]. A) SDS-PAGE is the same as in Figure 3A. B) Presentation of the spectra and labeling of peaks is as in Figure 3B. The complete table is presented in Supplementary Table ST8.

**Figure 9:**
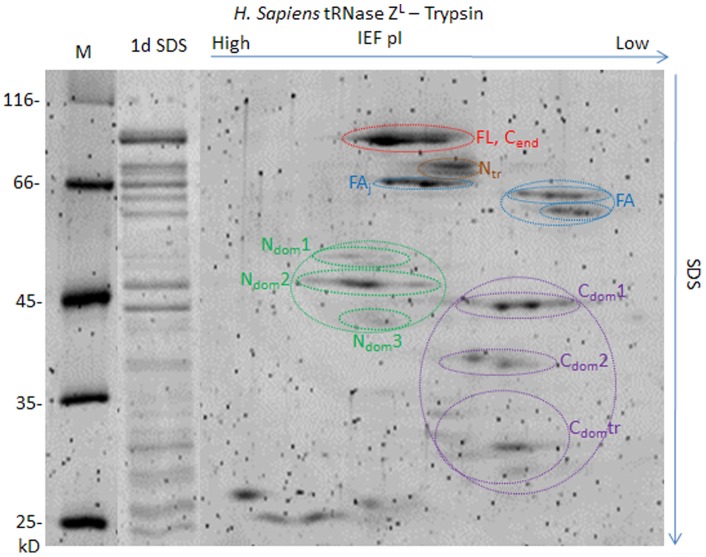
Two dimensional gel electrophoresis of *H. sapiens* tRNase Z^L^-trypsin polypeptides. A ten min. digest like those illustrated in Figure 5 was electrophoresed on a first dimension isoelectric focusing tube gel. The second dimension SDS-PAGE was as in Figure 5.

Because of differences in domain size and tether length between *D. melanogaster* tRNase Z and *H. sapiens* tRNase Z^L^ (Figure 1, cf Figures 4, 7), protease/MS spectra (Figure 8), 2D electrophoresis patterns (Figure 9) and polypeptide identification (Table 3, Supplemental Tables ST8, 9 in File S1) obtained from *H. sapiens* tRNase Z^L^ help evaluate the conservation of stable domains and flexible regions observed in *D. melanogaster* tRNase Z. A five residue leader (GAMGS) after TEV protease cleavage of the His-tag is followed by *H. sapiens* tRNase Z^L^ from G_50_ (***Start*** in Figure 7; see^17^). The major peak at 86,997 Da in the spectrum (Figure 8, Table 3, Supplemental Table ST8 in File S1) corresponds to the TEV cleaved product of *H. sapiens* tRNase Z^L^ (calc. 87,014 Da). A weak peak corresponds to tRNase Z^L^ with the uncleaved leader (mass observed 89,834 Da; calc. 89,854 Da).

**Table 3:** *H. sapiens* tRNase Z^L^ – Trypsin Polypeptides.

	Observed Polypeptide	Cleavage Sites		Theoretical		MALDI-TOF
		N-end	C-end	MW	pI	Peaks
***Full Length***	Full Length	N	Q_826_	86,997	6.62	87,004
***Flexible Carboxy End***	N_end_ – C_flex_	N	R_781_ REKRELR	82,844	6.53	82,818
	N_flex_ – C_flex_	RD_69_	R_781_ REKRELR	79,629–80,597	6.33–6.46	ND
***Amino End Truncation***	N_Tr_ – C_end_	RR_191_GKH	KK_822_VRAQ	70,683–71,025	6.40–6.55	ND
	N_Tr_ – C_end_	RR_191_GKH	KR_812_	69,108–69,450	6.31–6.45	ND
	N_Tr_ – C_flex_	RR_191_GKH	R_781_ REKRELR	65,695–67,203	6.24–6.53	66,258; 66,680
***Flexible Arm***	FA_J_ – C_end_	RR_232_G	KR_812_AHTEEPQAKKVRAQ	64,399–66,286	6.24–6.49	65,556–66,291
	FA_J_ – C_flex_	RR_232_G	R_781_ REKRELR	60,983–62,491	6.16–6.47	61,538; 61,907
	FA1 – C_end_	KA_259_ KE	KR_812_AHTEEPQAKKVRAQ	60,453–62,220	5.72–5.90	61,538
	FA2 – C_flex_	RS_283_	RREKR_785_	56,354	5.93	56,342
		RE_290_	ELRQVR_791_	56,355	5.97	
***Amino Domain***	N_dom_1	RD_69_	K_476_ R_477_	45,851–46,007	6.75, 6.99	ND
			R_464_ R_465_	44,642–44,798	6.75, 6.99	
	N_dom_2	N	RCKK_407_	39,774–40,133	6.99–7.74	40,150
***Carboxy Domain***	C_dom_1 – C_flex_	RCKKE_408_	RREKR_785_	41,735–42,664	5.89–6.26	41,748
	C_dom_2 – C_flex_	RR_465_S	RREKR_785_	34,848–35,574	5.95–6.25	35,551
			ELRQVR_791_	35,816–36,356		35,802; 36,218
	C_dom_2 – C_flex_	KRS_478_	RREKR_785_	33,640–34,366	5.95–6.25	ND
	C_dom_Tr	RR_465_S	K_751_	31,471; 31,627	6.35; 6.23	ND
	C_dom_Tr	KRS_478_	K_751_	30,419; 30,263	6.35; 6.22	ND

Interpretation of limited proteolysis products is based on gel electrophoresis (Figures 8A and 9) and mass spectrometry (Figures 8B and ST9).

Most peaks observed in the spectra (Figure 8) correspond to polypeptides that fit the designations developed for *D. melanogaster* tRNase Z (yellow highlighting in Supplemental Table ST8, cf ST1 in File S1). Cleavage was observed at R_68_ close to the amino end (N_flex_) and in tether, FA, and in the C-terminal tail (Table 3, cf Table 2).

Several peaks also arise from cleavage at a basic patch on the amino side of the *H. sapiens* tRNase Z^L^ FA referred to as N_Tr_ (truncated in amino domain at R_190_ RGK; Table 3, Supplemental Table ST8 in File S1). A tryptic fragment in *H. sapiens* tRNase Z^L^ beginning at R_231_ at the base of the ascending stalk referred to as FA_J_ (for example, a peak at 66,291 Da in the 30 minute time point interpreted as R_232_–Q_826_, calc. 66,286 Da; Figure 8, Supplemental Table ST8 in File S1) corresponds in position to a LysC cleavage in *D. melanogaster* tRNase Z (Supplemental Table ST6 in File S1) but was otherwise not prominently observed in *D. melanogaster* tRNase Z. These two basic flexible patches demarcate the boundaries encoded by exon 7 of *H. sapiens* tRNase Z^L^. Isoform 3 of *H. sapiens* tRNase Z^L^ arises from skipping of exon 7, causing deletion of 40 residues^41^ (orange ellipse in Figure 7).

Cleavage in the FA hand of *H. sapiens* tRNase Z^L^, but not the stalk, is observed at K_258_AKE, K_282_ and R_289_. A peak at 61,538 Da in the 15 min spectrum, for example, corresponds to A_259_–K_811_ with the correct pI [5.95], mass calc 61,504 Da; 56,342 Da in the 30 minute time point corresponds to the tryptic fragments S_283_–R_785_, calc. 56,354 Da, and E_290_–R_791_, calc. 56,355 Da, which cannot be distinguished from each other by mass or pI.

Two main N_dom_ species and two main C_dom_ species were observed. Prominent cleavages that demarcate amino and carboxy domains in *H. sapiens* tRNase Z^L^ include a basic patch in the amino domain proximal to tether (R_404_CKK), just as the accessible 348–351 basic patch in *D. melanogaster* tRNase Z is found on the amino side of tether (Figure 3–5). For example, a peak in the 10 minute spectrum at 47,261 Da corresponds to the proteolytic product C_405_–Q_826_, calc. 47,241 Da. Isoform 2 of *H. sapiens* tRNase Z^L^ (deletion of K_406_ or K_407_ within the flexible region at the N_dom_-tether boundary; orange circle in Figure 7) arises from slippage at the exon 13–14 splice junction [41].

Cleavages distributed through tether in *H. sapiens* tRNase Z^L^ include R_464_ RS and K_476_ RS (Figures 8, 9 and Supplemental Table ST8 in File S1), similar to the R_384_ KG region in *D. melanogaster* tRNase Z. Additionally, C_dom_Tr arises from cleavages in tether at R_464_ RS or K_476_ RS and within the carboxy domain at K_742_, K_751_, K_762_ or K_769_ (Figure 9 and Supplemental Tables ST8, ST9 in File S1).

The polypeptides whose ends define the stable domains and flexible regions in *H. sapiens* tRNase Z^L^, like those from *D. melanogaster* tRNase Z, fall into families when separated by two dimensional electrophoresis (Figure 9). Relative pI and apparent relative masses for the major polypeptide families and thus relative positions on the 2D gels are strikingly similar for these two long form tRNase Z species, with comparable coverage of the spots by mass spectrometry (Supplemental Figure SF9; cf SF3 in File S1; Appendix S2). Less heterogeneity is observed in the isoelectric focusing dimension, however, and a several-fold higher combination of trypsin concentration and incubation time are required for a comparable distribution of cleavage polypeptides with *H. sapiens* tRNase Z^L^ than with *D. melanogaster* tRNase Z.

N_dom_2 and C_dom_1, the most prominent N_dom_ and C_dom_ species, display a reversal of apparent relative molecular weights on the 2D gels. N_dom_2 migrates slower than C_dom_1 by SDS gel electrophoresis, while mass spectrometric methods show C_dom_1 and N_dom_2 to have masses in the 42 kDa and 40 kDa ranges, respectively; this discrepancy could be due to the higher pI of N_dom_ than C_dom_ polypeptides, consistent with the observation that positively charged proteins such as Ribonuclease A and histones migrate anomalously slow on SDS gels.

### Conserved characteristics of the tether

**Figure 10:**
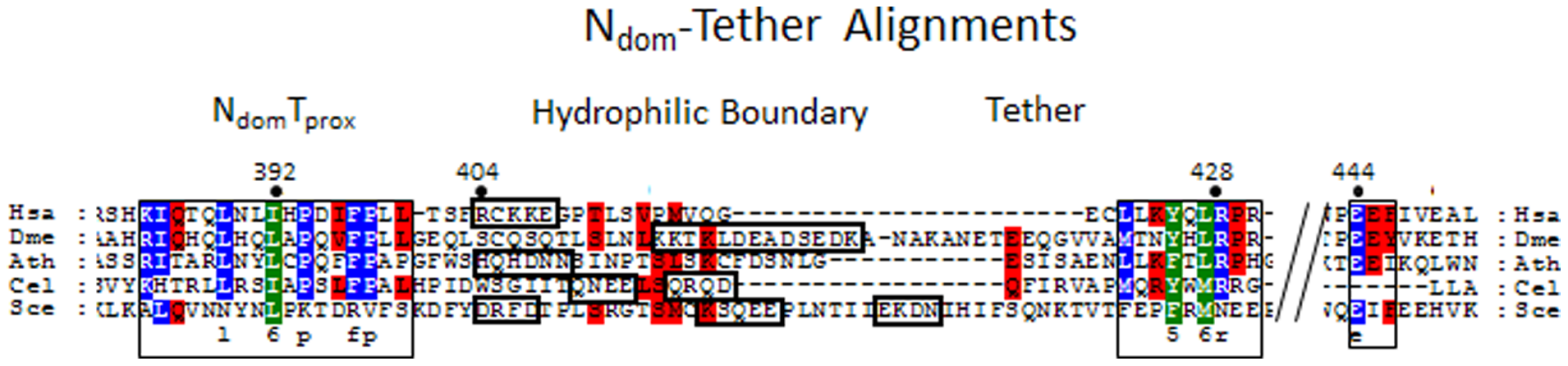
Tether alignments. The long form of tRNase Z from *H. sapiens* (Hsa; Accession # NP_060597), *D. melanogaster* (Dme; Q8MKW7), *A. thaliana* (Ath; AAM51378), *C. elegans* (Cel; O4476) and *S. cereviseae* (Sce; NP013005.1) were aligned using Clustalw. The full length alignments are presented in Supplemental Figure SF2 in File S1. Numbers at top are for *H. sapiens* tRNase ZL. Regions presented begin with the last identified homology block (N_dom_-T_prox_) in the amino domain [41] proximal to tether and include two smaller homology blocks in tether (rectangular enclosures).//indicates omitted sequence. Small bold rectangles enclose hydrophilic patches that mark the N_dom_-tether boundary.

Tether length, position and flexibility appear to be conserved between *D. melanogaster* tRNase Z and *H. sapiens* tRNase Z^L^. The carboxy boundary of the amino domain proximal to tether is demarcated by a conserved sequence block [41] designated N_dom_-T_prox_ (Figure 10). A hydrophilic region at the N_dom_-tether boundary (Figures 4 and 7; sequence enclosed in small bold rectangles in Figure 10 and Supplemental Figure SF2 in File S1) is similar to the hydrophilic patches close to the carboxy ends of metazoan tRNase Z^L^s. The carboxy boundary of tether and start of the alignment between C_dom_ and tRNase Z^S^ is marked by a conserved proline (S in Figures 4, 7; identical in 3/5 species in the full length alignments in Supplemental Figure SF2 in File S1and in 19/26 species in the alignment of metazoan tRNase Z^L^s [41]).

The tethers examined most closely here have similar sequences and predicted secondary structures (two α-helices toward the carboxy end; Figures 4 and 7). Two short sequence patches are conserved in the first half of tether: BxKBxBRP (in which B is a bulky hydrophobic residue) just preceding the major flexible site RK_385_ in the *D. melanogaster* tRNase Z tether and EE_399_B at the start of the first predicted α helix. The first of these sites is detected as a possible trypsin cleavage at K_424_ in the *H. sapiens* tRNase Z^L^ C_dom_ polypeptide Y_425_–K_784_ (Supplemental Table ST8 in File S1); the second is detected by GluC in *D. melanogaster* C_dom_ polypeptide E_399_–E_718_ (Supplemental Table ST7 in File S1).

## Discussion

**Figure 11:**
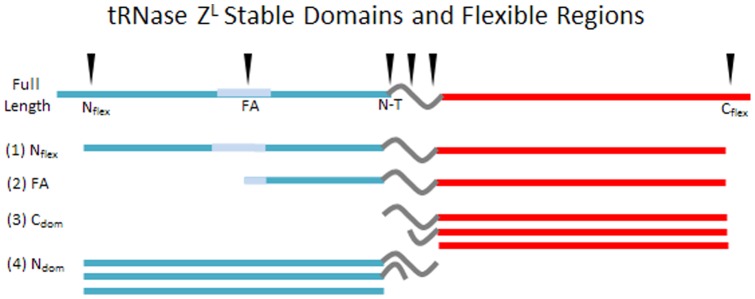
Tethered domains and flexible regions in tRNase Z^L^. Polypeptides characterized in the limited proteolysis experiments define the stable domains and flexible regions in tRNase Z^L^ illustrated in the diagram. Most of the proteolytic cleavages fall into four flexible regions, designated N_flex_, FA, Tether and C_end_, producing polypeptides designated N_flex_, FA, N_dom_ and C_dom_. Two main flexible regions in tether produce N_dom_ and C_dom_ polypeptides varying in length as illustrated.

Two defining features of tRNase Z^L^, flexible tethering of tandemly duplicated, functionally diverged domains and the presence of the tRNA-binding flexible arm in the amino domain, are directly characterized here through biophysical analysis. tRNase Z^L^ purifies under native conditions and presumably functions as a monomer, while tRNase Z^S^ is a homodimer. The main flexible regions are the FA hand and the tether that links the amino and carboxy domains, besides the hydrophilic ends, illustrated schematically in Figure 11. Internal sites presented in Tables 2 and 3 and Figures 4 and 7, including N_Int_ and C_Tr_ in *D. melanogaster* tRNase Z and N_tr_, FA_J_ and C_dom_Tr in *H. sapiens* tRNase Z^L^, are not equivalent.

### Simultaneous vs. sequential cleavage in flexible regions and production and disappearance of stable domains

Data from the time courses with trypsin and other proteases generally favor simultaneous, as distinguished from strictly sequential, cleavage of tRNase Z. 1D SDS-PAGE and MALDI-TOF experiments show polypeptide species that appear early and persist late, over a factor of ten in digestion time (from 1–10 min for *D. melanogaster* tRNase Z and from 3–30 min for *H. sapiens* tRNase Z^L^), and similar overall patterns were observed in time courses of tRNase Z^L^ cleavage by trypsin separated on 2D gels (not shown).

### Depletion of N_dom_ but not C_dom_ polypeptides by FA cleavage

A tRNase Z protein cleaved in FA would not be a source for N_dom_ polypeptides, but could produce C_dom_ polypeptides. Similarly, once cleaved in tether, the pool of N_dom_ polypeptides could be depleted by cleavage in FA, producing fragments below 30–35 kDa, too small for productive analysis of stable domains by these methods. C_dom_Tr fragments are the only small stable polypeptides.

### Inverse relation between flexibility and catalytic efficiency

*D. melanogaster* tRNase Z requires ∼5-fold less time/protease to achieve similar levels of proteolysis, and is therefore more flexible than *H. sapiens* tRNase Z^L^ *D. melanogaster* tRNase Z is between 10 – 100-fold less active in pre-tRNA processing than *H. sapiens* tRNase Z^L^ (from comparisons between [11], [17], [37], [42]), suggesting an inverse correlation between flexibility and catalytic efficiency. Likewise, the *D. melanogaster* L187A tRNase Z variant displays a more flexible FA hand (Supplemental Figure SF4B in File S1; cf Figure 3B) and close to 100-fold lower catalytic efficiency than wild type due to an increase in *K*_m_, suggesting impaired substrate binding. Hypotheses relating flexibility to catalytic efficiency are more fully developed in Supplement.

### Possible adaptive value of tandem duplication with a flexible tether

Strong N_dom_ and C_dom_ peaks observed throughout the tRNase Z^L^ time course with trypsin and other proteases support the tandem duplication/flexible tether model of eukaryotic tRNase Z evolution. tRNase Z^L^ is generally more than twice the size of the tRNase Z^S^ subunit (e.g. 826 residues, 92,219 Da in *H. sapiens* tRNase Z^L^ vs 363 residues, 40,019 Da in tRNase Z^S^). tRNase Z^S^, a homodimeric protein, requires an extensive dimer interface. In contrast, the amino- and carboxy-domains of tRNase Z^L^ are flexibly tethered. Close to 2,000-fold greater catalytic efficiency of *H. sapiens* tRNase Z^L^ over tRNase Z^S^ is consistent with adaptive value of the flexible tether.

## Supporting Information

Appendix S1 **MALDI-Ion trap MS/MS spectra from** ***D. melanogaster*** **tRNAseZ.**
(PDF)

Appendix S2 **MALDI-Ion trap MS/MS spectra from** ***H. sapiens*** **tRNAseZ.**
(PDF)

File S1 **Contains supplemental figures SF1 – SF7, and supplemental tables ST1- ST9 and acc:ompanying text:** Figure SF1. tRNA in a complex with tRNase Z^S^. Figure SF2. Full length tRNase Z^L^ alignments. Table ST1. Wild Type *D. melanogaster* tRNase Z – Trypsin MALDI-ToF based on spectra in Figure 3B. Table ST2. Predicted members of *D. melanogaster* tRNase Z C_dom_2 family. Figure SF3. *D. melanogaster* tRNase Z – Trypsin Full Length Coverage. Table ST3. Compilation of *D. melanogaster* tRNase Z peptide MALDI-Ion trap. Figure SF4. *D. melanogaster* tRNase Z ΔFA & L187A MALDI-ToF. Tables ST4, ST5. Polypeptide Tables based on spectra in Supplemental Figure SF4. Figure SF5. Detail views of tRNase Z FA free and bound to tRNA. Figure SF6. *D. melanogaster* tRNase Z– LysC & GluC MALDI-ToF. Tables ST6, ST7. Polypeptide Tables based on spectra in Supplemental Figure SF6. Table ST8. *H. sapiens* tRNase Z^L^ – Trypsin MALDI-ToF Table. Figure SF7. *H. sapiens* tRNase Z^L^ – Trypsin Full Length Coverage. Table ST9. Compilation of *H. sapiens* tRNase Z^L^ peptide MALDI-Ion trap.
(DOCX)

## References

[1] Hartmann RK, Gössringer M, Späth B, Fischer S, Marchfelder A (2009) The making of tRNAs and more – RNase P and tRNase Z. Prog Mol Biol Transl Sci. 85: 319–368. 10.1016/S0079-6603(08)00808-8 19215776

[2] McClain WH, Guerrier-Takada C, Altman S (1987) Model substrates for an RNA enzyme. Science 238: 527–530. 2443980 10.1126/science.2443980

[3] Levinger L, Bourne R, Kolla S, Cylin E, Russell K, et al (1998) Matrices of paired substitutions show the effects of tRNA D/T loop sequence on Drosophila RNase P and 3'-tRNase processing. J Biol Chem 273: 1015–1025. 9422763 10.1074/jbc.273.2.1015

[4] Shi PY, Weiner AM, Maizels N (1998) A top-half tDNA minihelix is a good substrate for the eubacterial CCA-adding enzyme. RNA 4: 276–284. 9510330 PMC1369617

[5] Li de la Sierra-Gallay I, Pellegrini O, Condon C (2005) Structural basis for substrate binding, cleavage and allostery in the tRNA maturase RNase Z. Nature. 433: 657–661. 10.1038/nature03284 15654328

[6] Ishii R, Minagawa A, Takaku H, Takagi M, Nashimoto M, et al (2005) Crystal structure of the tRNA 3' processing endoribonuclease tRNase Z from Thermotoga maritima. J Biol Chem 280: 14138–14144. 15701599 10.1074/jbc.M500355200

[7] Schilling O, Späth B, Kostelecky B, Marchfelder A, Meyer-Klaucke W, et al (2005) Exosite modules guide substrate recognition in the ZiPD/ElaC protein family. J Biol Chem 280: 17857–17862. 15699034 10.1074/jbc.M500591200

[8] Kostelecky B, Pohl E, Vogel A, Schilling O, Meyer-Klaucke W (2006) The crystal structure of the zinc phosphodiesterase from Escherichia coli provides insight into function and cooperativity of tRNase Z-family proteins. J Bacteriol. 188: 1607–1614. 10.1128/JB.188.4.1607-1614.2006 PMC1367222 16452444

[9] Li de la Sierra-Gallay I, Mathy N, Pellegrini O, Condon C (2006) Structure of the ubiquitous 3′ processing enzyme RNase Z bound to transfer RNA. Nat Struct Mol Biol 13: 376–377. 16518398 10.1038/nsmb1066

[10] Ishii R, Minagawa A, Takaku H, Takagi M, Nashimoto M, et al (2007) The structure of the flexible arm of Thermotoga maritima tRNase Z differs from those of homologous enzymes. Acta Crystallogr 63: 637–641. 10.1107/S1744309107033623 PMC2335171 17671357

[11] Levinger L, Hopkinson A, Desetty R, Wilson C (2009) Effect of changes in the flexible arm on tRNase Z processing kinetics. J Biol Chem 284: 15685–15691. 19351879 10.1074/jbc.M900745200 PMC2708865

[12] Pellegrini O, Li de la Sierra-Gallay I, Piton J, Gilet L, Condon C (2012) Activation of tRNA maturation by downstream uracil residues in B. subtilis. Structure. 20: 1769–77. 10.1016/j.str.2012.08.002 22940585

[13] Aravind L (1999) An evolutionary classification of the metallo-beta-lactamase fold proteins. In Silico Biol 1: 69–91. 11471246

[14] Dominski Z (2007) Nucleases of the metallo-β-lactamase family and their role in DNA and RNA metabolism. Crit Rev Biol & Mol Biol 42: 67–93. 10.1080/10409230701279118 17453916

[15] Schiffer S, Rosch S, Marchfelder A (2002) Assigning a function to a conserved group of proteins: the tRNA 3'-processing enzymes. EMBO J 21: 2769–2777. 12032089 10.1093/emboj/21.11.2769 PMC126033

[16] Dubrovsky EB, Dubrovskaya VA, Levinger L, Schiffer S, Marchfelder A (2004) *Drosophila* RNase Z processes mitochondrial and nuclear pre-tRNA 3' ends *in vivo* . Nucleic Acid Res 32: 255–262. 14715923 10.1093/nar/gkh182 PMC373292

[17] Yan H, Zareen N, Levinger L (2006) Naturally occurring mutations in human mitochondrial pre-tRNA^Ser(UCN)^ can affect the tRNase Z cleavage site, processing kinetics and substrate secondary structure. J Biol Chem 281: 3926–3935. 16361254 10.1074/jbc.M509822200

[18] Mineri R, Pavelka N, Fernandez-Vizarra E, Ricciardi-Castagnoli P, Zeviani M, et al (2009) How do human cells react to the absence of mitochondrial DNA? PLoS One 4: e5713. 19492094 10.1371/journal.pone.0005713 PMC2683933

[19] Rossmanith W (2011) Localization of human RNase Z isoforms: dual nuclear/mitochondrial targeting of the ELAC2 gene product by alternative translation initiation. PLoS One 6: e19152. 21559454 10.1371/journal.pone.0019152 PMC3084753

[20] Brzezniak LK, Bijata M, Szczesny RJ, Stepien PP (2011) Involvement of human ELAC2 gene product in 3' end processing of mitochondrial tRNAs. RNA Biol 8: 616–626. 21593607 10.4161/rna.8.4.15393

[21] Zareen N, Yan H, Hopkinson A, Levinger L (2005) Residues in the conserved His domain of fruit fly tRNase Z that function in catalysis are not involved in substrate recognition or binding. J Mol Biol 350: 189–199. 15935379 10.1016/j.jmb.2005.04.073

[22] Tavtigian SV, Simard J, Teng DHF, Abtin V, Baumgard M, et al (2001) A candidate prostate cancer susceptibility gene at chromosome 17p. Nature Genet 27: 172–180. 11175785 10.1038/84808

[23] Redko Y, Li de la Sierra-Gallay I, Condon C (2007) When all's zed and done: the structure and function of RNase Z in prokaryotes. Nat Rev Microbiol 5: 278–286. 17363966 10.1038/nrmicro1622

[24] Novotny J, Bruccoleri RE (1987) Correlation among sites of limited proteolysis, enzyme accessibility and segmental mobility. FEBS Lett 211: 185–189. 3542567 10.1016/0014-5793(87)81433-3

[25] Kazanov MD, Igarashi Y, Eroshkin AM, Cieplak P, Ratnikov B, et al (2011) Structural Determinants of Limited Proteolysis. J Proteome Res 10: 3642–3651. 21682278 10.1021/pr200271w PMC3164237

[26] Fontana A, de Laureto PP, Spolaore B, Frare E, Picotti P, et al (2004) Probing protein structure by limited proteolysis. Acta Biochim Pol 51: 299–321. 15218531

[27] Fontana A, Fassina G, Vita C, Dalzoppo D, Zamai M, et al (1986) Correlation between sites of limited proteolysis and segmental mobility in thermolysin. Biochemistry 25: 1847–1851. 3707915 10.1021/bi00356a001

[28] Herschlag D (1988) The role of induced fit and conformational changes of enzymes in specificity and catalysis. Bioorganic Chem 16: 62–96.

[29] Packman LC, Perham RN (1987) Limited proteolysis and sequence analysis of the 2-oxo acid dehydrogenase complexes from Escherichia coli. Cleavage sites and domains in the dihydrolipoamide acyltransferase components. Biochem J 242: 531–538. 3297046 10.1042/bj2420531 PMC1147738

[30] Baker ES, Luckner SR, Krause KL, Lambden PR, Clarke IN, et al (2012) Inherent Structural Disorder and Dimerisation of Murine Norovirus NS1-2 Protein. PLoS One 7: e30534. 22347381 10.1371/journal.pone.0030534 PMC3274520

[31] Arighi CN, Rossi JP, Delfino JM (2003) Temperature-induced conformational switch in intestinal fatty acid binding protein (IFABP) revealing an alternative mode for ligand binding. Biochemistry 42: 7539–7551. 12809510 10.1021/bi020680d

[32] Sajnani G, Pastrana MA, Dynin I, Onisko B, Requena JR (2008) Scrapie prion protein structural constraints obtained by limited proteolysis and mass spectrometry. J Mol Biol 382: 88–98. 18621059 10.1016/j.jmb.2008.06.070

[33] Cohen SL, Chait BT (2001) Mass spectrometry as a tool for protein crystallography. Annu Rev Biophys Biomol Struct. 30: 67–85. 10.1146/annurev.biophys.30.1.67 11340052

[34] Villanueva J, Villegas V, Querol E, Avilés FX, Serrano L (2002) Protein secondary structure and stability determined by combining exoproteolysis and matrix-assisted laser desorption/ionization time-of-flight mass spectrometry. J Mass Spectrom. 37: 974–984. 10.1002/jms.356 12271440

[35] Karkashon S, Hopkinson A, Levinger L (2007) tRNase Z Catalysis and Conserved Residues on the Carboxy Side of the His Cluster. Biochemistry 46: 9380–9387. 17655328 10.1021/bi700578v PMC2526284

[36] Cadene M, Chait BT (2000) A robust, detergent-friendly method for mass spectrometric analysis of integral membrane proteins. Anal. Chem. 72: 5655–5658. 10.1021/ac000811l 11101244

[37] Fenyo D, Wang Q, DeGrasse JA, Padovan JC, Cadene M, et al (2007) MALDI sample preparation: the ultrathin layer method. J. Vis. Exp. 3: 192. 10.3791/192 PMC2535834 18978997

[38] Malone JP, Radabaugh MR, Leimgruber RM, Gerstenecker GS (2001) Practical aspects of fluorescent staining for proteomic applications. Electrophoresis. 22: 919–932. 10.1002/1522-2683()22:5<919::AID-ELPS919>3.0.CO;2-U 11332760

[39] Chang EJ, Archambault V, McLachlin DT, Krutchinsky AN, Chait BT (2004) Analysis of Protein Phosphorylation by Hypothesis-Driven Multi-Stage Mass Spectrometry. Anal Chem. 76: 4472–4483. 10.1021/ac049637h 15283590

[40] Kalkum M, Lyon GJ, Chait BT (2003) Detection of secreted peptides by using hypothesis-driven multistage mass spectrometry. Proc Natl Acad Sci USA 100: 2795–2800. 12591958 10.1073/pnas.0436605100 PMC151420

[41] Wang Z, Zheng J, Zhang X, Peng J, Liu J, et al (2012) Identification and sequence analysis of metazoan tRNA 3'-end processing enzymes tRNase Zs. PLoS One 7(9): e44264 doi: 10.1371/journal.pone.0044264. Epub 2012 Sep 4 22962606 10.1371/journal.pone.0044264 PMC3433465

[42] Hopkinson A, Levinger L (2008) Effects of conserved D/T loop substitutions in the pre-tRNA substrate on tRNase Z catalysis. RNA Biol 5: 104–111. 18421255 10.4161/rna.5.2.6086

[43] Dubrovsky EB, Dubrovskaya VA, Bilderback AL, Berger EM (2000) The isolation of two juvenile hormone-inducible genes in Drosophila melanogaster. Dev Biol. 224: 486–495. 10.1006/dbio.2000.9800 10926782

[44] Takaku H, Minagawa A, Takagi M, Nashimoto M (2003) A candidate prostate cancer susceptibility gene encodes tRNA 3' processing endoribonuclease. Nucleic Acids Res. 31: 2272–2278. 10.1093/nar/gkg337 PMC154223 12711671

