# Supplementary material for: Tethered Domains and Flexible Regions in tRNase ZL, the Long Form of tRNase Z
Source: PLoS One. 2013 Jul 17;8(7):e66942. doi: 10.1371/journal.pone.0066942 (PMC3714273; doi:10.1371/journal.pone.0066942)

D. Melanogaster tRNase Z

Supplemental Appendix 1. Panels 1-40 provide typical examples of MALDI-ion trap MS/MS spectra that confirm the amino acid sequences of the *D. melanogaster* tRNaseZ peptides obtained by exhaustive digestion of 2D gel spots as represented in Supplemental Table ST3. Peaks are labeled in red, mainly as b-ion and c-ion derivatives (N-terminal fragments, numbering from N-terminal end, with cleavage at the peptide bond and c-terminal to peptide bond, respectively), and y-ion derivatives (C-terminal fragments, numbering from C-terminal end, cleavage at peptide bond). Theoretical m/z values are shown in parentheses. In certain cases, more than 1 theoretical tryptic peptide had a nominal molecular weight consistent with the isolated *m/z* value, and both peptides were considered (panels 19/20, and 34/35). Fragmentation results favor 20 over 19 and 35 over 34. Cysteines are modified *in situ* with acrylamide from the 2D gel preparation, to produce S-proprionamidated species). Under MS/MS conditions, these sometimes gave rise to neutral loss products where the cysteine sulfur and proprionamide modification are lost as a formal C_3H_6ONS species (*e.g.* panels 13,15). One species was present as both an unmodified species, and presumably cyclized N-terminal pyroglutamate species (panels 35/36). The unmodified species is less abundant, and it's low intensity MS/MS spectrum is consistent with the more abundant modified species. Results of panel 35 therefore support the interpretation in panel 36. All assignments were made manually.

1. MS^2 m/z 605

Theoretical mass: 604.40 Da 132-136, *D. melanogaster*

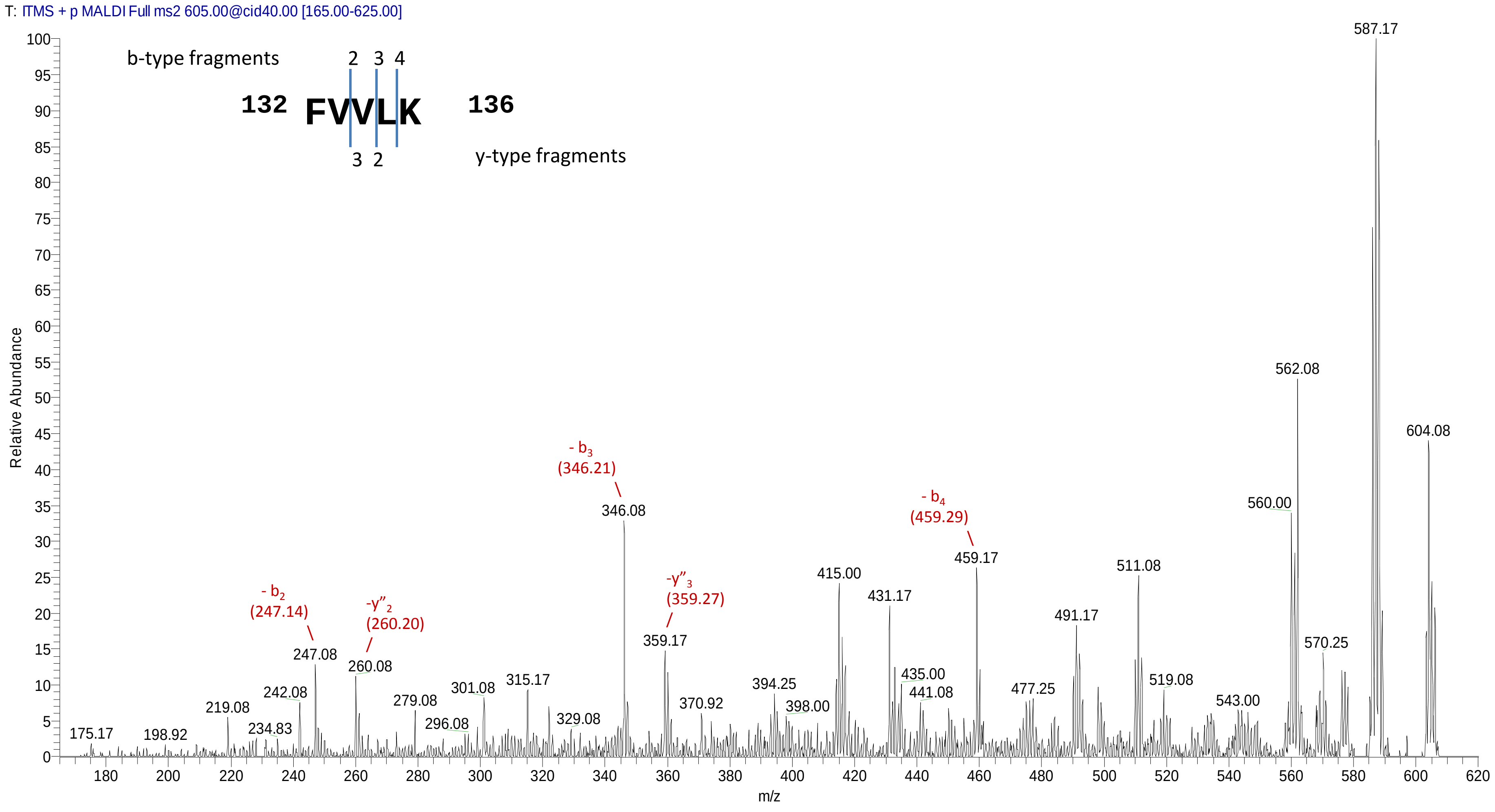

2. MS^2 m/z 727

Theoretical mass: 726.402 Da 20-25, *D. melanogaster*

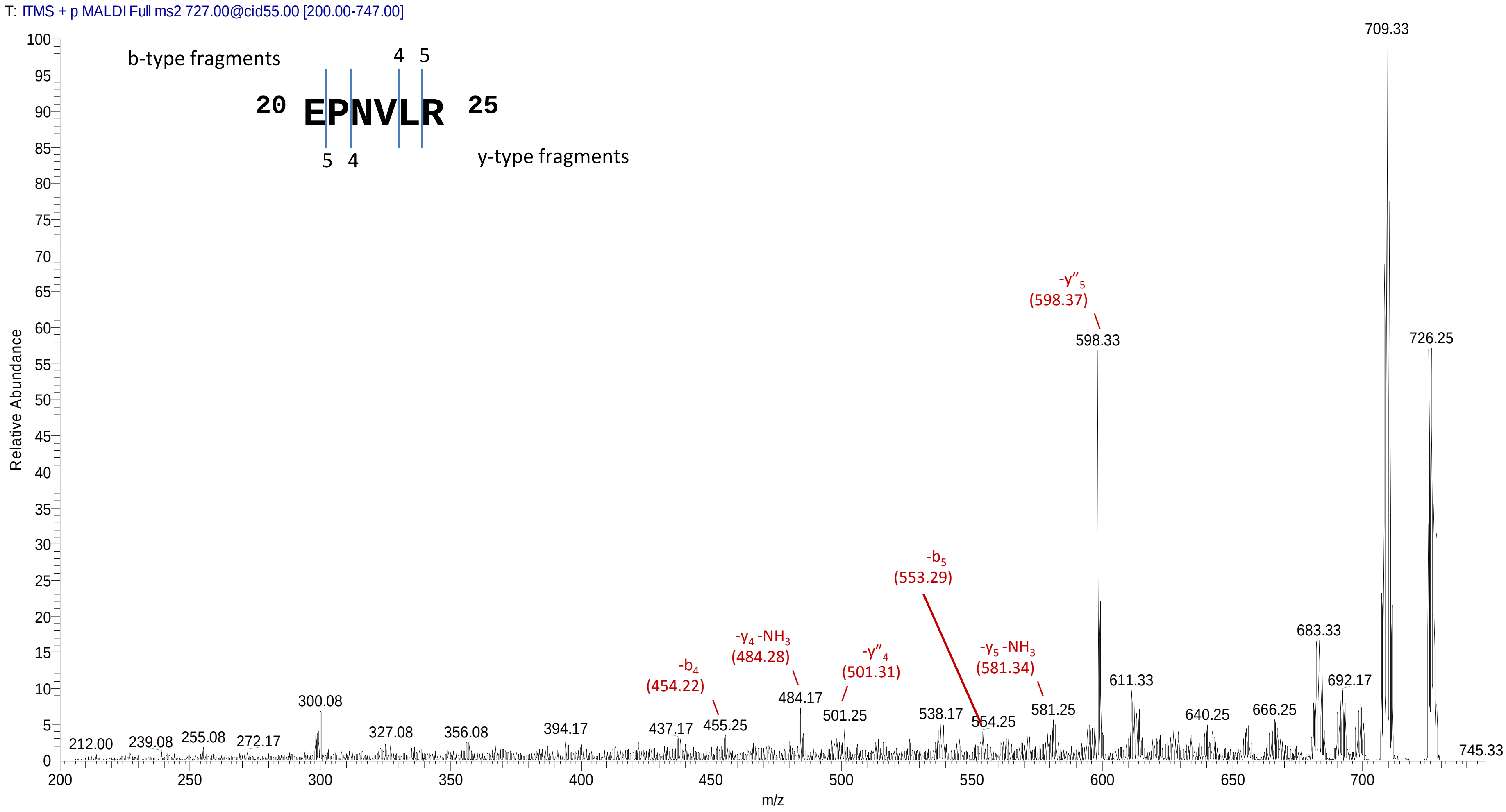

3. MS^2 m/z 734

Theoretical mass: 733.39 Da 72-77, *D. melanogaster*

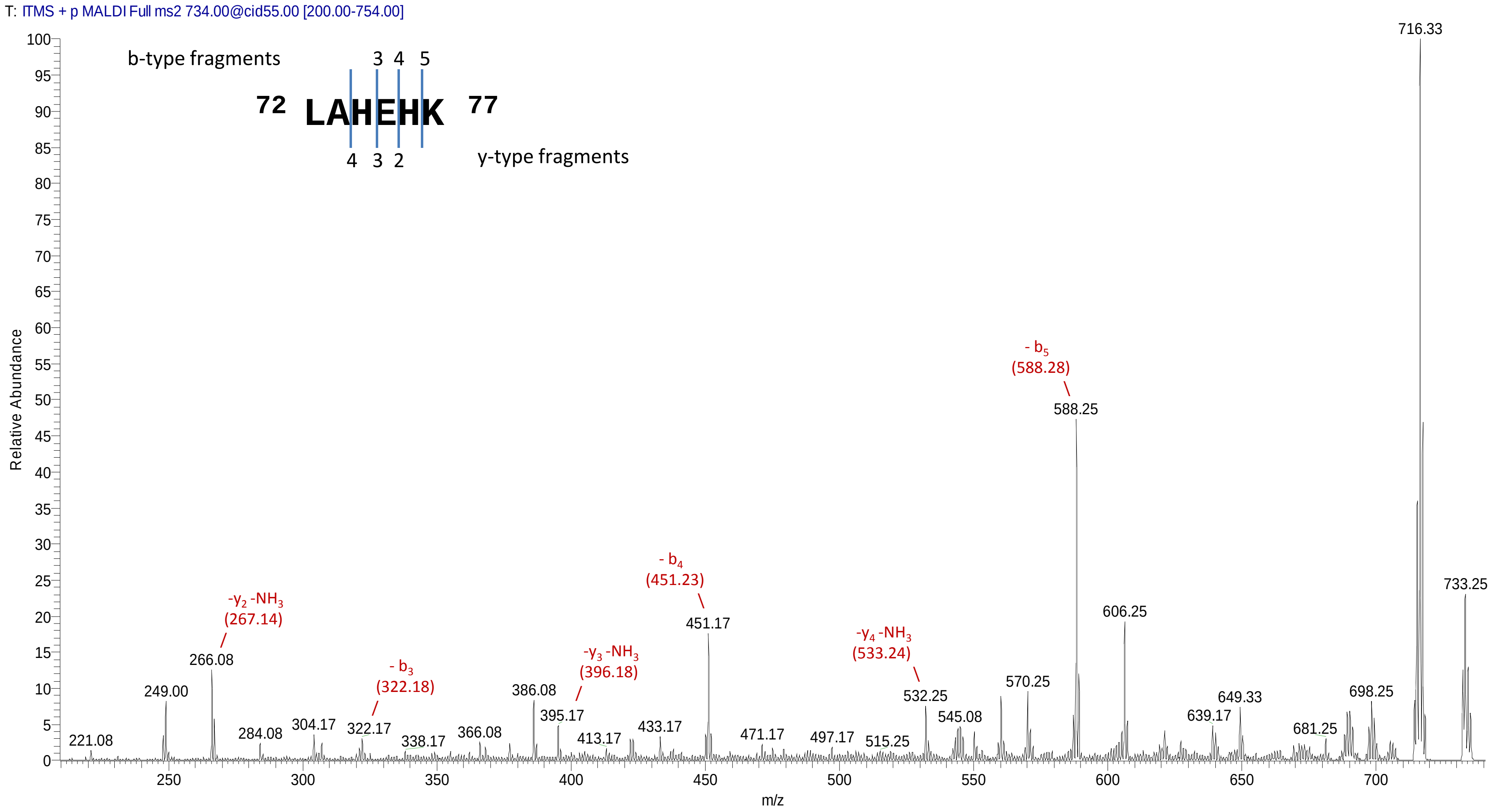

4. MS^2 m/z 755

Theoretical mass: 754.40 Da 10-16, *D. melanogaster*

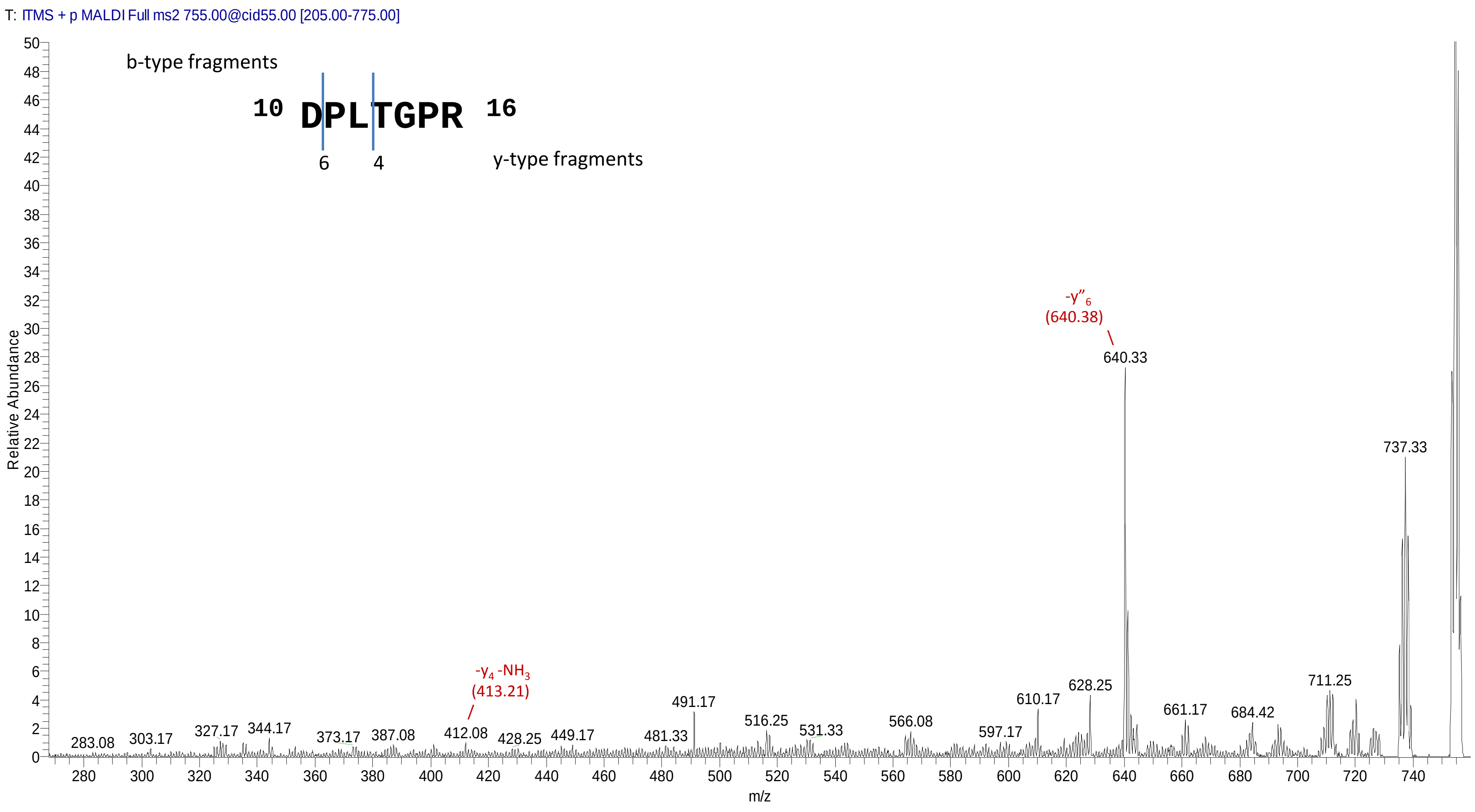

5. MS^2 m/z 761

Theoretical mass: 760.50 Da 131-136, *D. melanogaster*

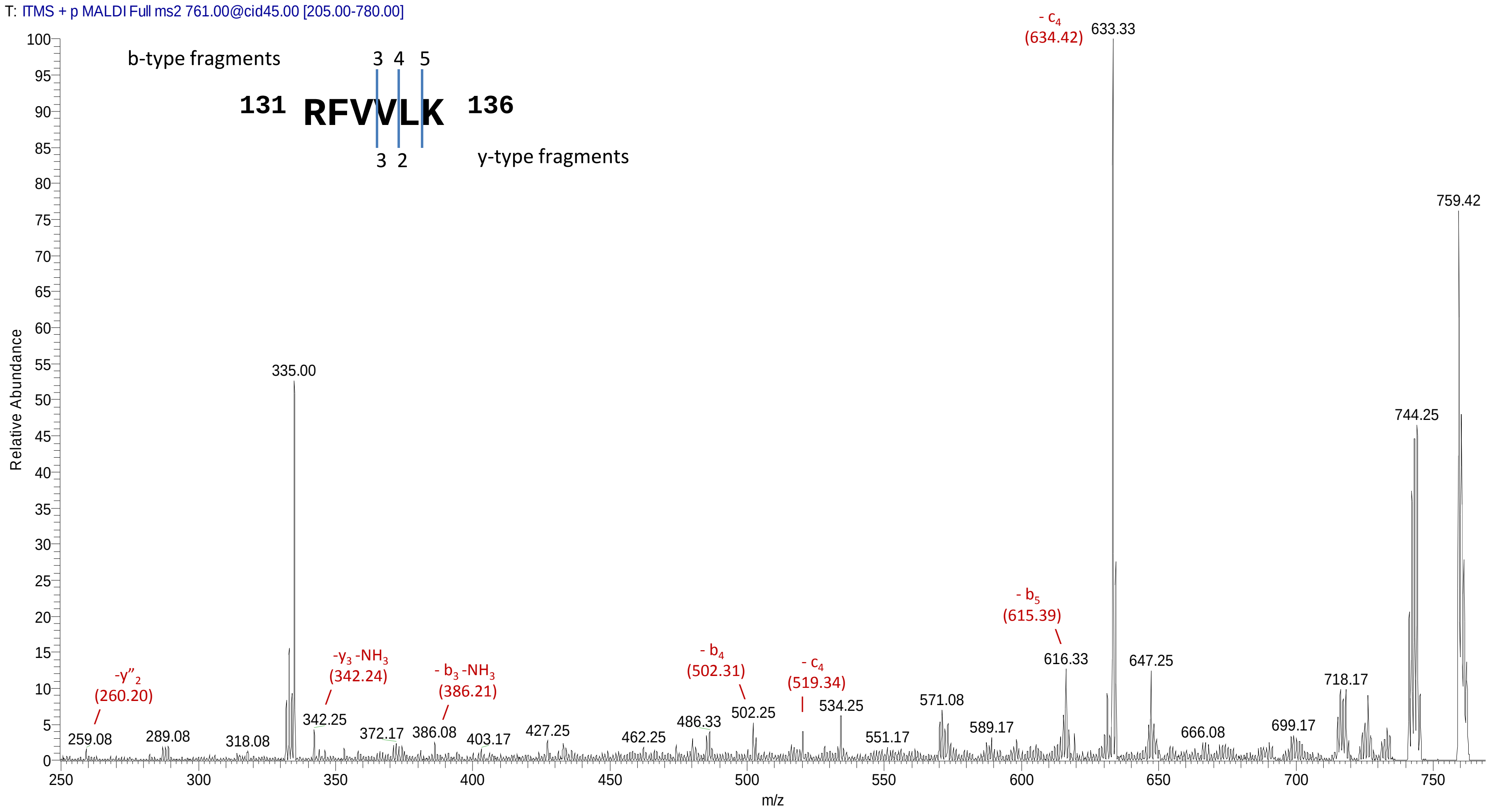

6. MS^2 m/z 797

Theoretical mass: 796.50 Da 515-520, *D. melanogaster*

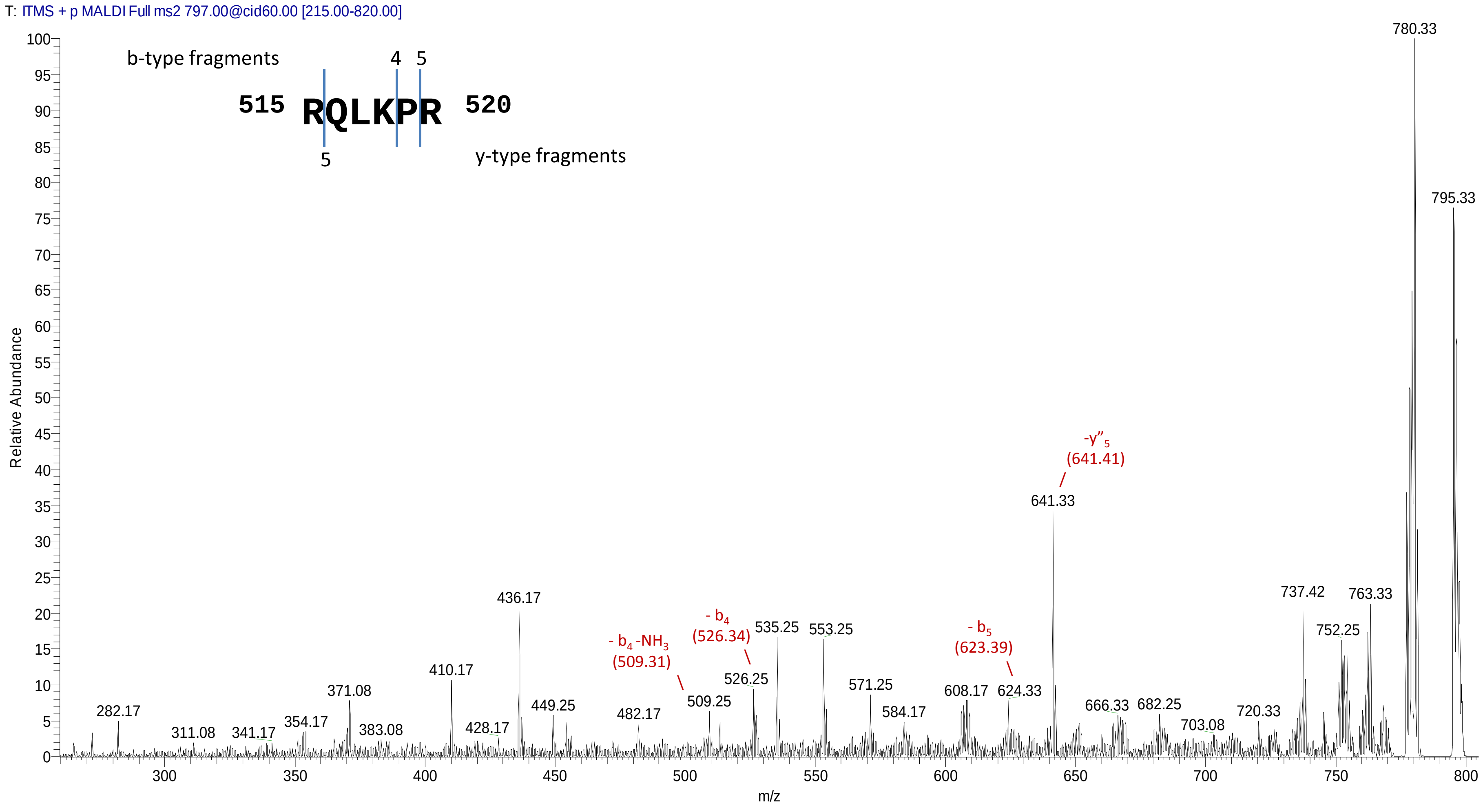

7. MS^2 m/z 901

Theoretical mass: 900.54 Da 515-520, *D. melanogaster*

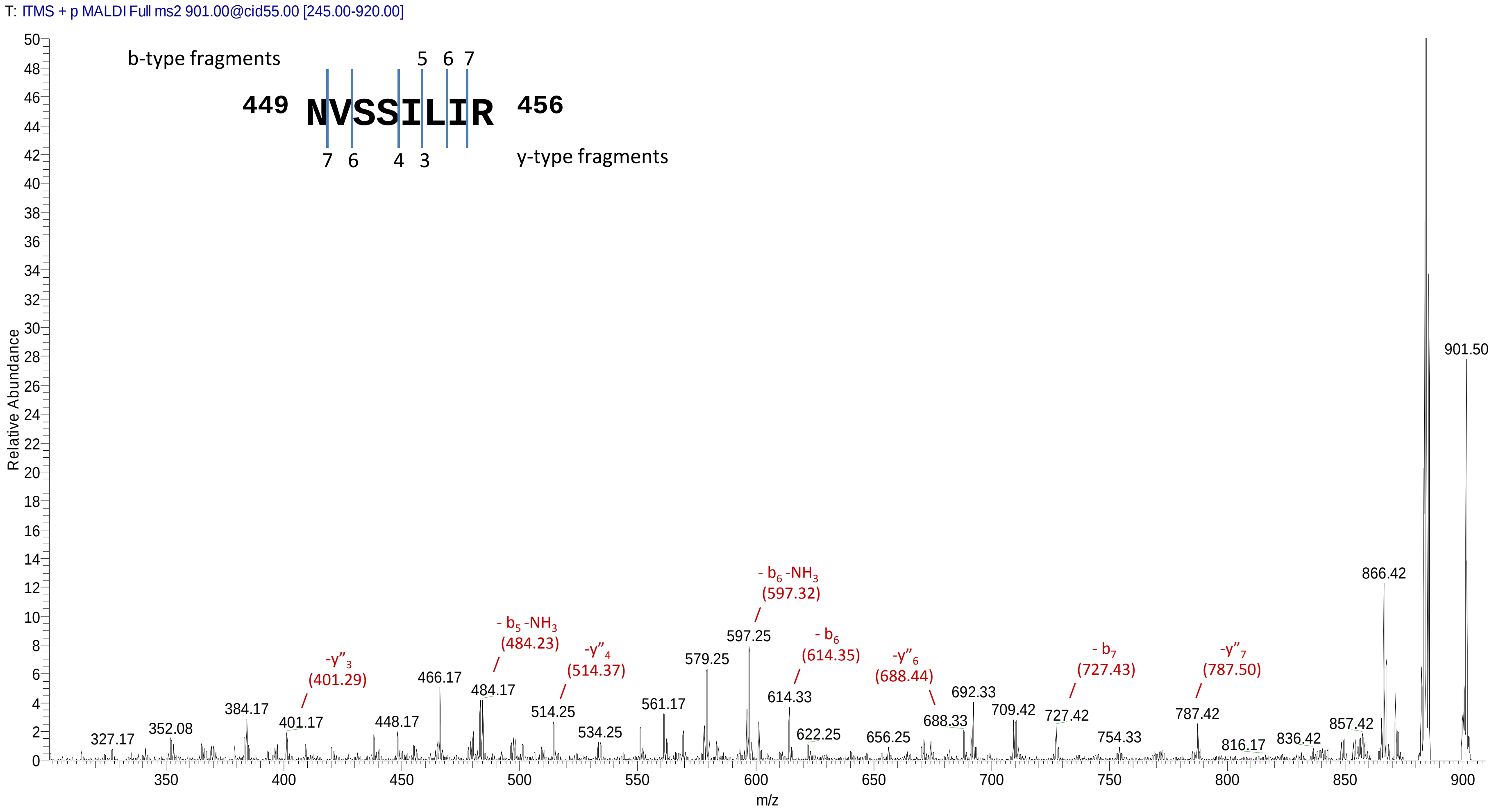

8. MS^2 m/z 978

Theoretical mass: 977.51 Da 395-402, *D. melanogaster*

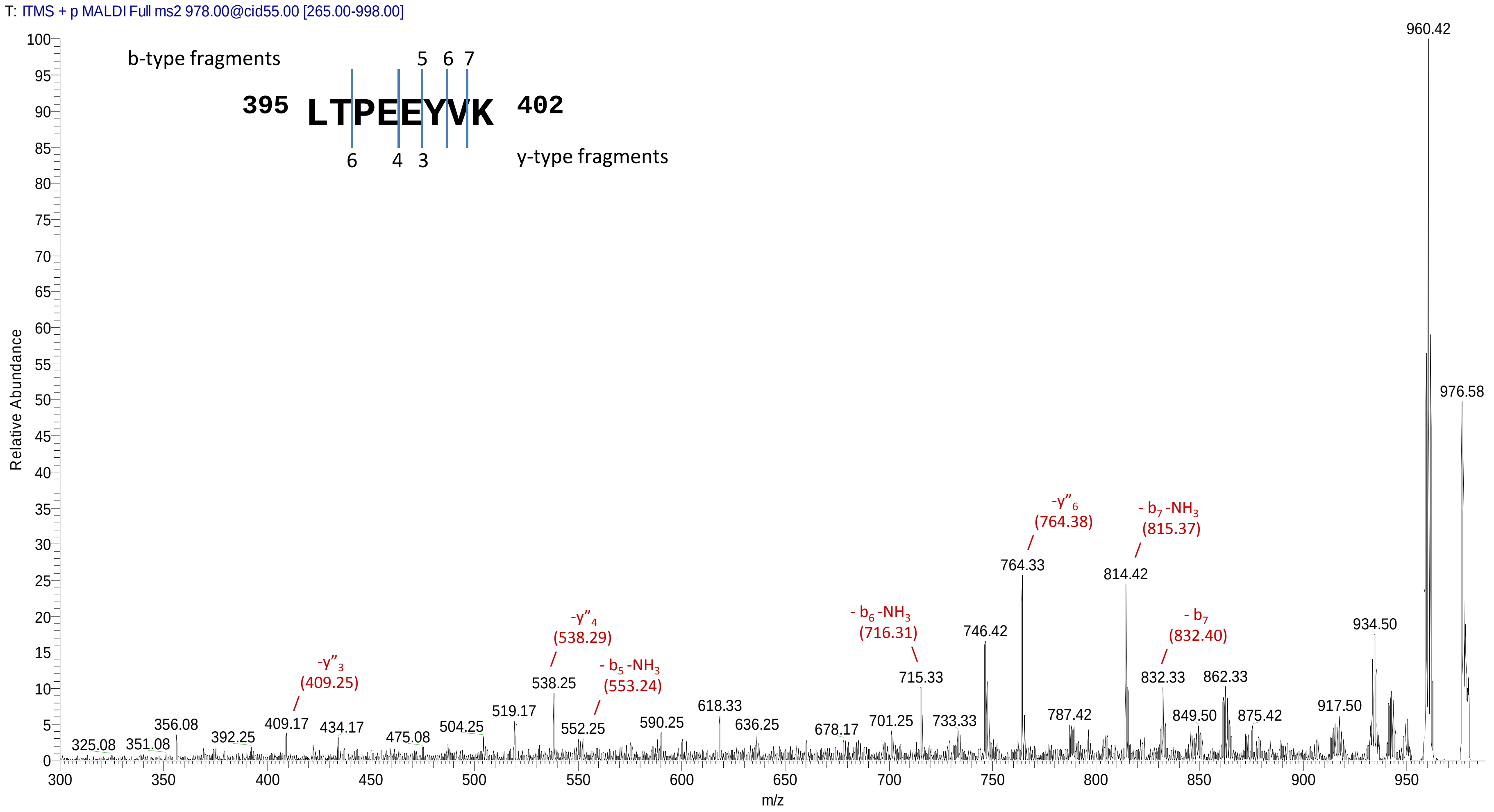

9. MS^2 m/z 1077

Theoretical mass: 1077.65 Da 521-530, *D. melanogaster*

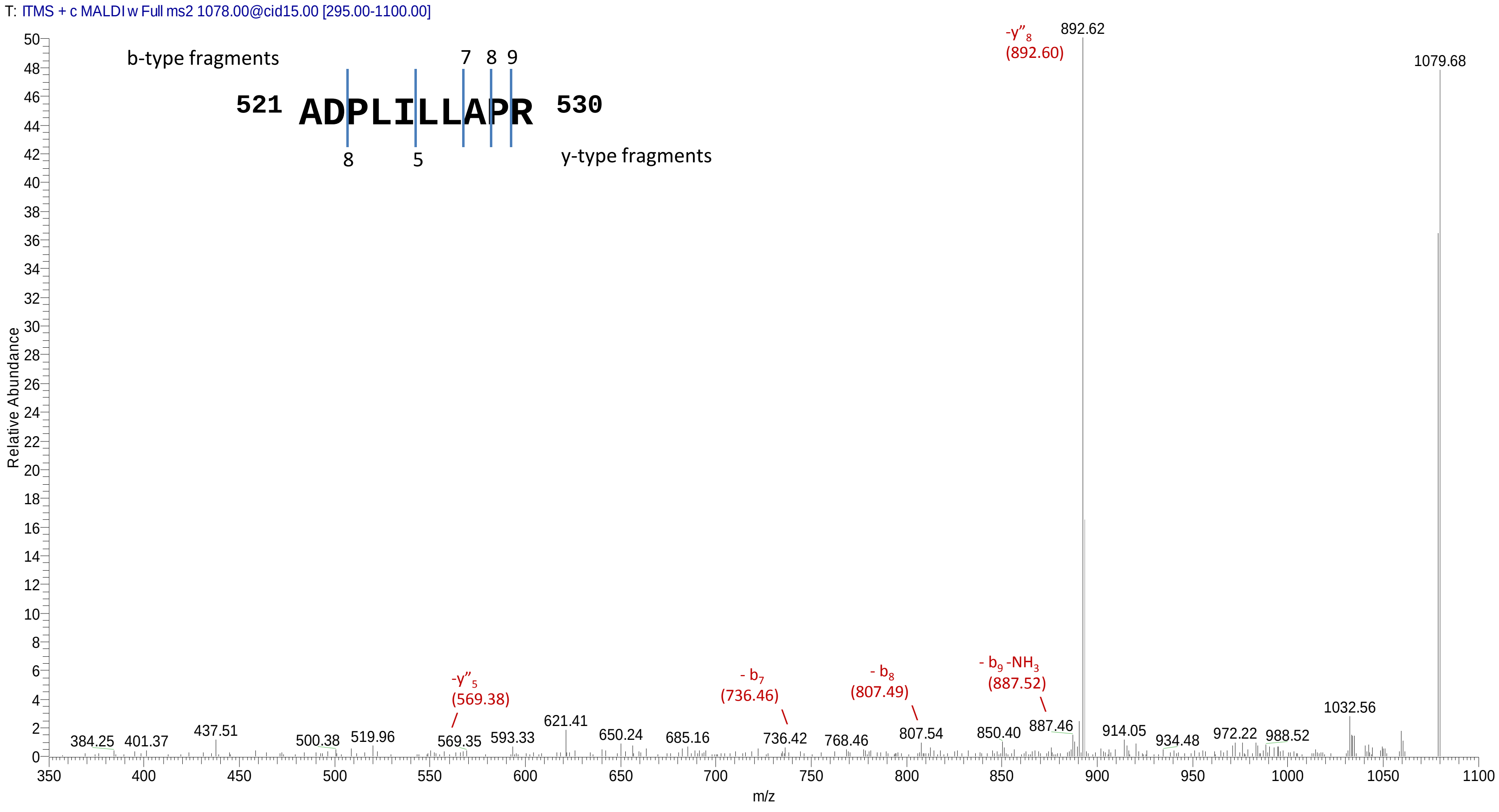

10. MS^2 m/z 1090

Theoretical mass: 1089.48 Da 679-687, *D. melanogaster*

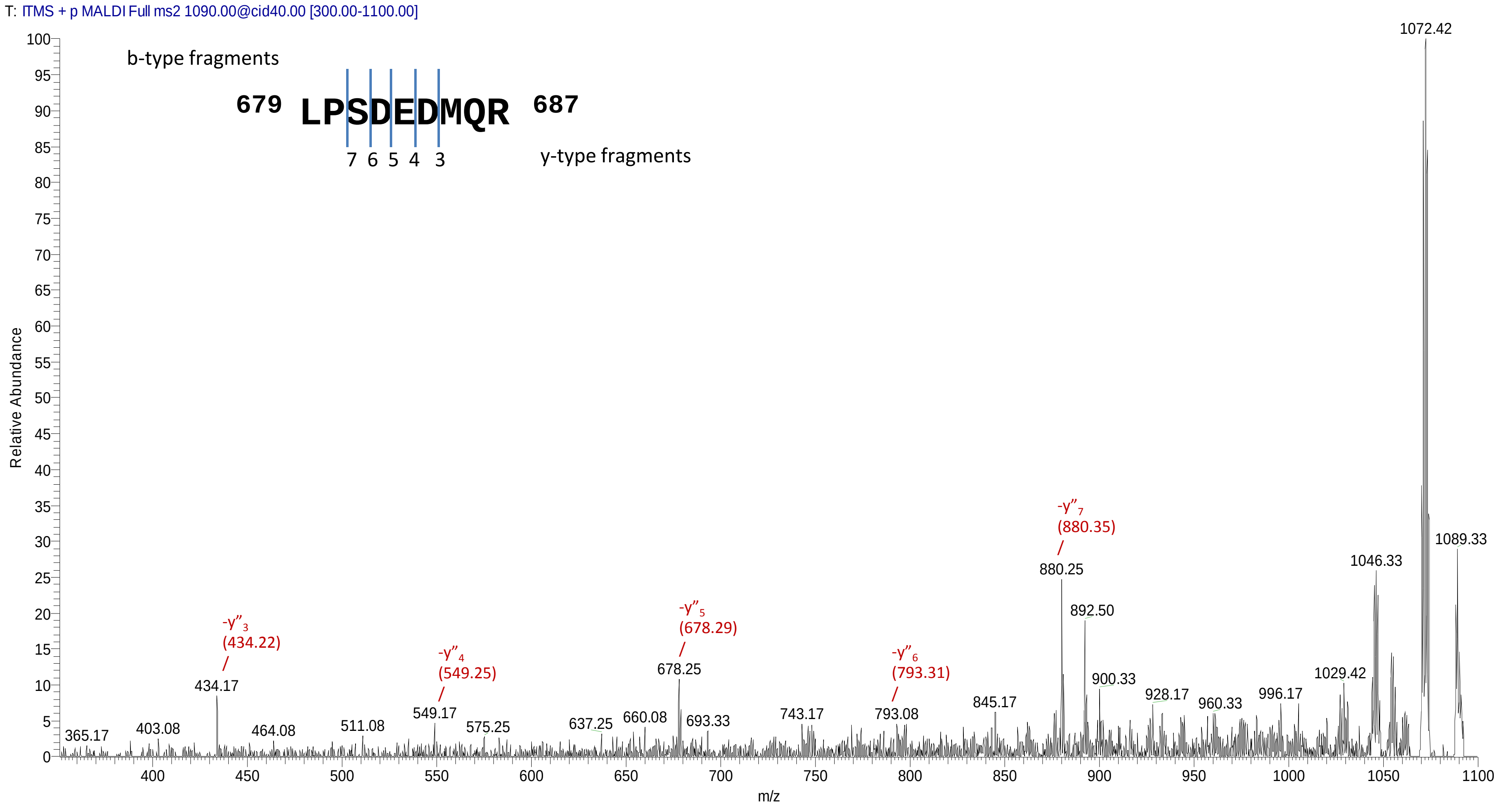

11. MS^2 m/z 1179

Theoretical mass: 1174.61 Da 17-25, *D. melanogaster*

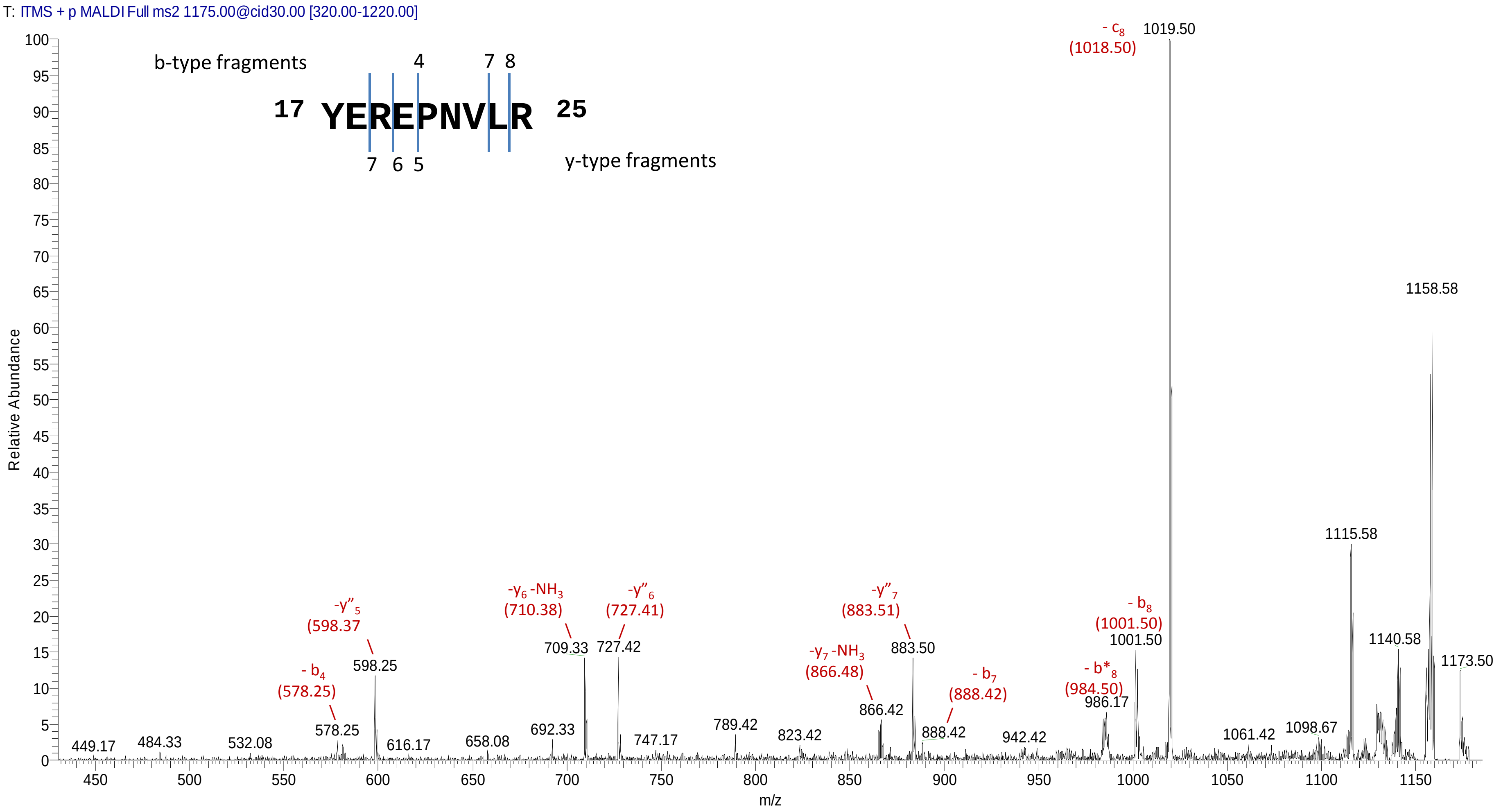

12. MS^2 m/z 1238

Theoretical mass: 1238.65 Da 663-672, *D. melanogaster*

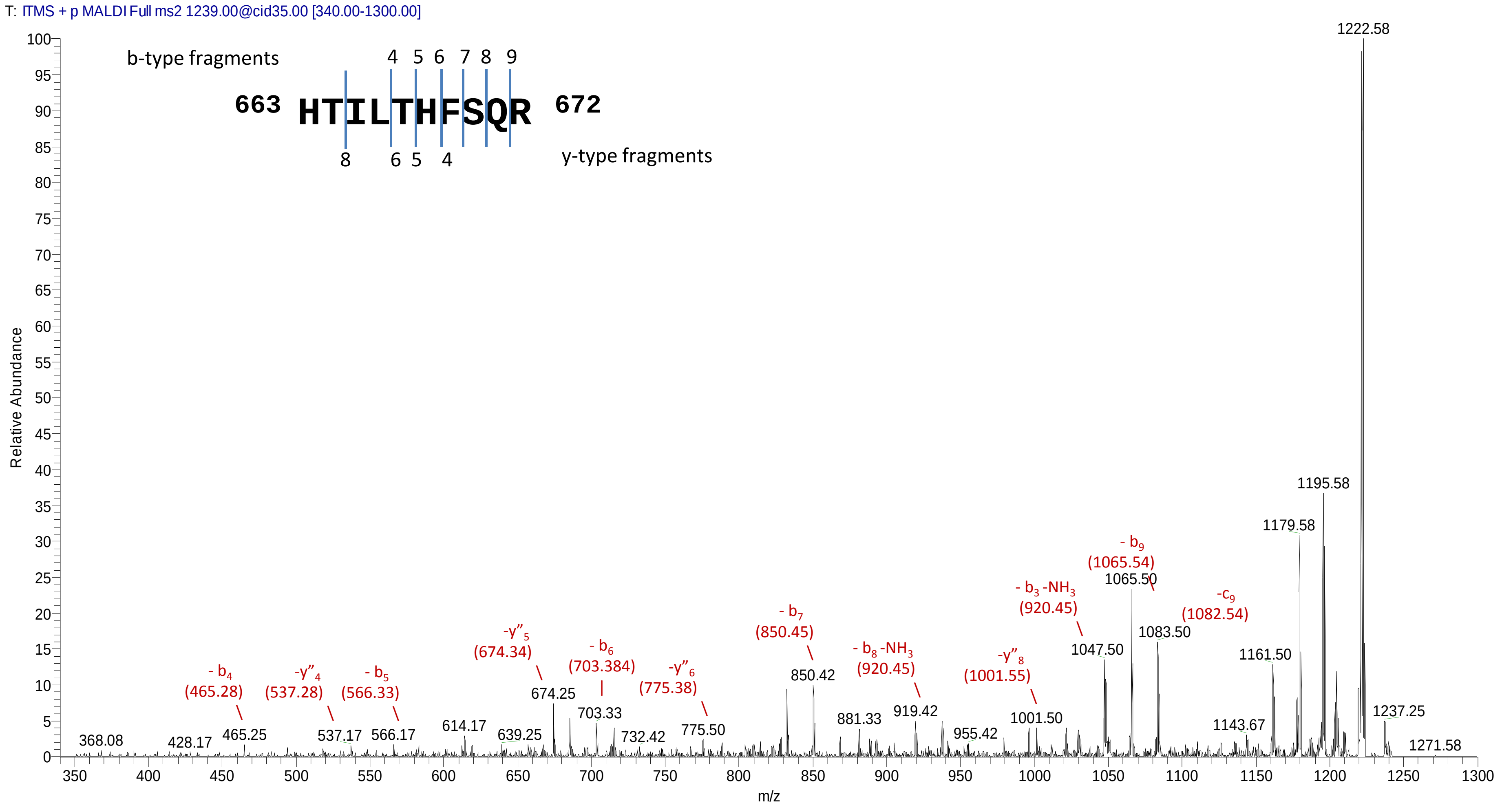

13. MS^2 m/z 1334

Theoretical mass: 1332.79 Da 571-582, *D. melanogaster*

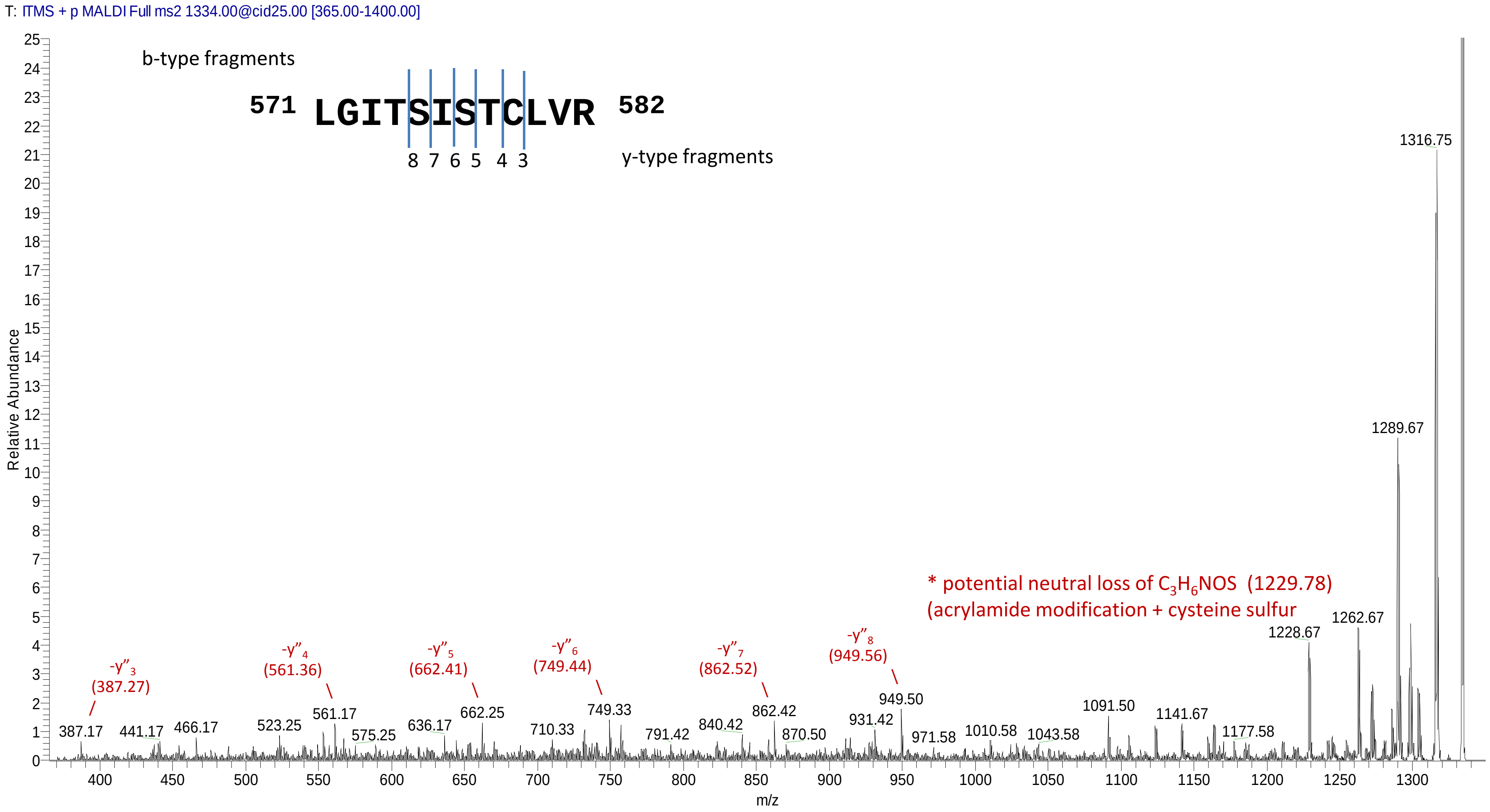

14. MS[2] m/z 1412

Theoretical mass: 1411.72 Da 645-657, *D. melanogaster*

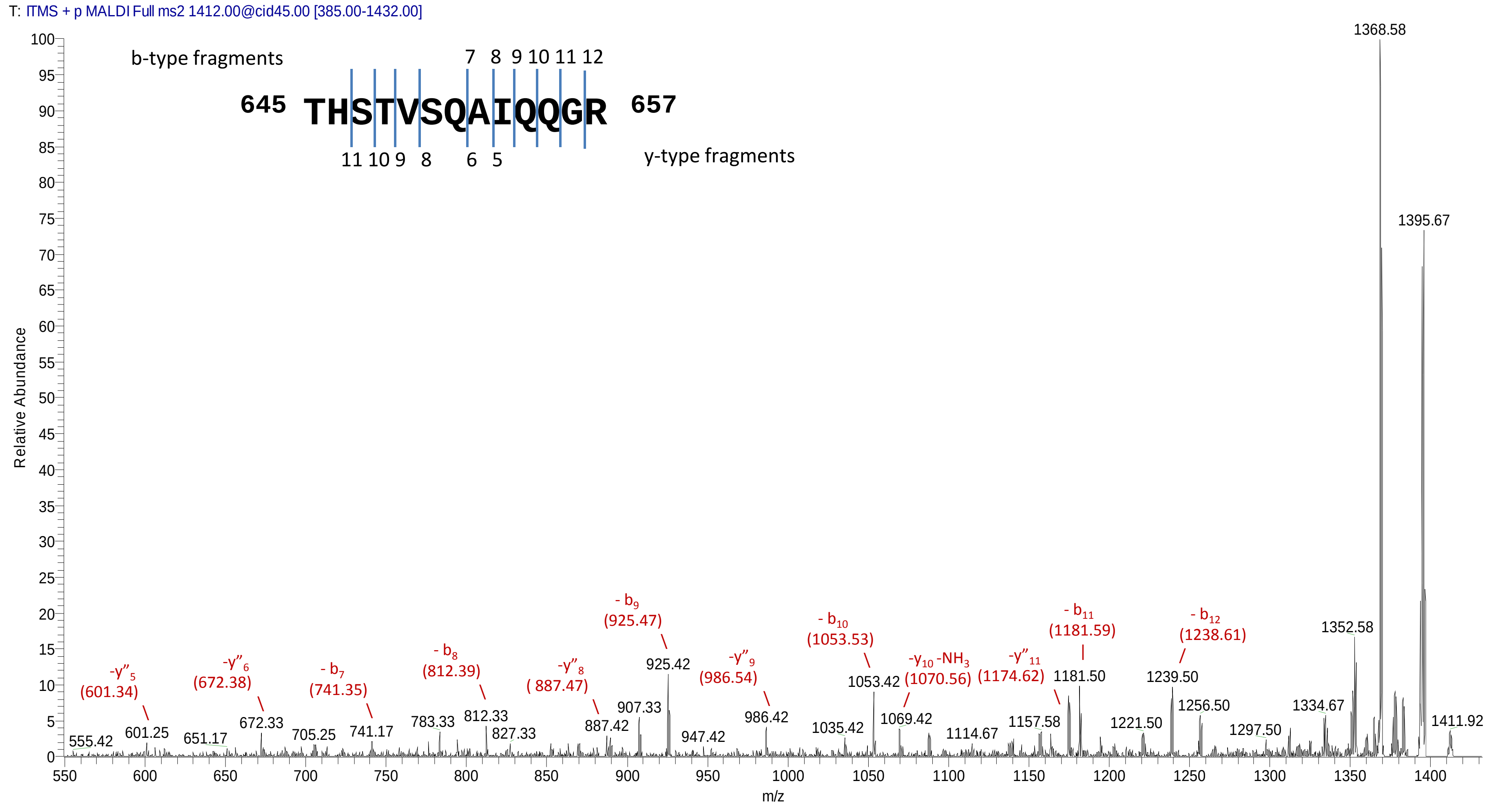

15. MS^2 m/z 1504

Theoretical mass: 1503.87 Da 172-183, *D. melanogaster*

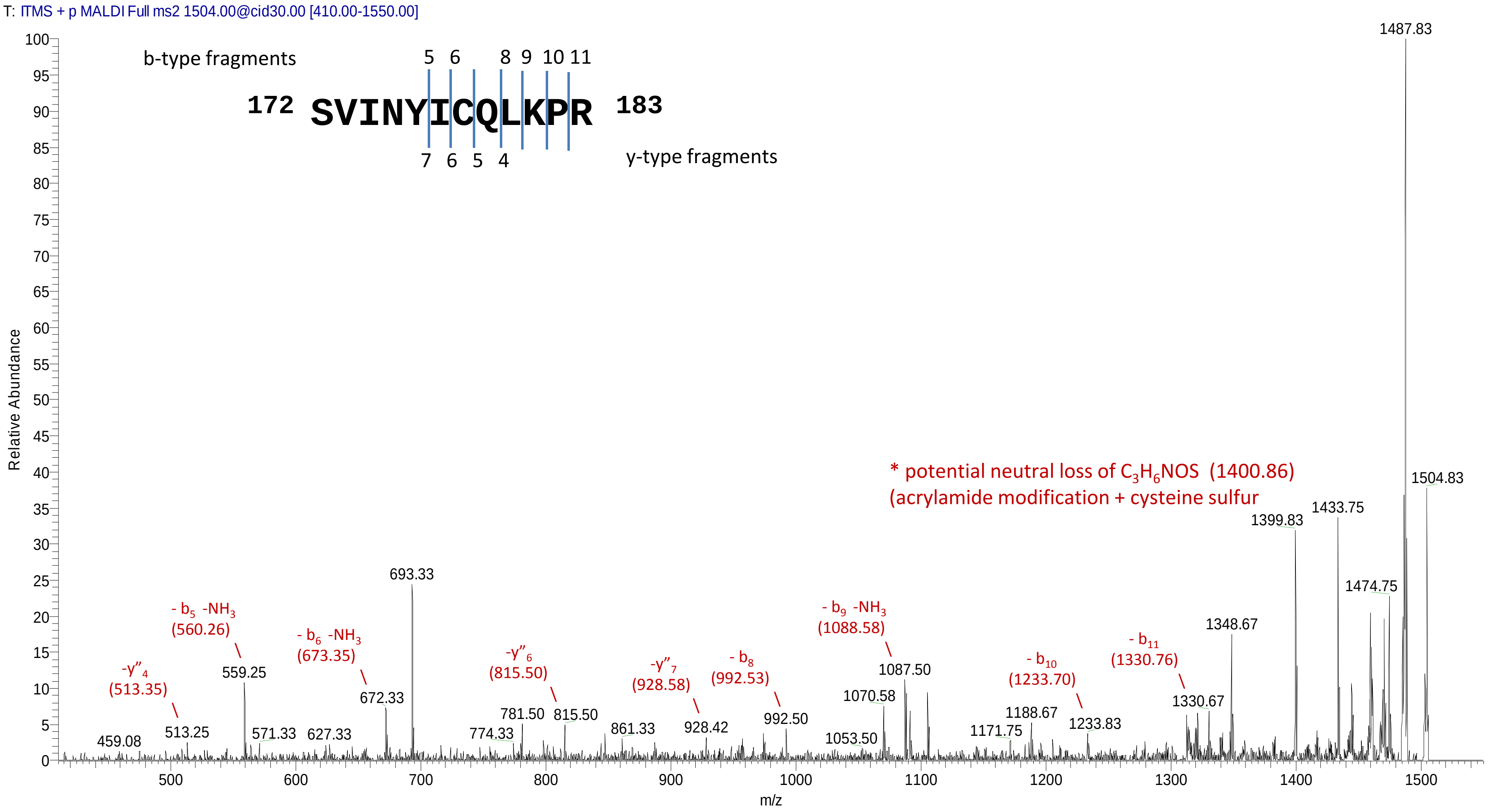

16. MS^2 m/z 1525

Theoretical mass: 1523.83 Da 403-420, *D. melanogaster*

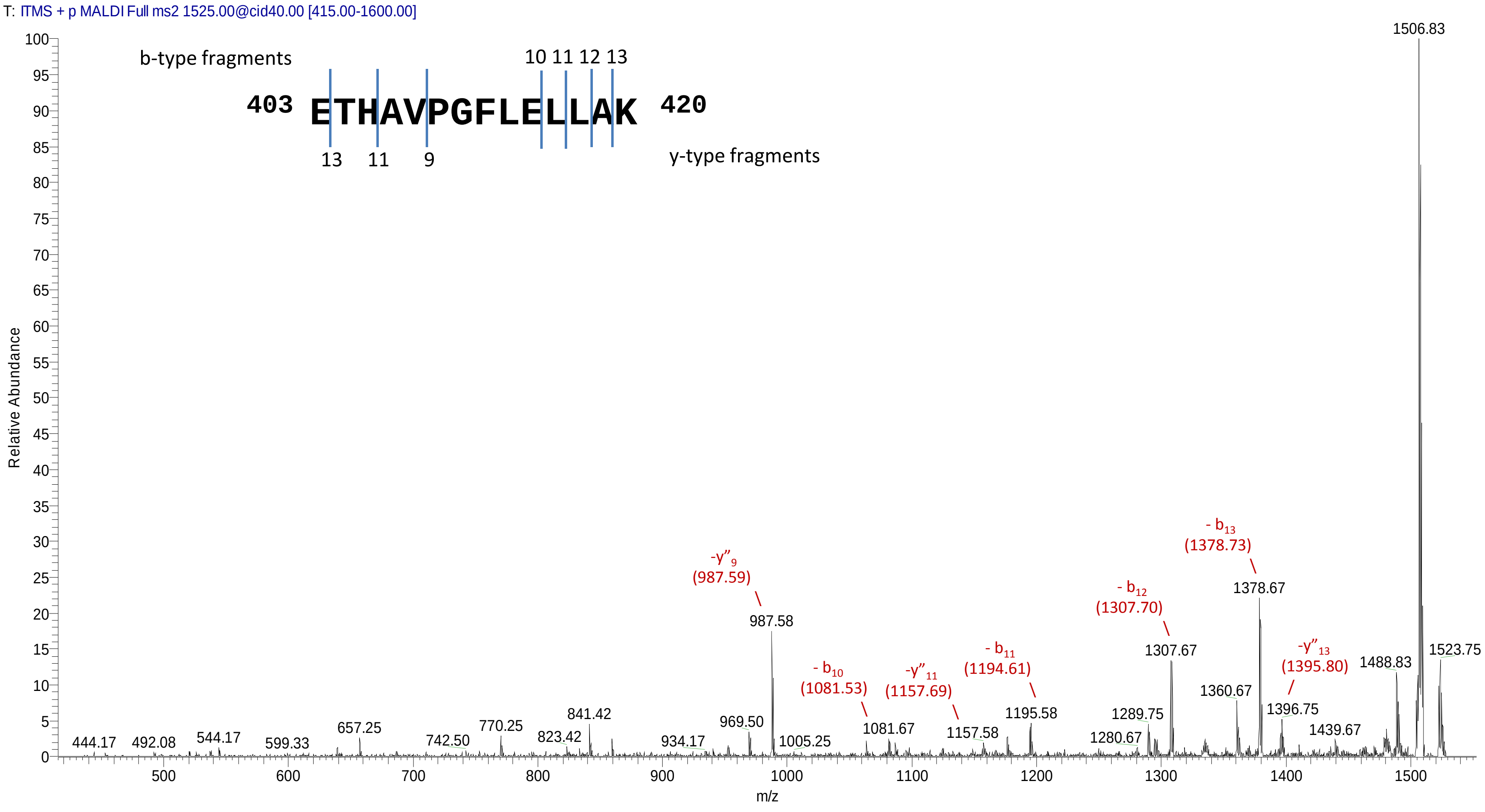

17. MS^2 m/z 1529

Theoretical mass: 1526.78 Da 158-171, *D. melanogaster*

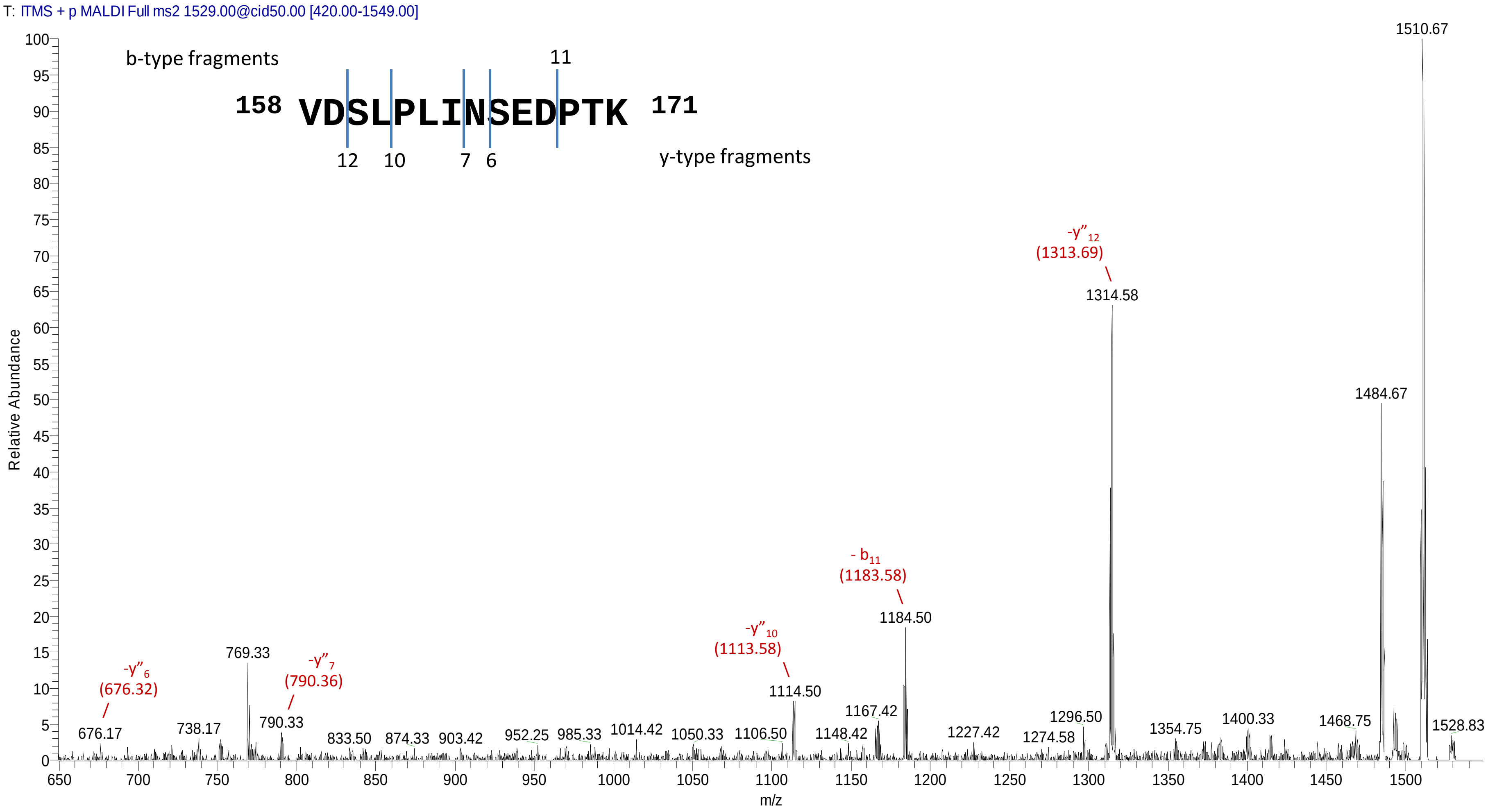

18. MS^2 m/z 1610

Theoretical mass: 1609.75 Da G_{-6} - K_9, *D. melanogaster*

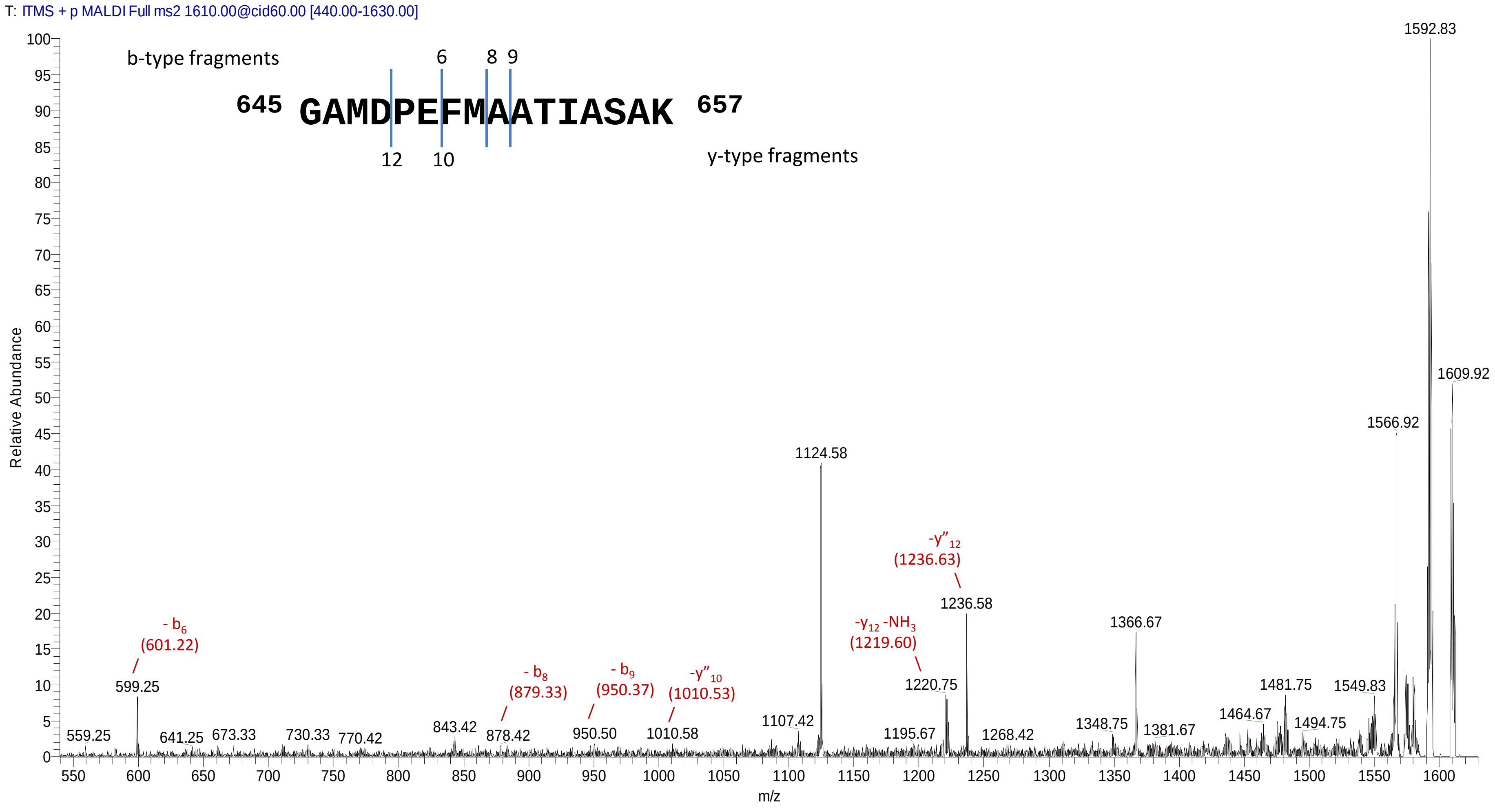

19. MS^2 m/z 1629

Theoretical mass: 1627.94 Da 731-743, *D. melanogaster*

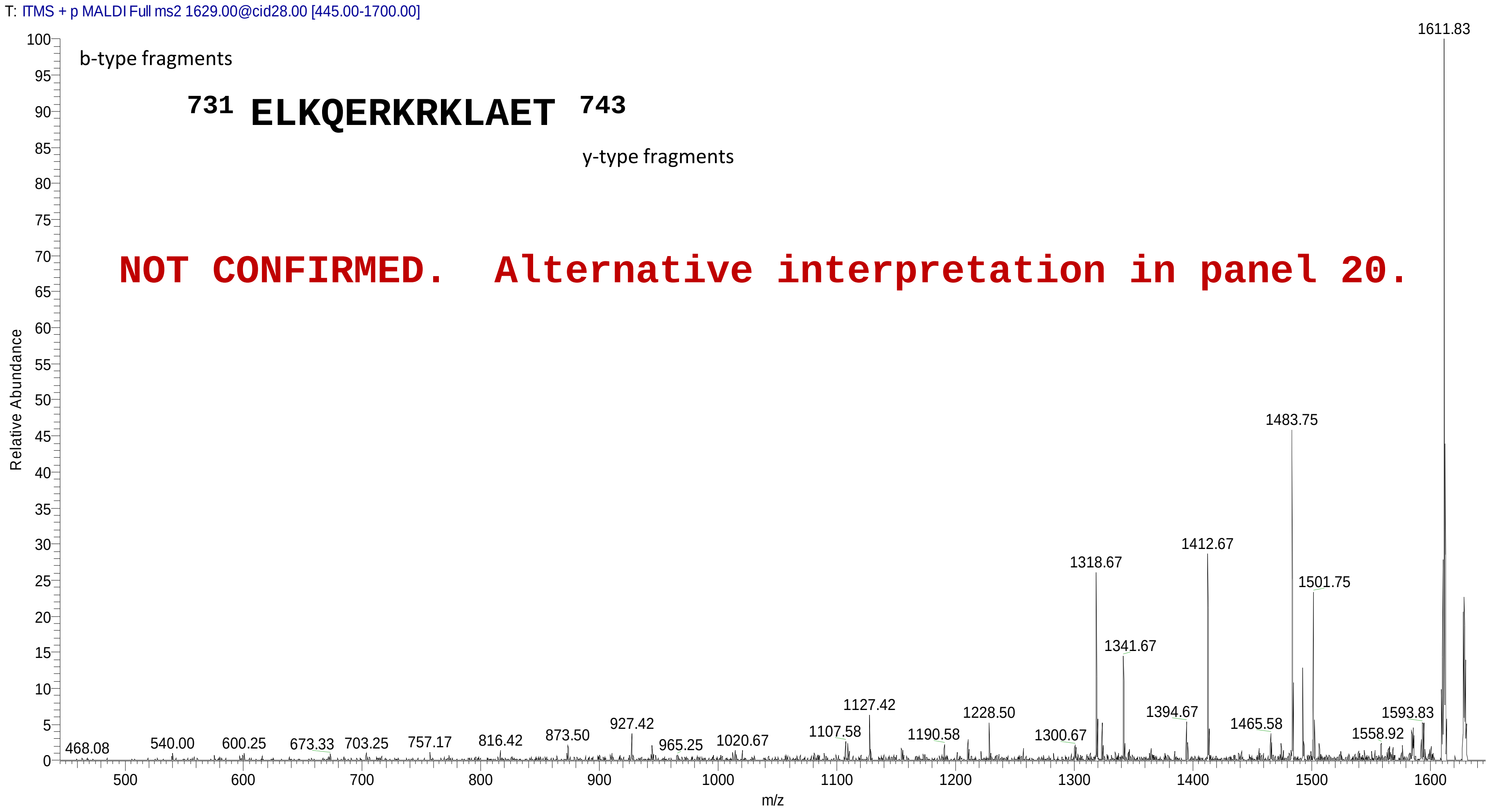

20. MS^2 m/z 1629

Theoretical mass: 1628.88 Da 583-597, *D. melanogaster*

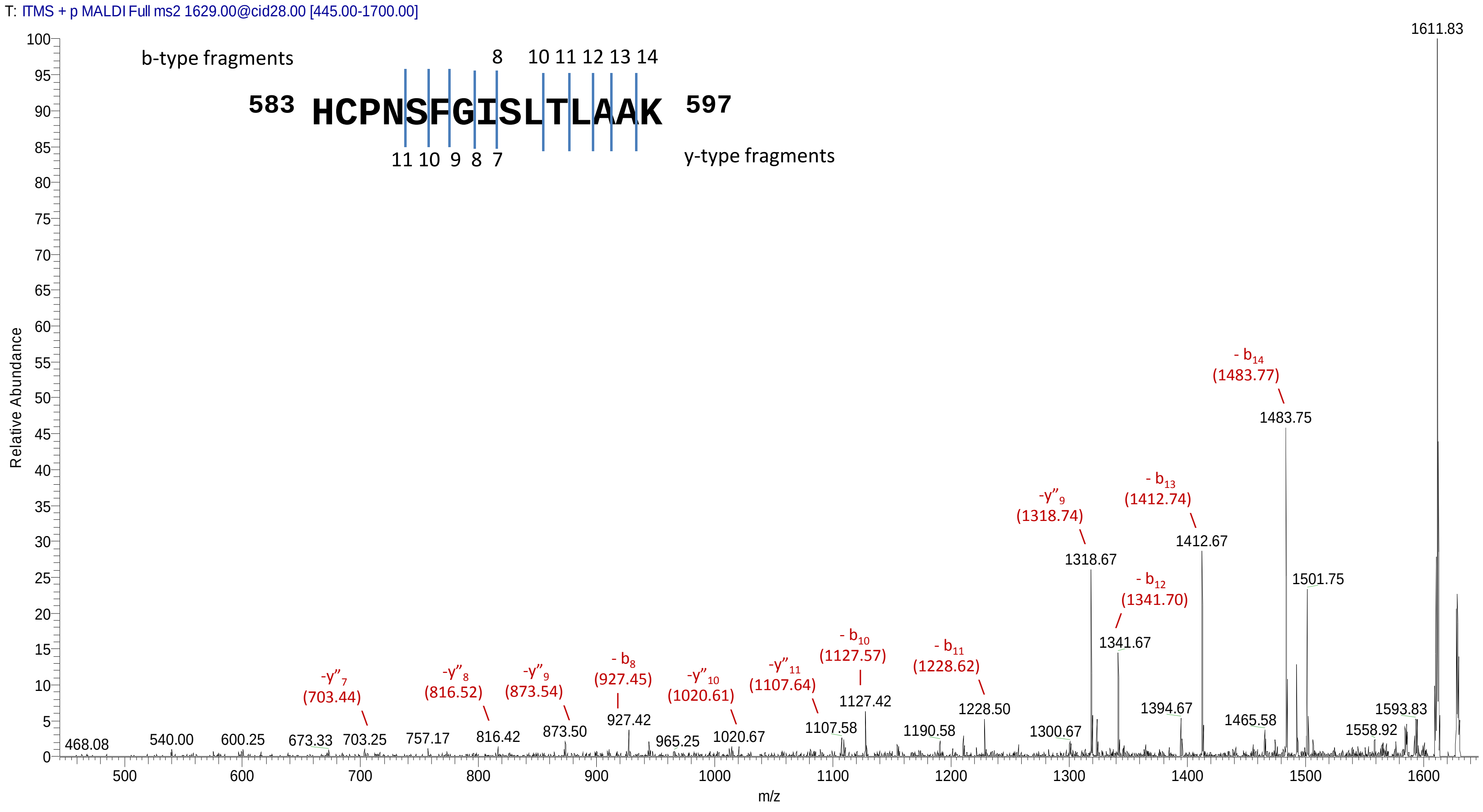

21. MS^2 m/z 1653

Theoretical mass: 1652.90 Da 643-657, *D. melanogaster*

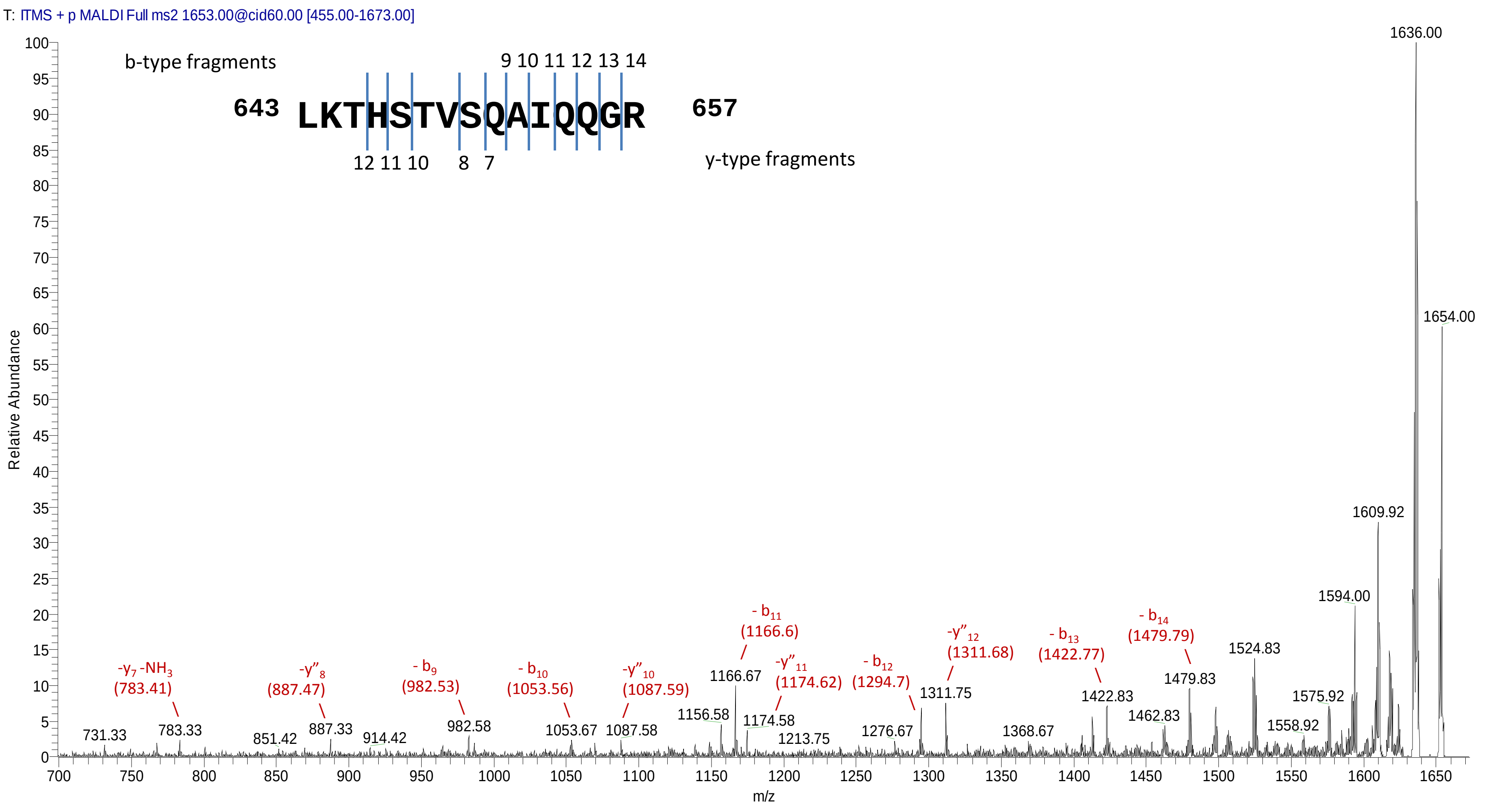

22. MS^2 m/z 1705

Theoretical mass: 1704.97 Da 192-207, *D. melanogaster*

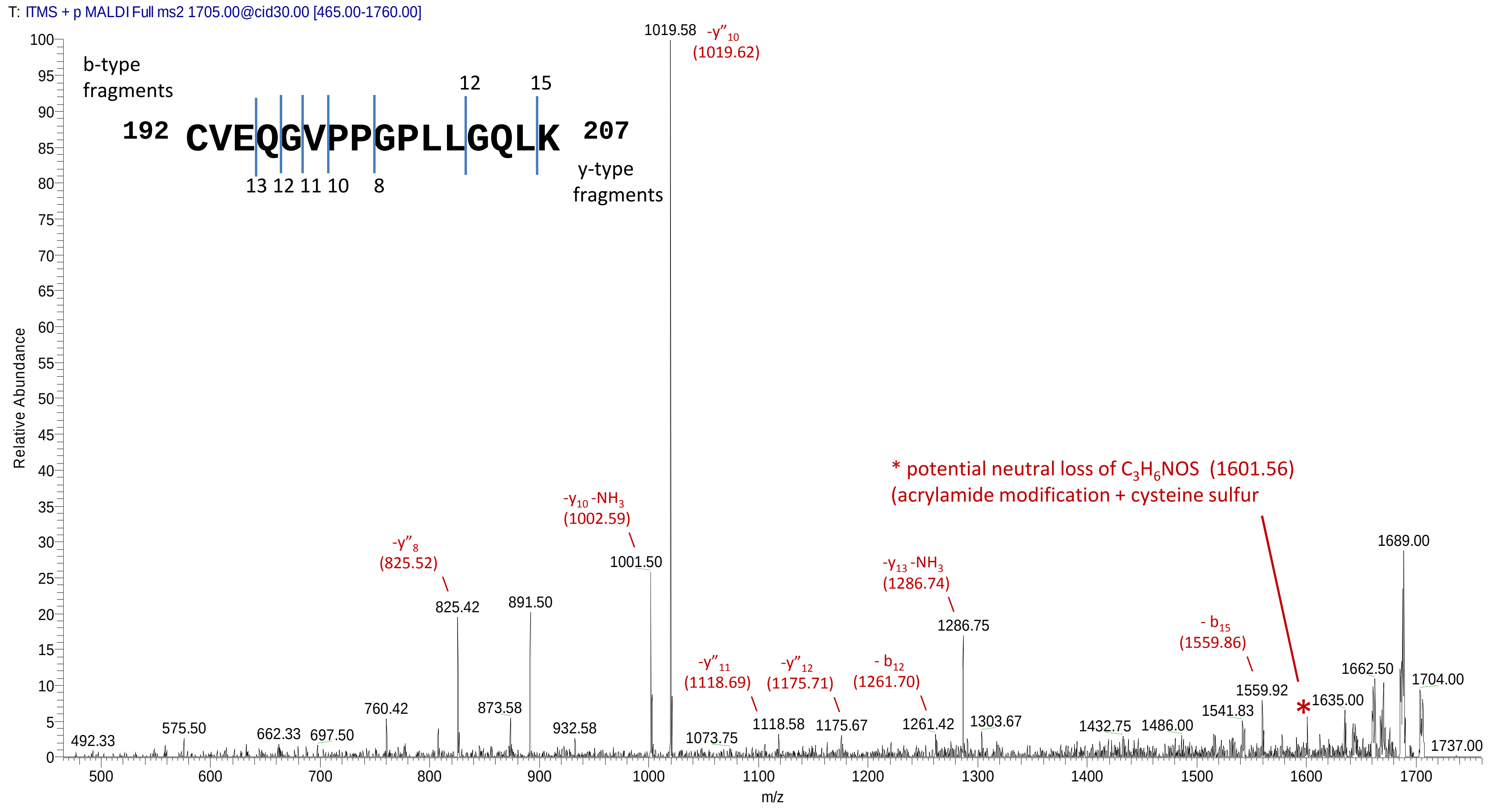

23. MS^2 m/z 1945

Theoretical mass: 1944.97 Da 113-130, *D. melanogaster*

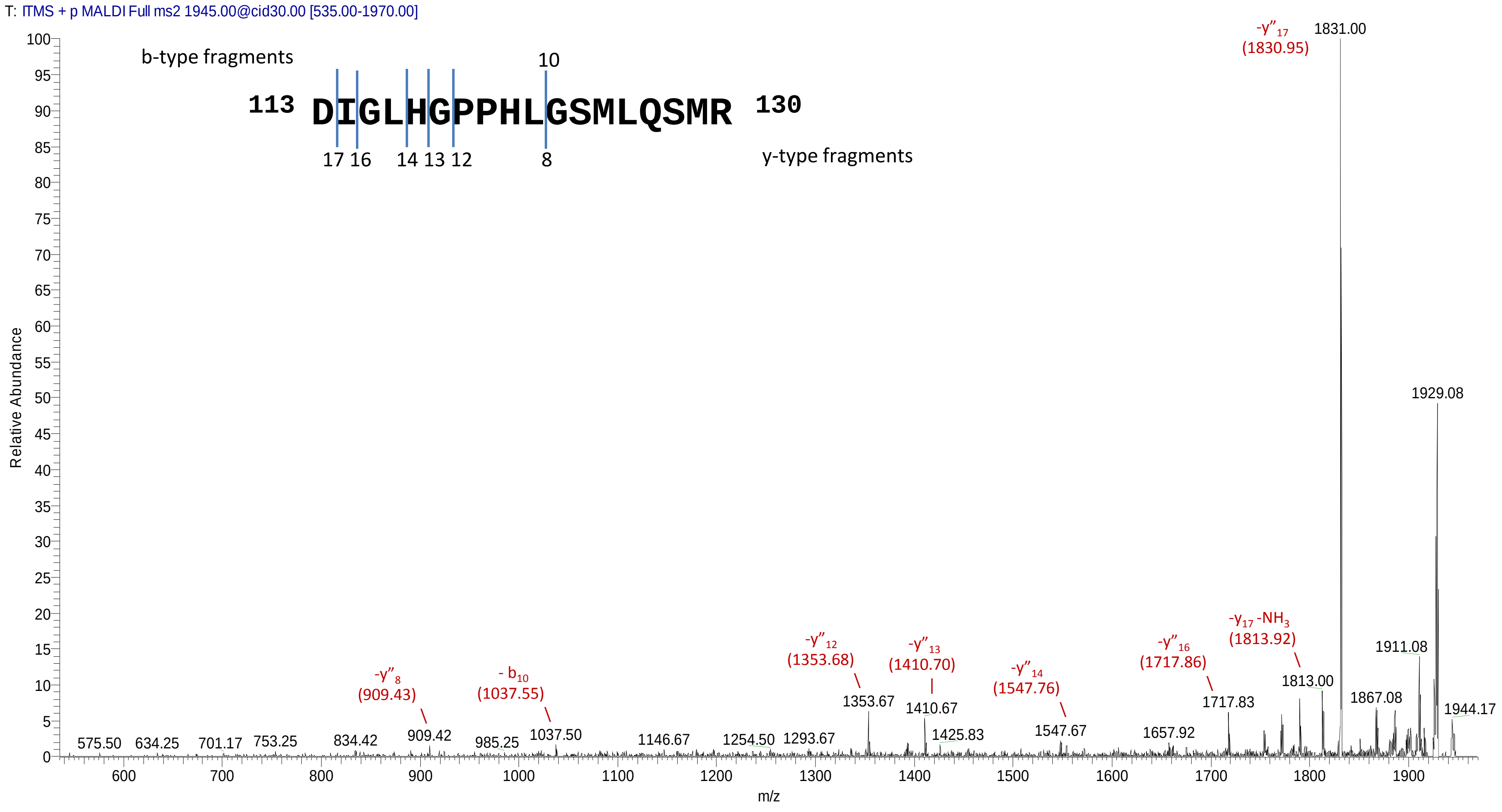

24. MS^2 m/z 2024

Theoretical mass: 2022.88 Da 417-433, *D. melanogaster*

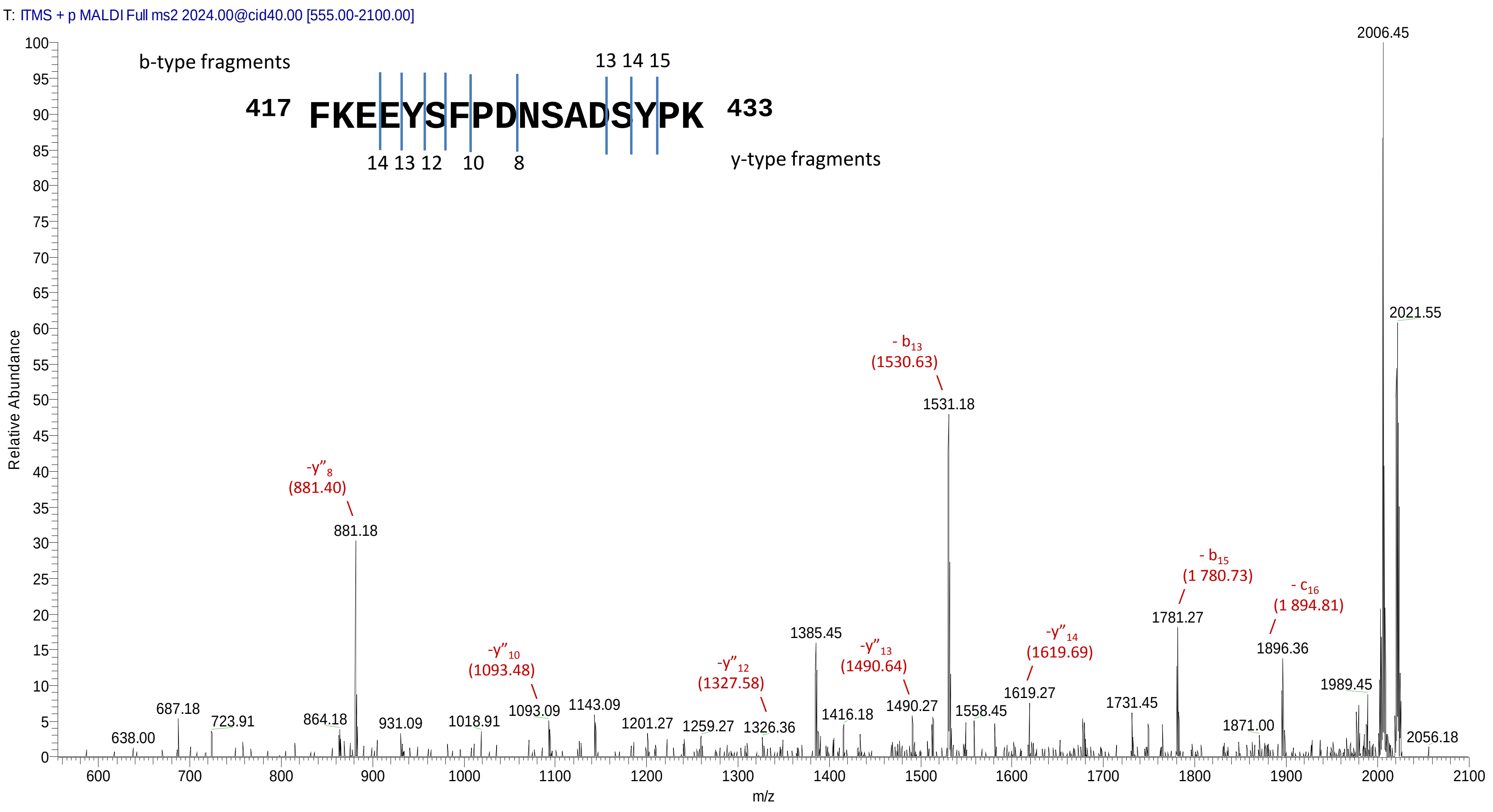

25. MS^2 m/z 2070

Theoretical mass: 2068.11 Da 264-281, *D. melanogaster*

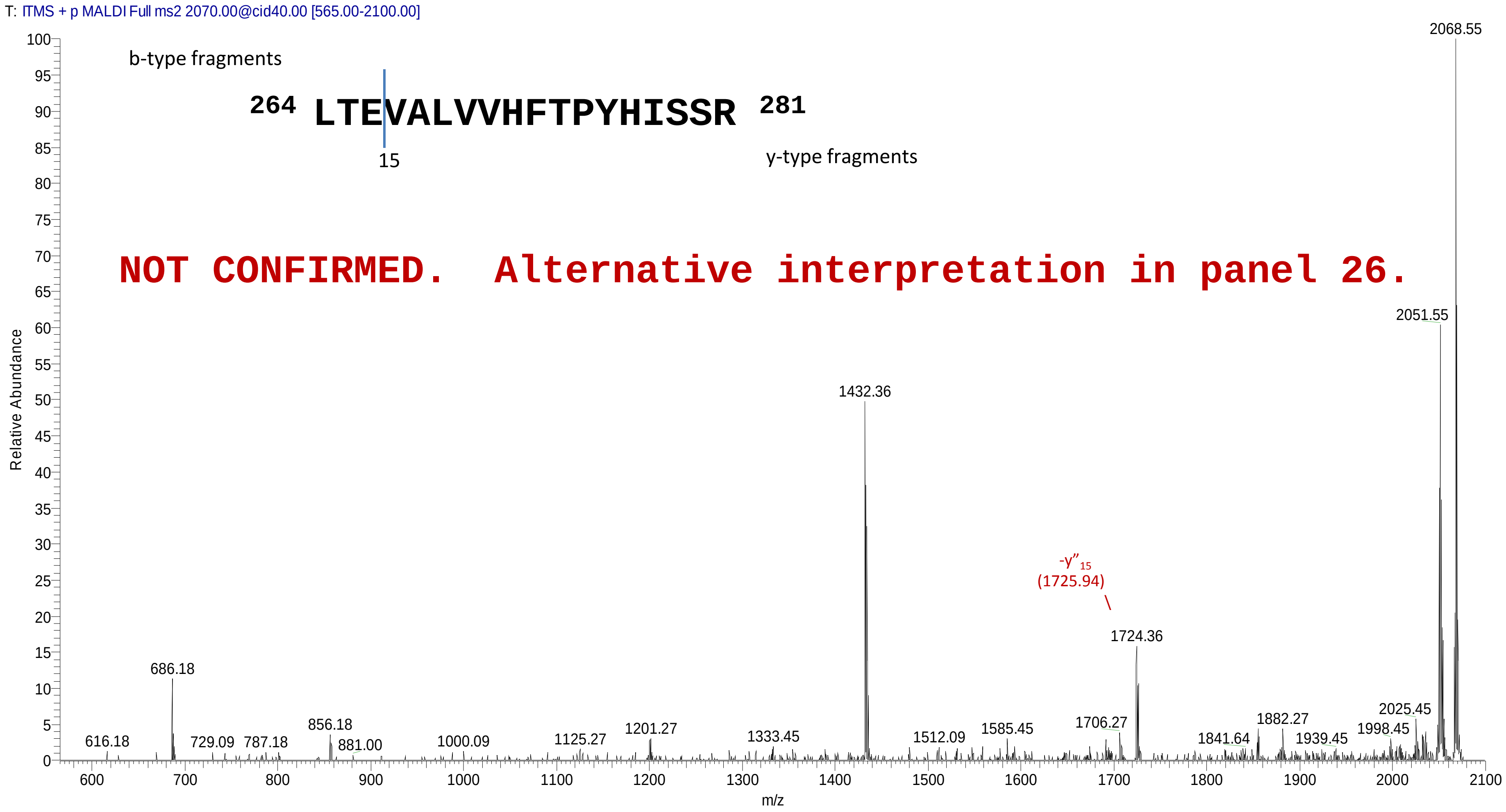

26. MS^2 m/z 2070

Theoretical mass: 2068.00 Da 605-622, *D. melanogaster*

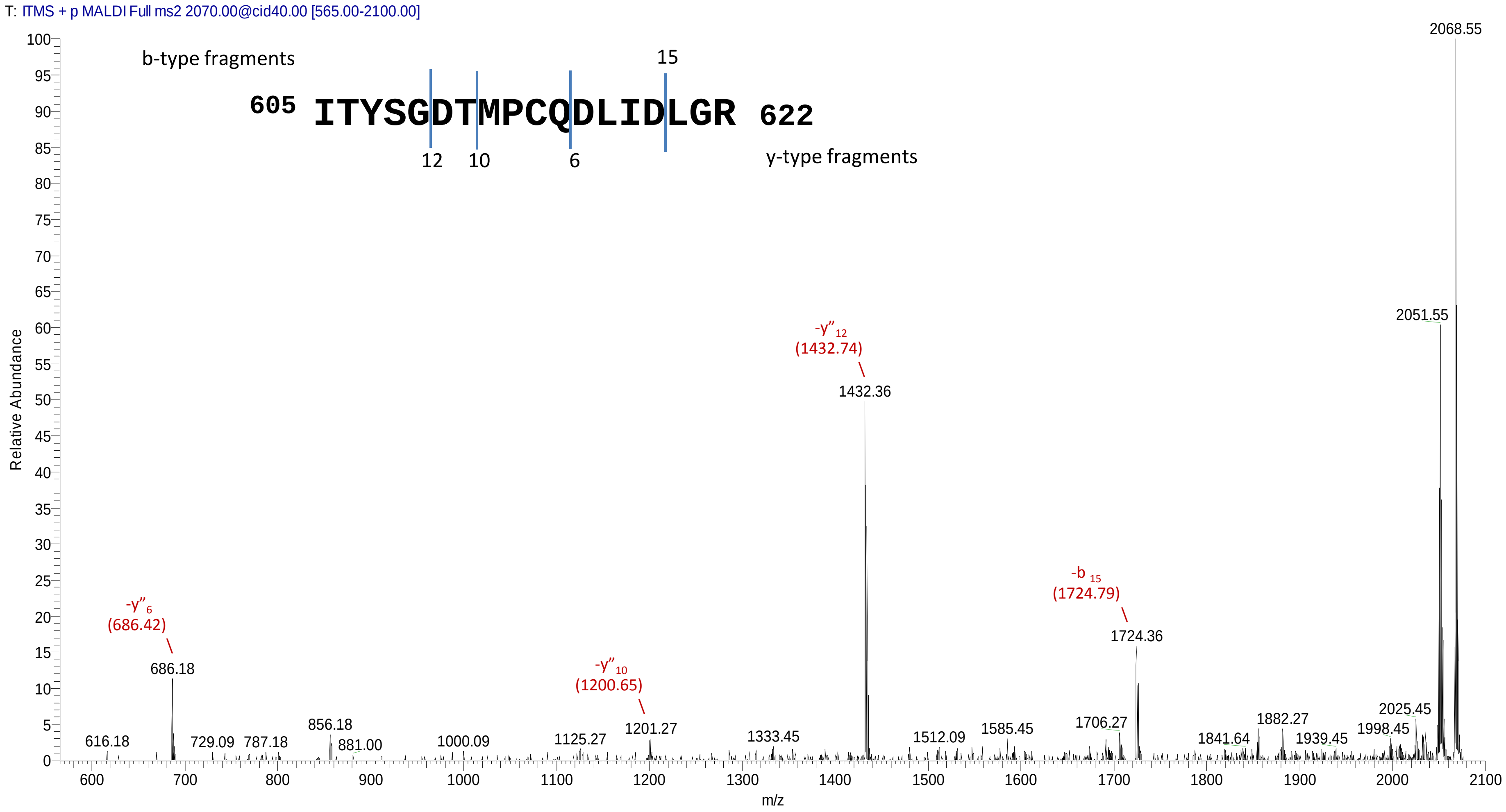

27. MS^2 m/z 2303

Theoretical mass: 2302.03 Da 623-642, *D. melanogaster*

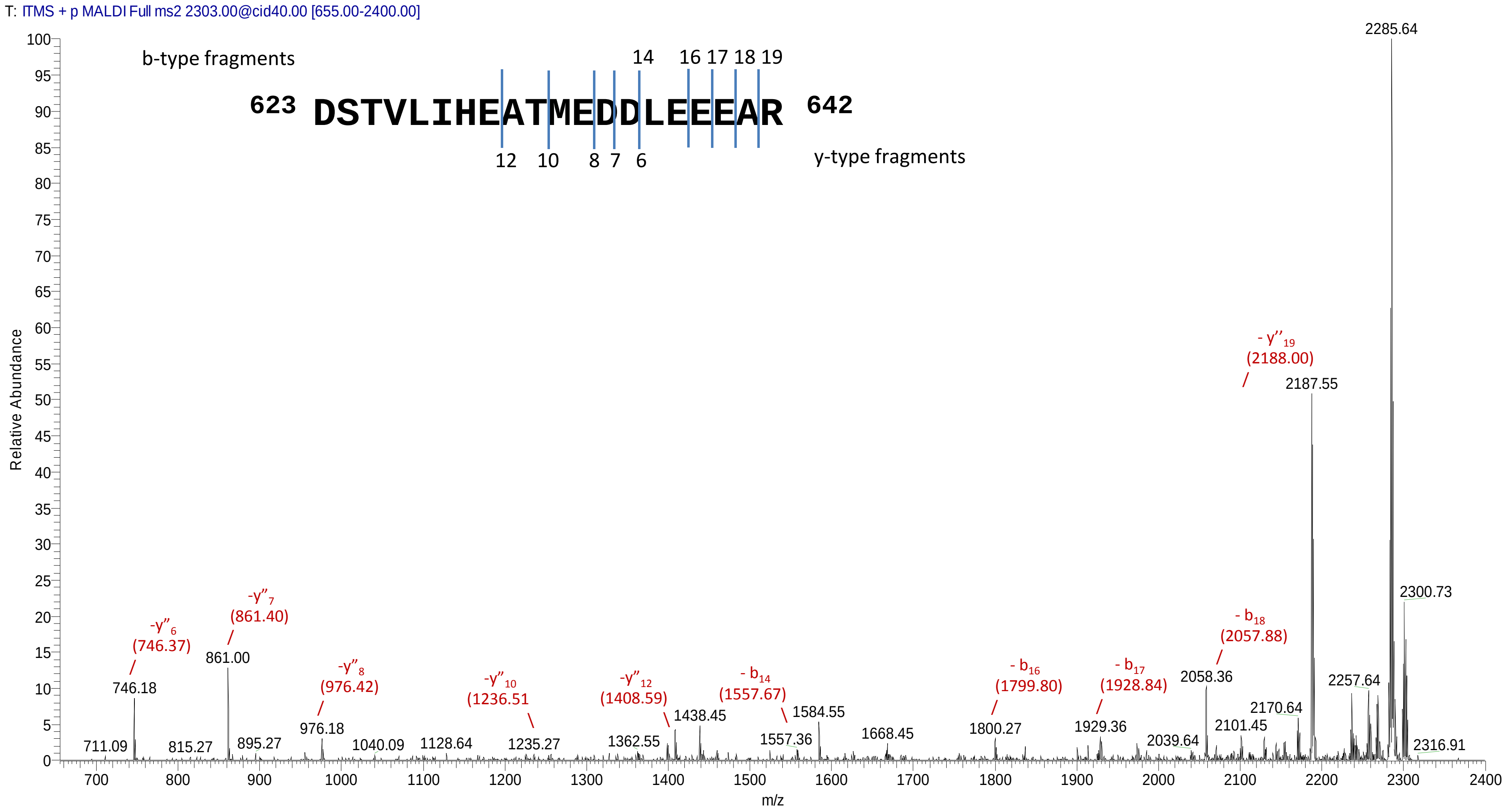

28. MS^2 m/z 2316

Theoretical mass: 2314.11 Da 365-384, *D. melanogaster*

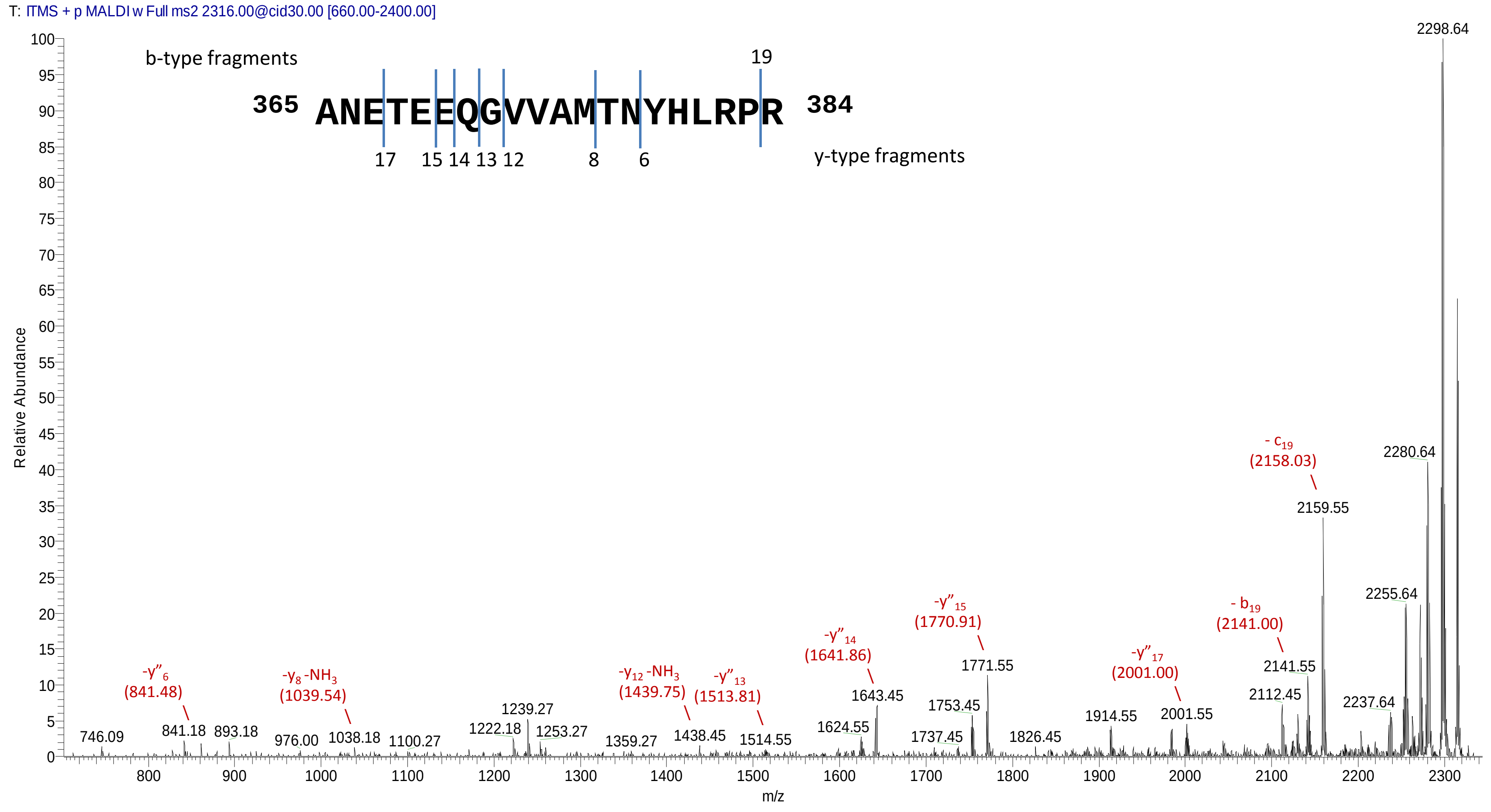

29. MS^2 m/z 2329

Theoretical mass: 2327.19 Da 457-477, *D. melanogaster*

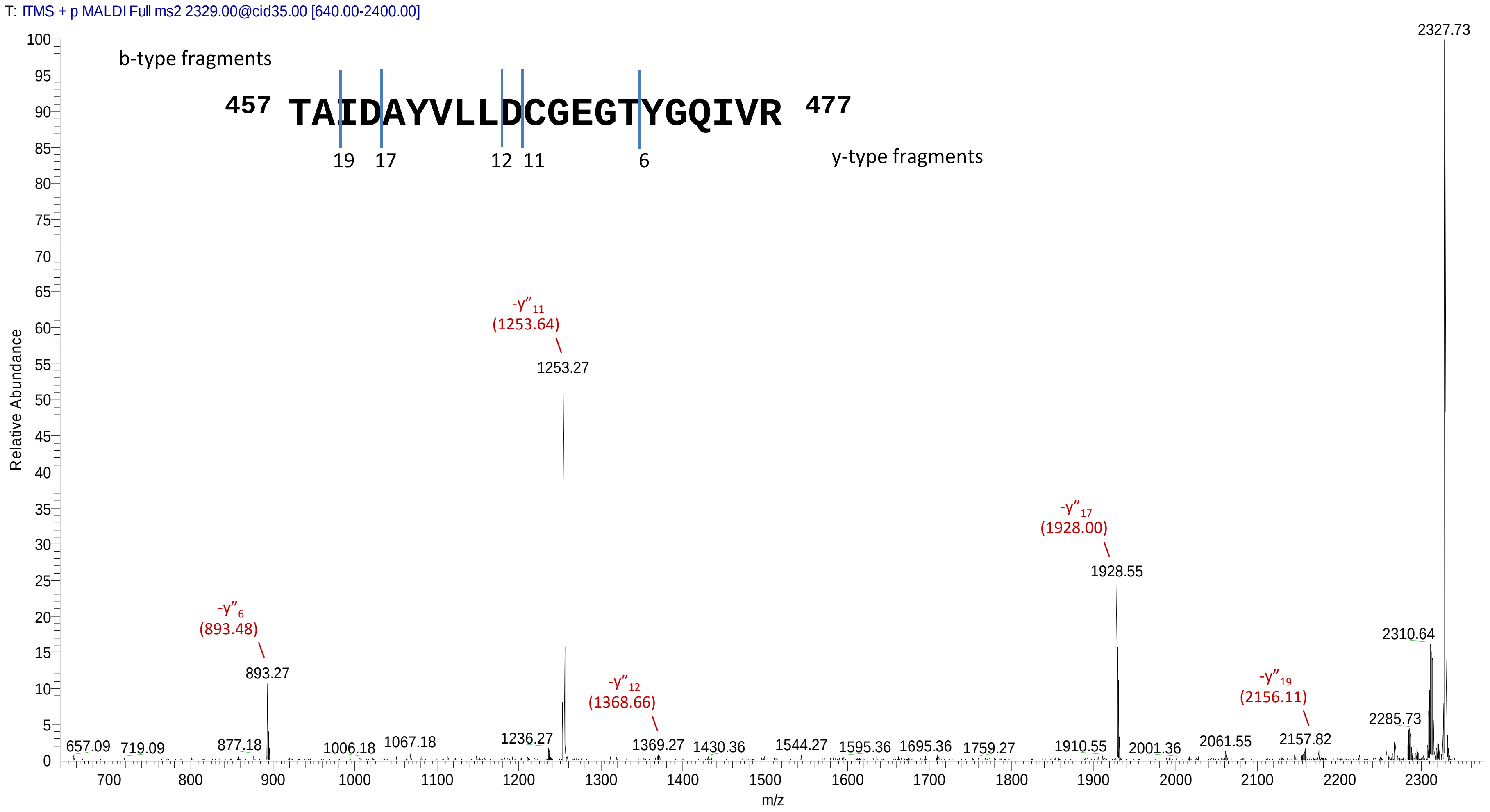

30. MS^2 m/z 2338

Theoretical mass: 2336.10 Da   708-726, *D. melanogaster*

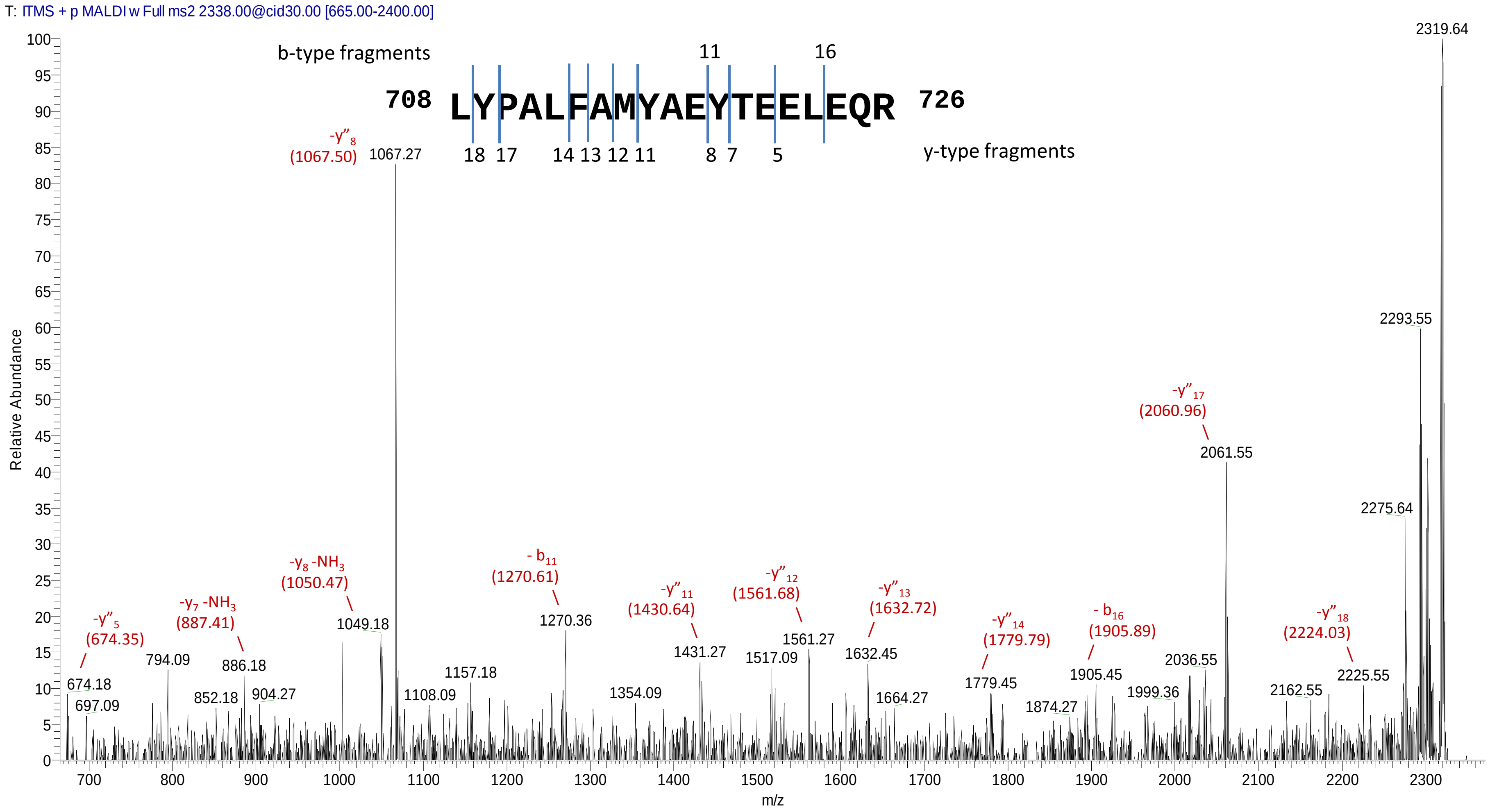

31. MS^2 m/z 2373

Theoretical mass: 2372.15 Da 688-707, *D. melanogaster*

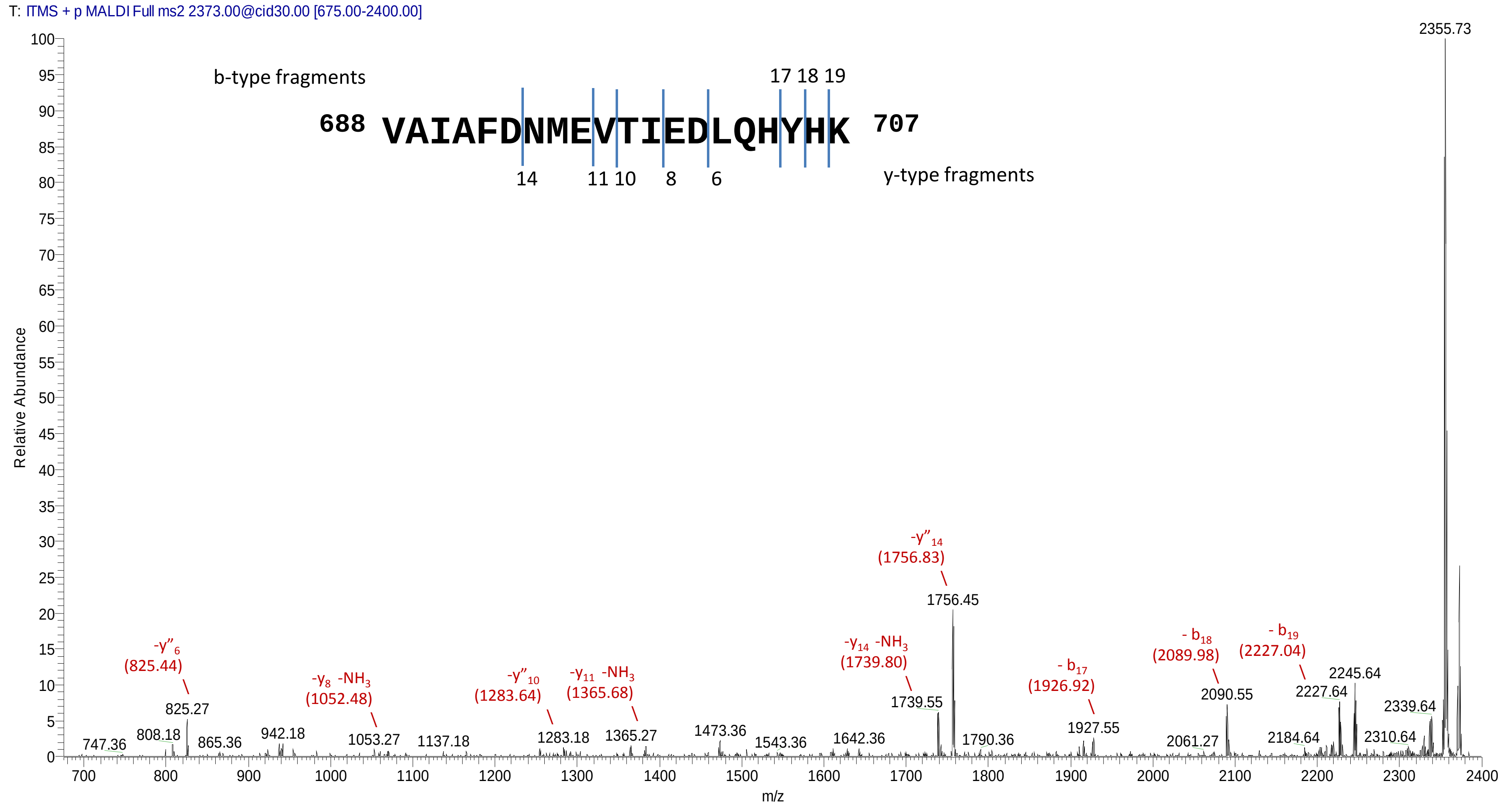

32. MS^2 m/z 2481

Theoretical mass: 2479.23 Da 137-157, *D. melanogaster*

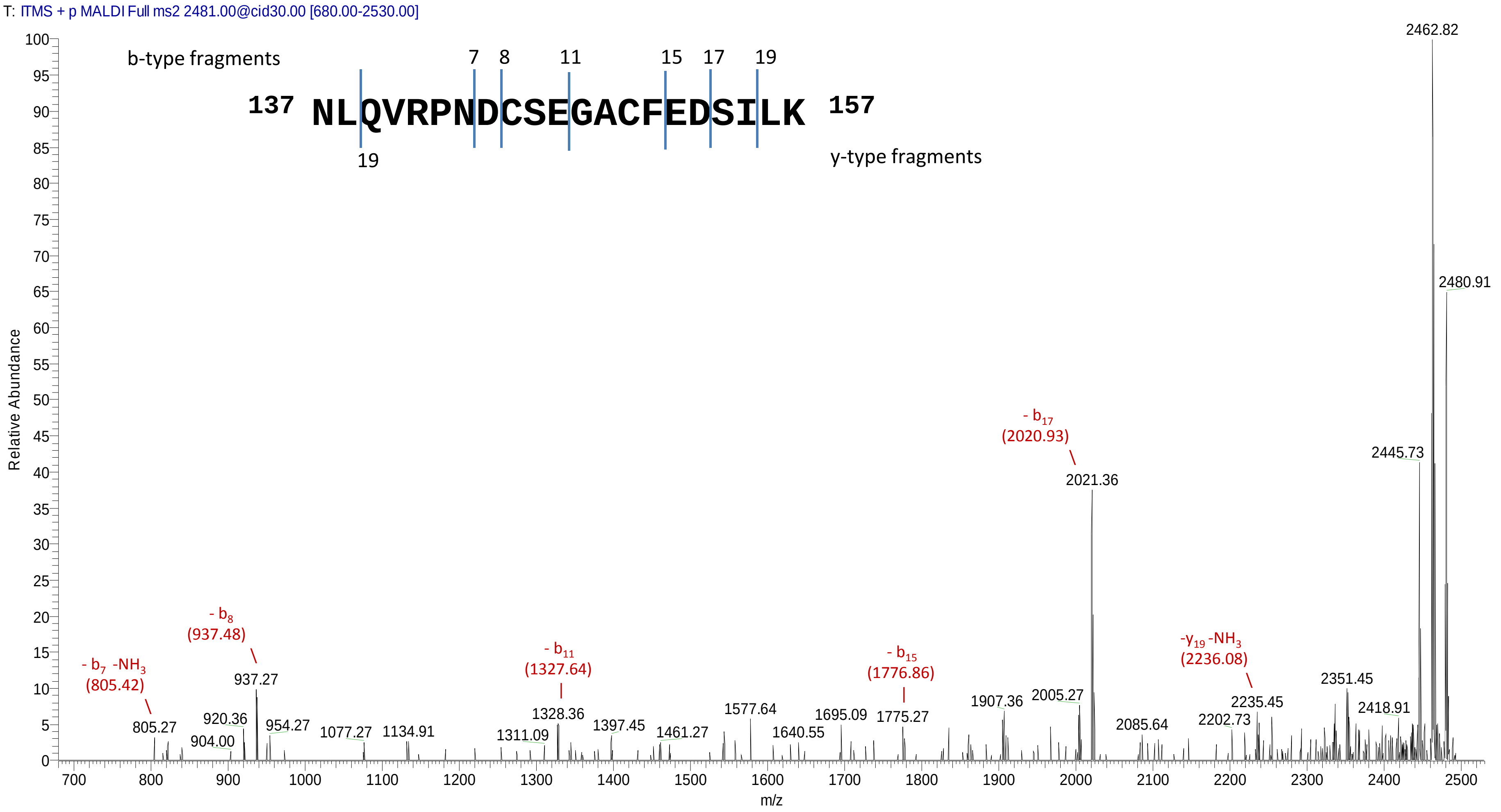

33. MS^2 m/z 2626

Theoretical mass: 2624.40 Da 259-281, *D. melanogaster*

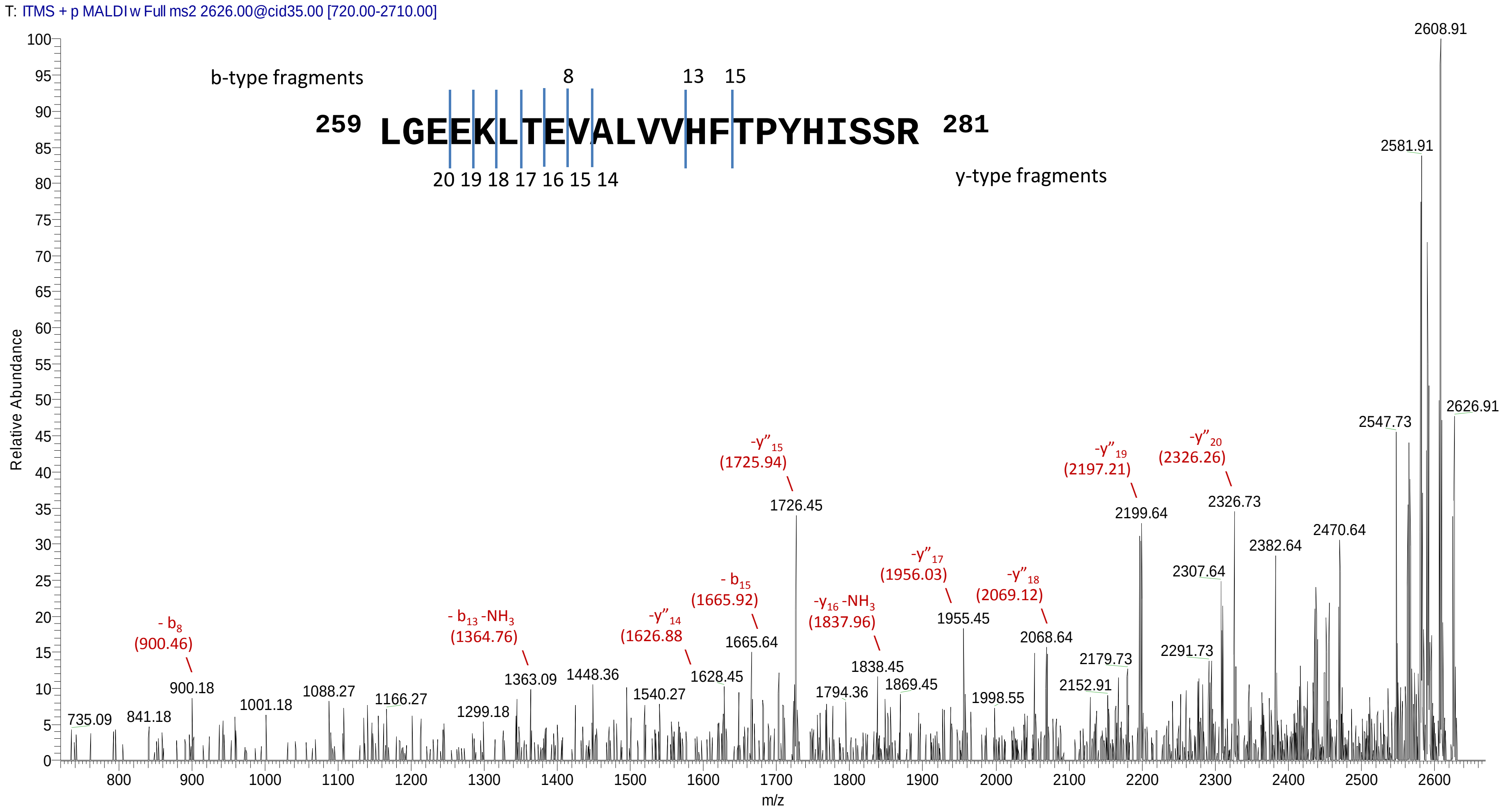

34. MS^2 m/z 3101

Theoretical mass: 3099.56 Da 663-687, *D. melanogaster*

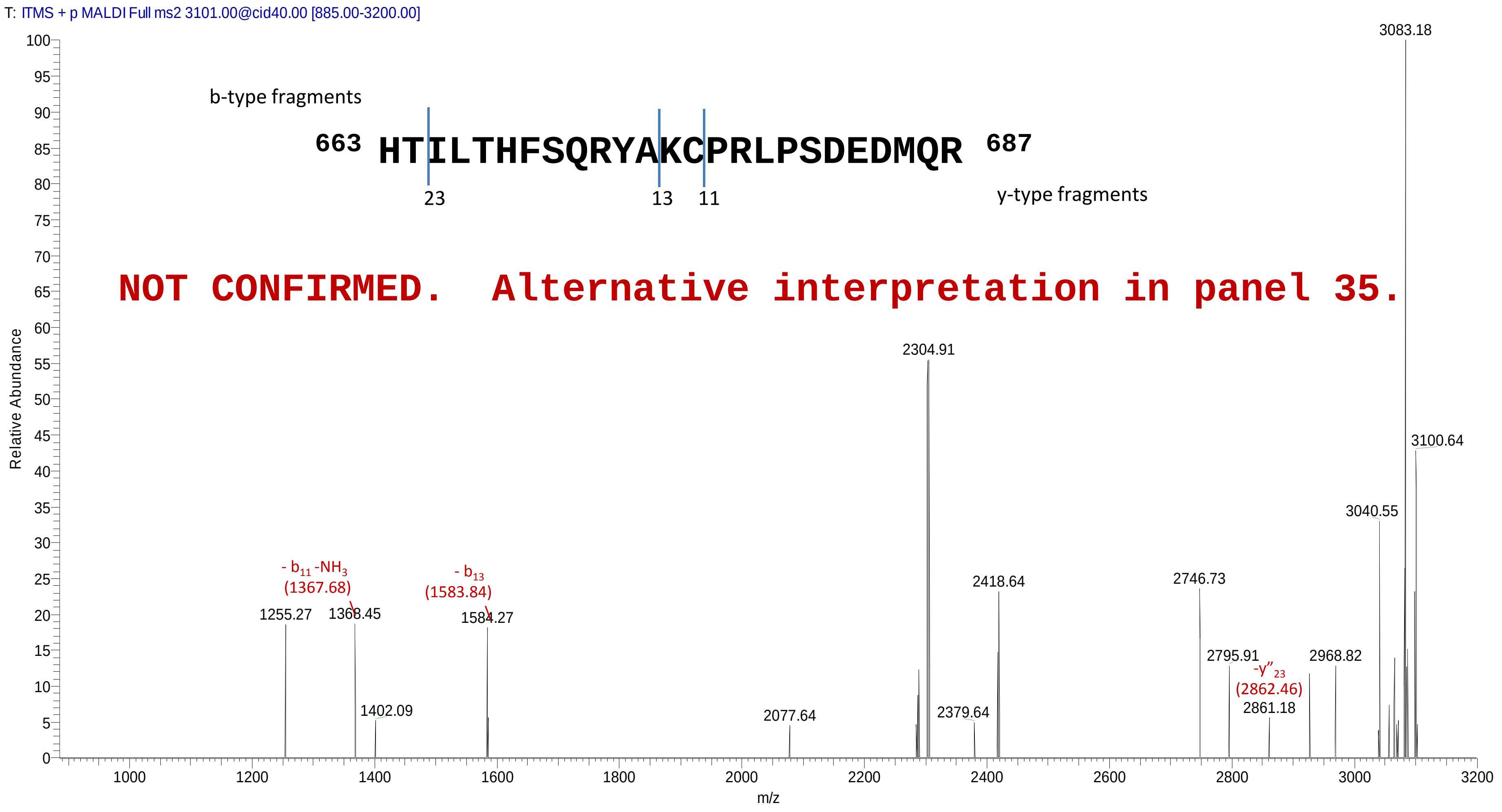

35. MS^2 m/z 3101

Theoretical mass: 3100.55 Da 542-570, *D. melanogaster*

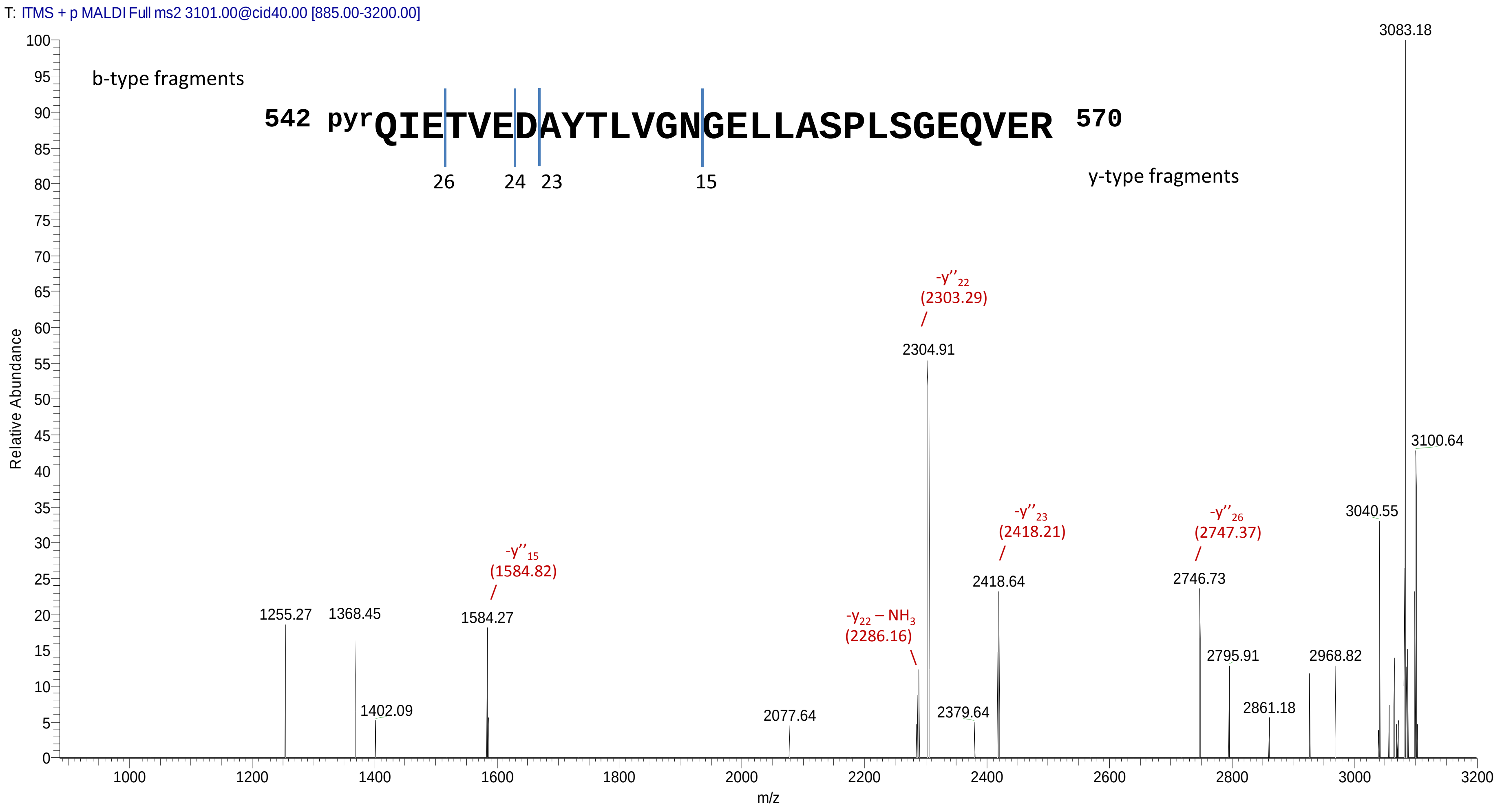

36. MS^2 m/z 3118

Theoretical mass: 3116.55 Da 542-570, *D. melanogaster*

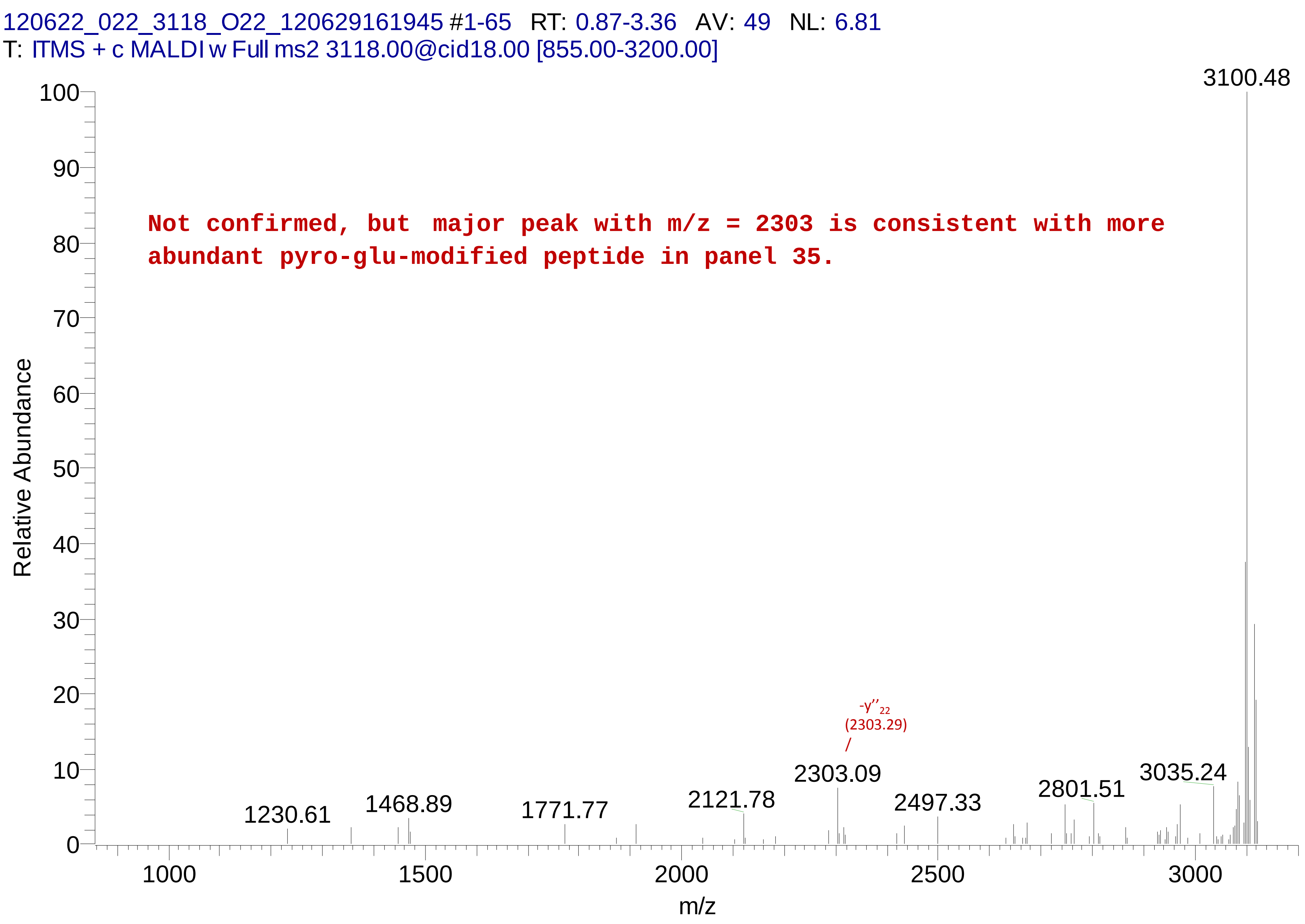

37. MS^2 m/z 3258

Theoretical mass: 3255.72 Da 28-60, *D. melanogaster*

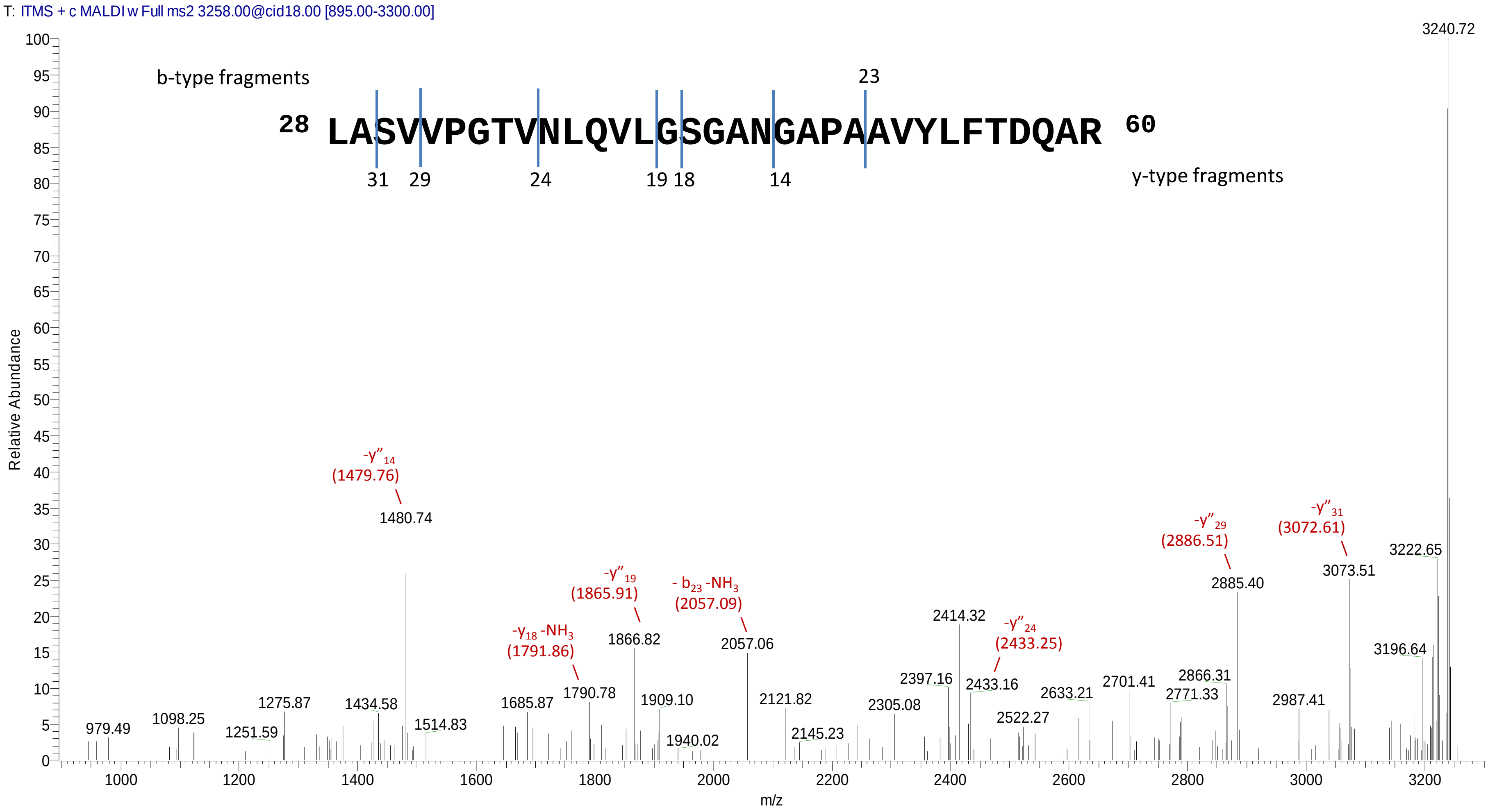

38. MS^2 m/z 3274

Theoretical mass: 3272.73 Da 83-112, *D. melanogaster*

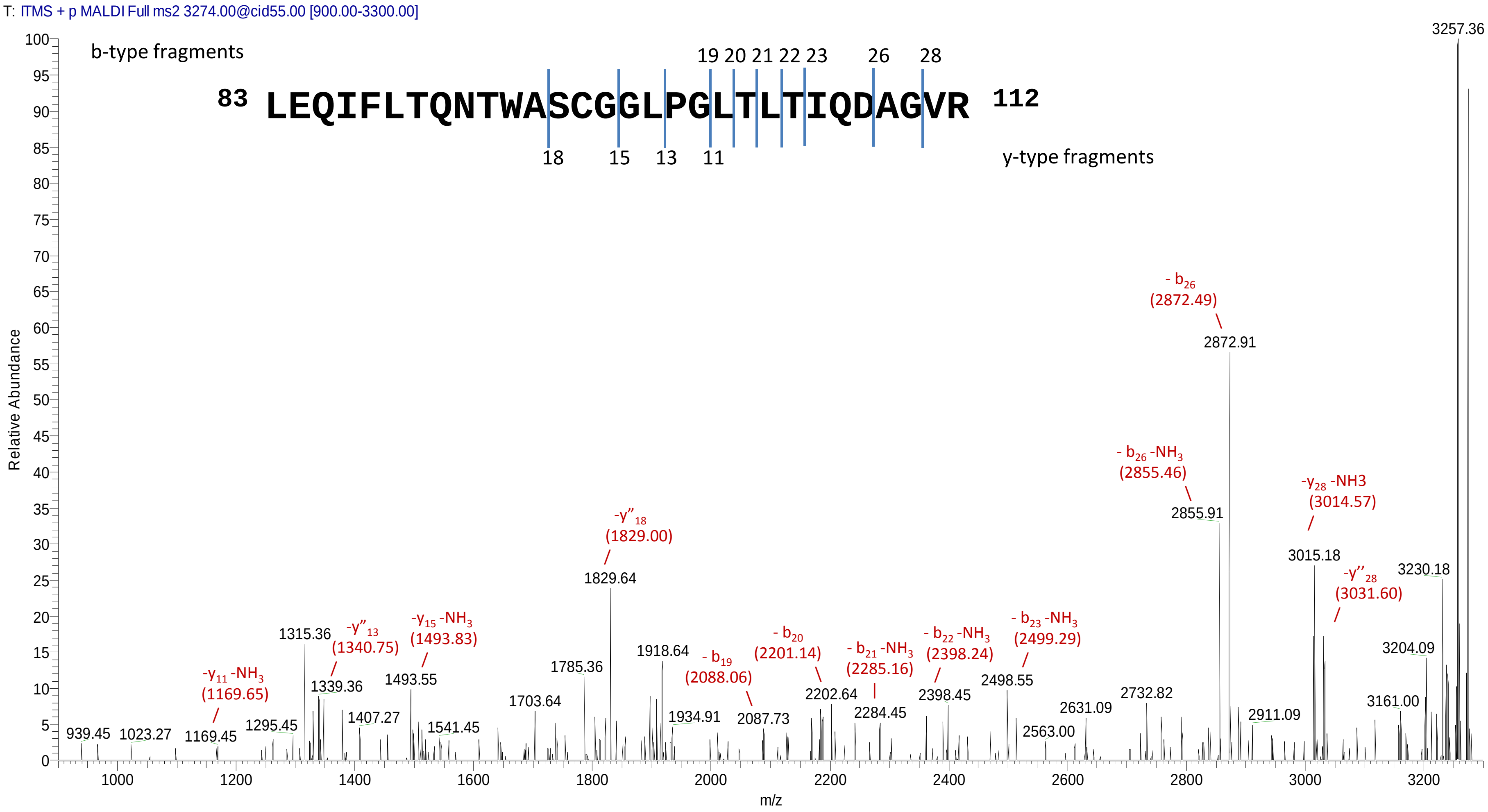

39. MS^2 m/z 3458

Theoretical mass: 3456.64 Da 286-316, *D. melanogaster*

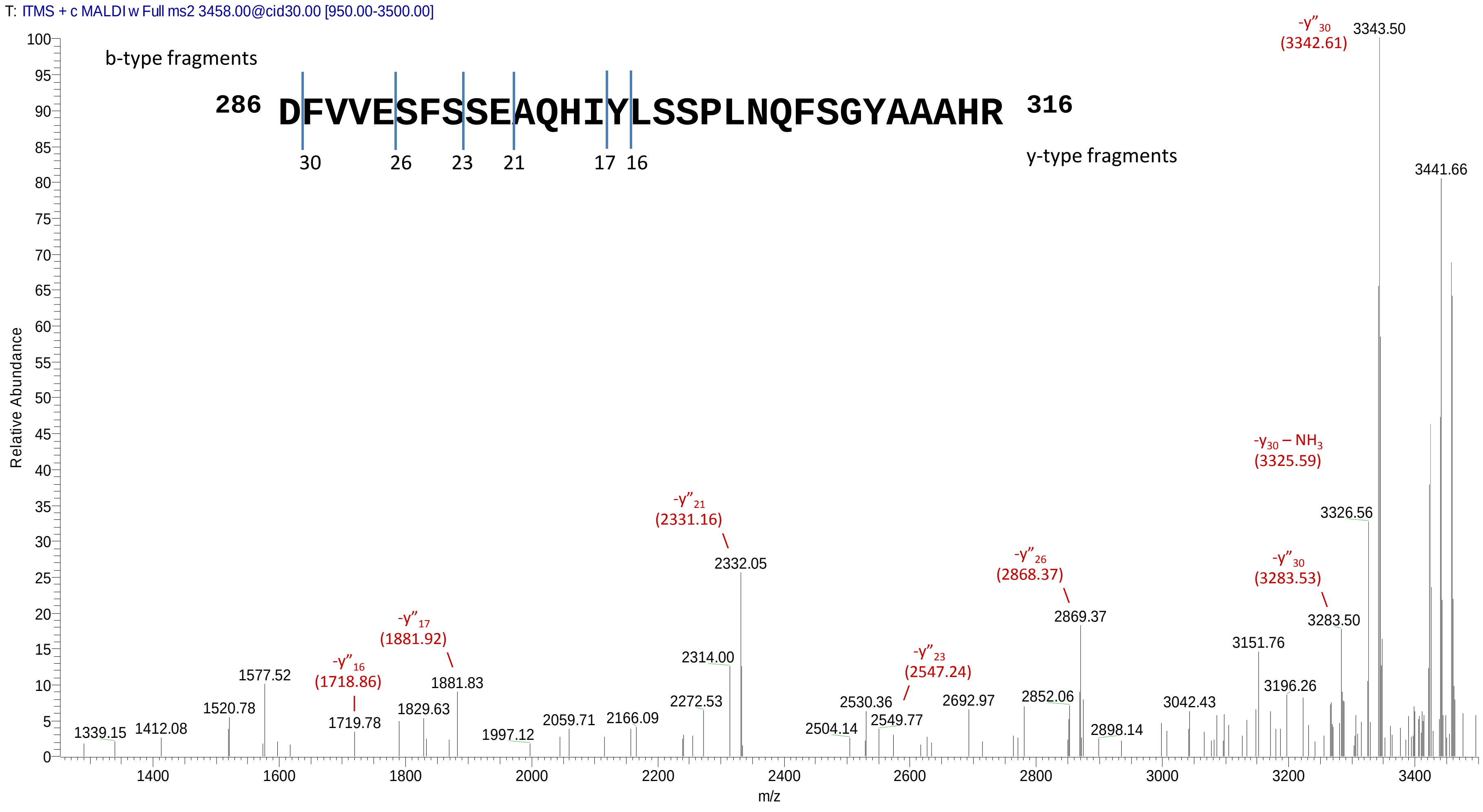

40. MS^2 m/z 3551

Theoretical mass: 3548.79 Da 222-254, *D. melanogaster*

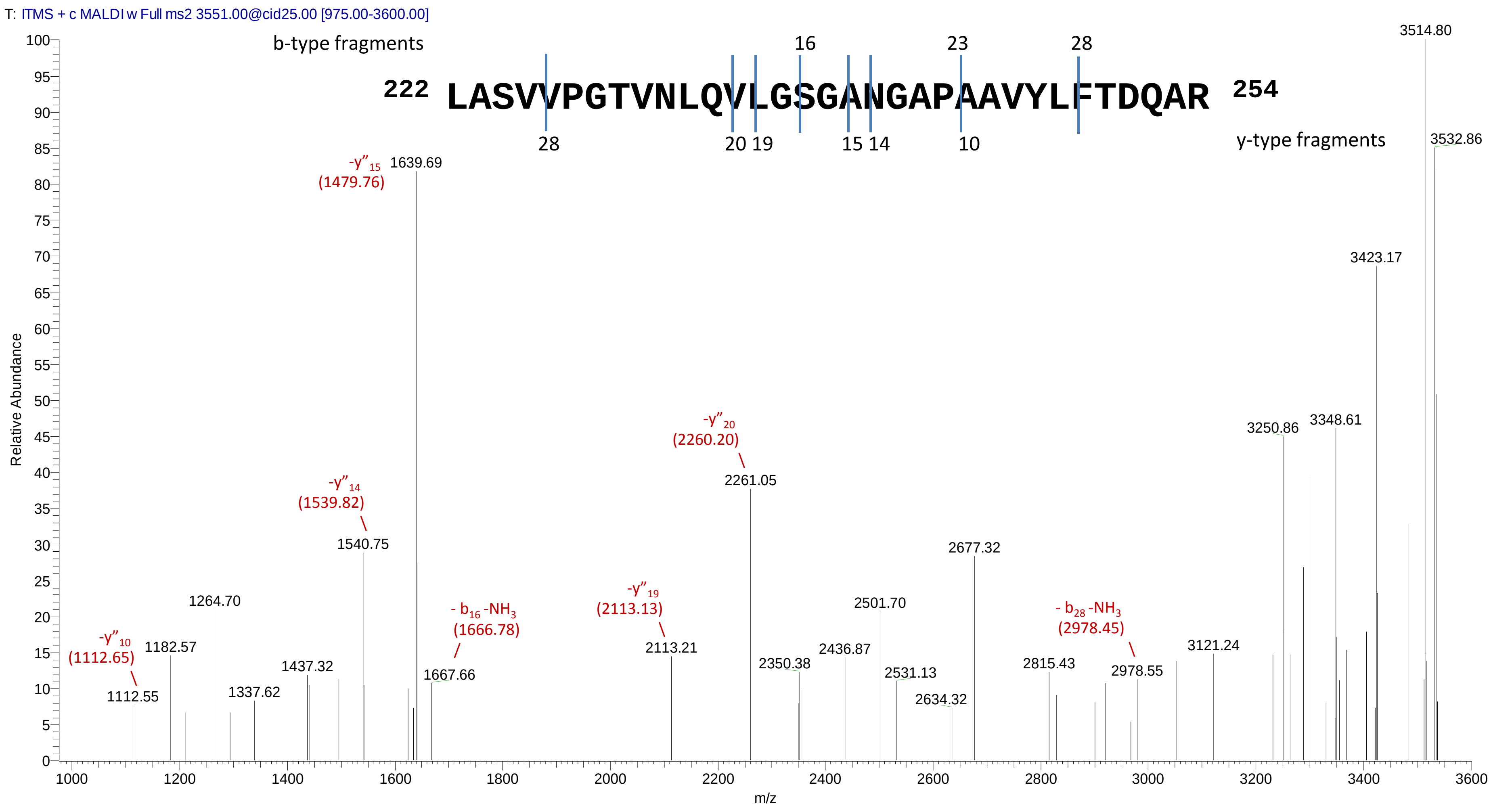

Supplement: Appendix S1 — MALDI-Ion trap MS/MS spectra from D. melanogaster tRNAseZ. (PDF) [file pone.0066942.s001.pdf]
