# Supplementary material for: Tethered Domains and Flexible Regions in tRNase ZL, the Long Form of tRNase Z
Source: PLoS One. 2013 Jul 17;8(7):e66942. doi: 10.1371/journal.pone.0066942 (PMC3714273; doi:10.1371/journal.pone.0066942)

H. sapiens tRNase Z^L

Supplemental Appendix 2. Panels 1-33 provide typical examples of MALDI-ion trap MS/MS spectra that confirm the amino acid sequences of the *H. sapiens* tRNaseZ^L peptides obtained by exhaustive digestion of 2D gel spots as represented in Supplemental Table ST9. Peaks are labeled in red, mainly as b-ion and c-ion derivatives (N-terminal fragments, numbering from N-terminal end, with cleavage at the peptide bond and c-terminal to peptide bond, respectively), and y-ion derivatives (C-terminal fragments, numbering from C-terminal end, cleavage at peptide bond). Theoretical m/z values of fragment ions are shown in parentheses. Cysteines are modified *in situ* with acrylamide from the 2D gel preparation, to produce S-proprionamidated species). Under MS/MS conditions, these sometimes gave rise to neutral loss products where the proprionamide cysteine modification areis lost as formal C_3H_4ON and C_3H_6ONS and related species (*e.g.* panels 17). All assignments were made manually.

1. MS^2 m/z 801

Theoretical mass: 800.51 Da 624-630, *H. sapiens*

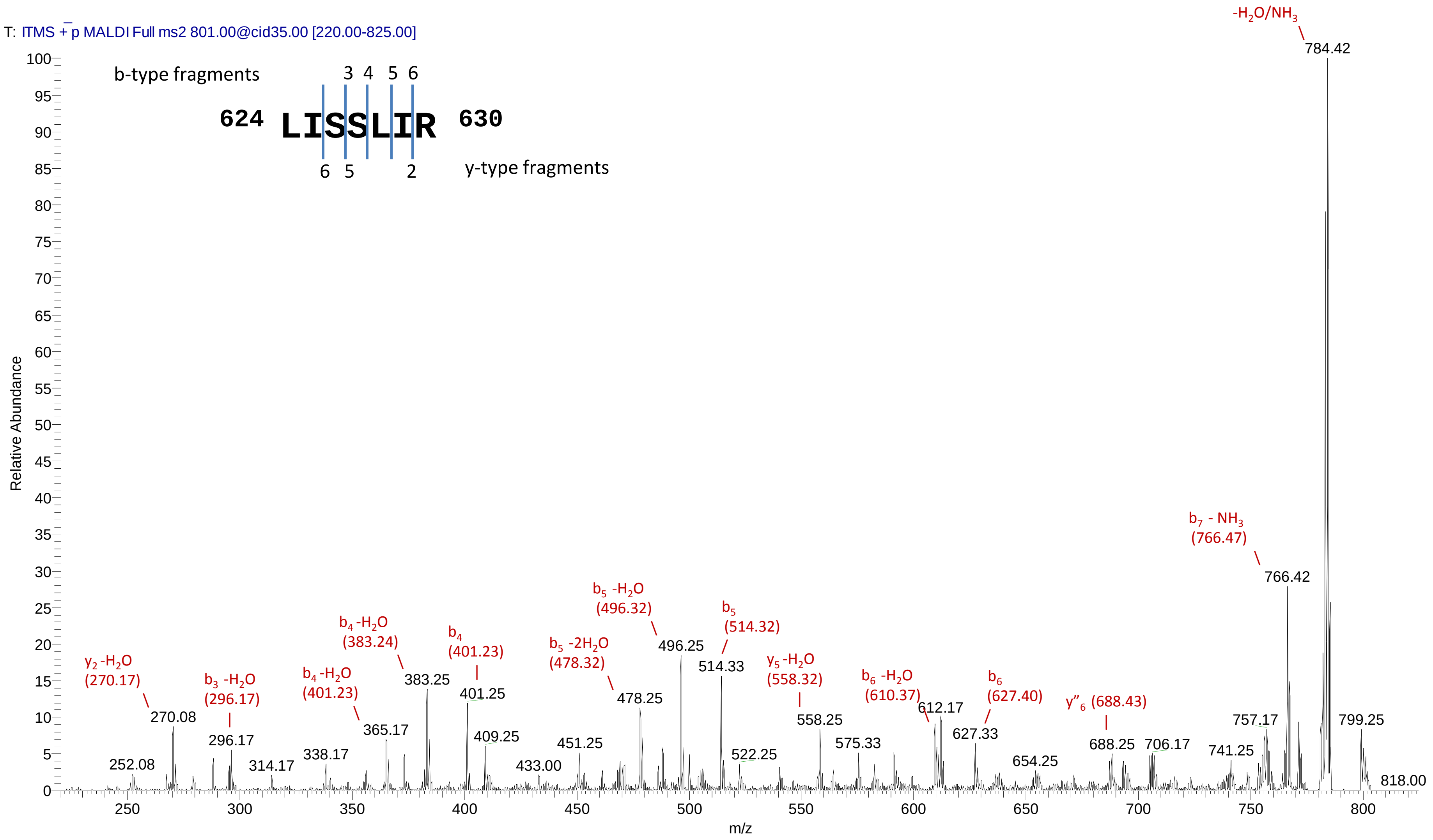

2. MS^2 m/z 832

Theoretical mass: 831.47 Da 425-430, *H. sapiens*

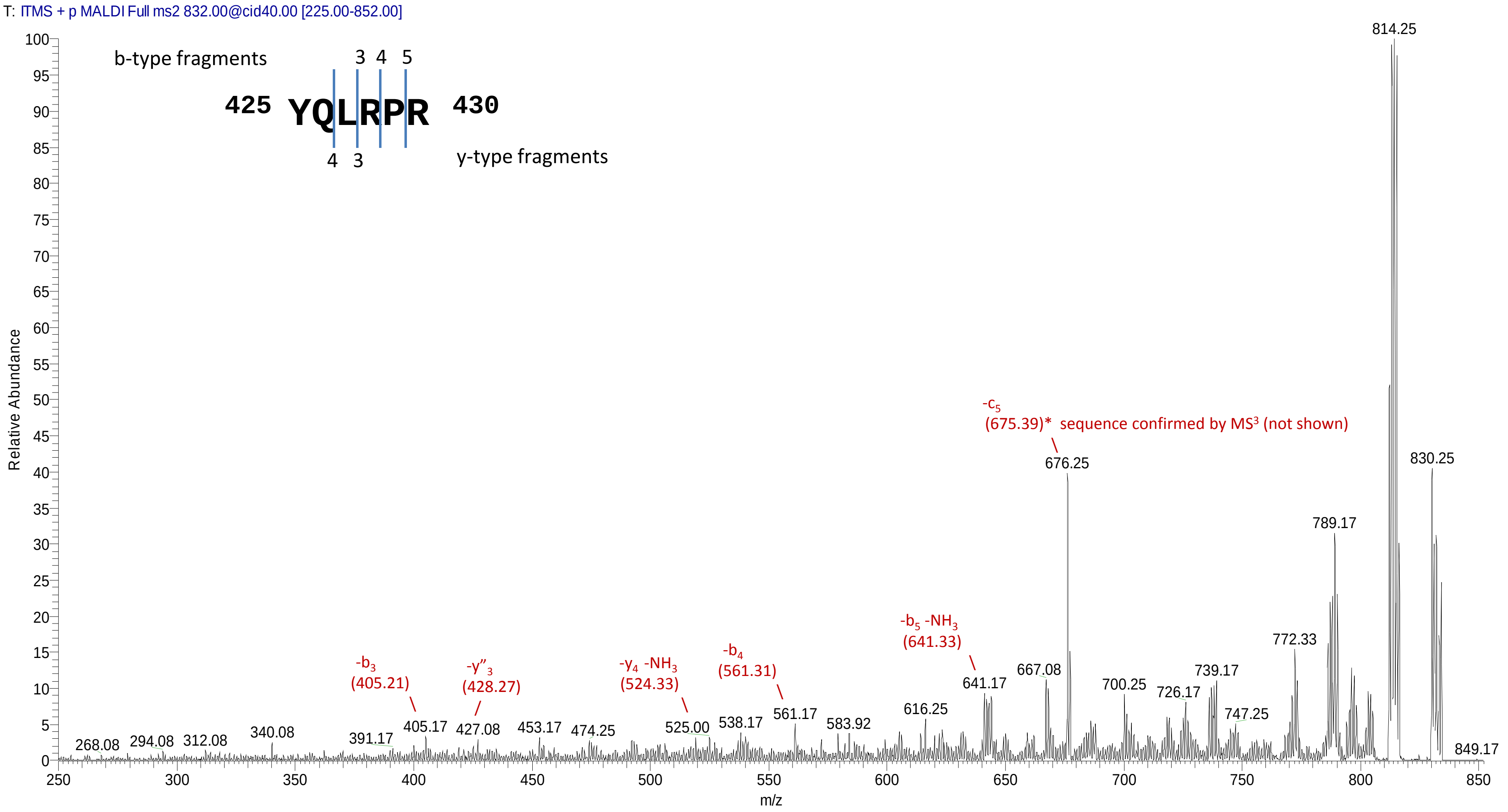

3. MS^2 m/z 990

Theoretical mass: 990.55 Da 105-112, *H. sapiens*

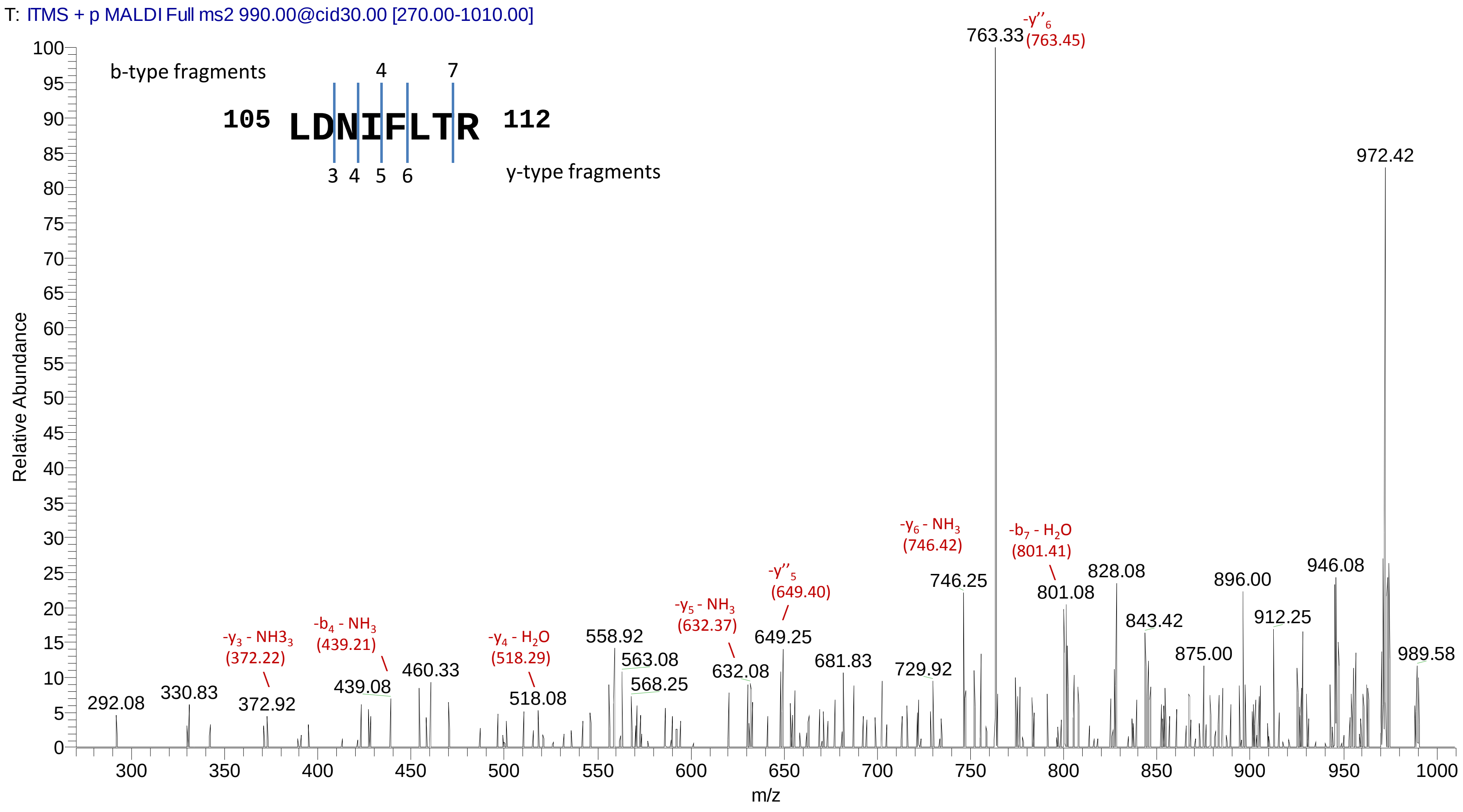

4. MS^2 m/z 1003

Theoretical mass: 1002.50 Da 741-749, *H. sapiens*

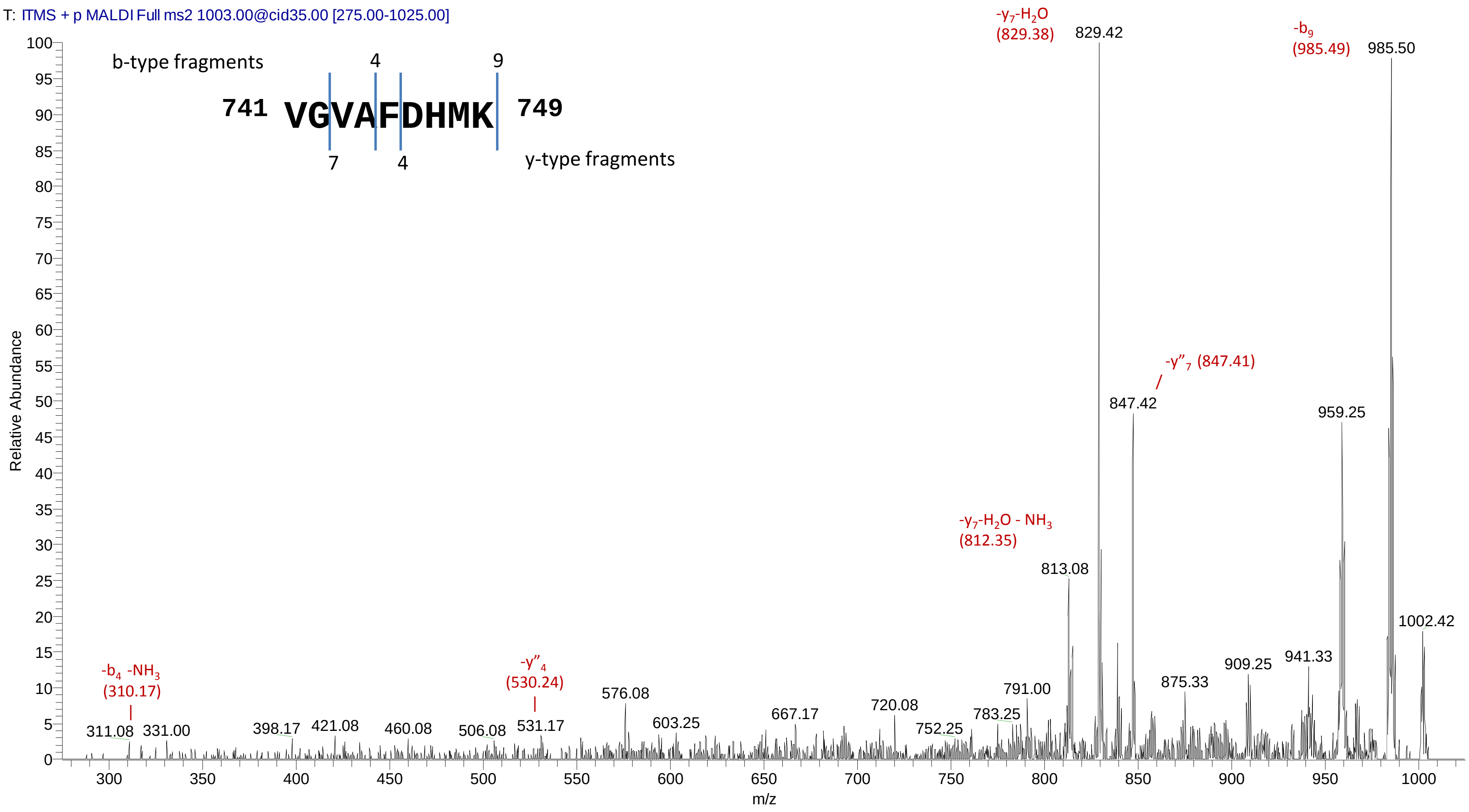

5. MS^2 m/z 1039

Theoretical mass: 1039.45 Da 354-360, *H. sapiens*

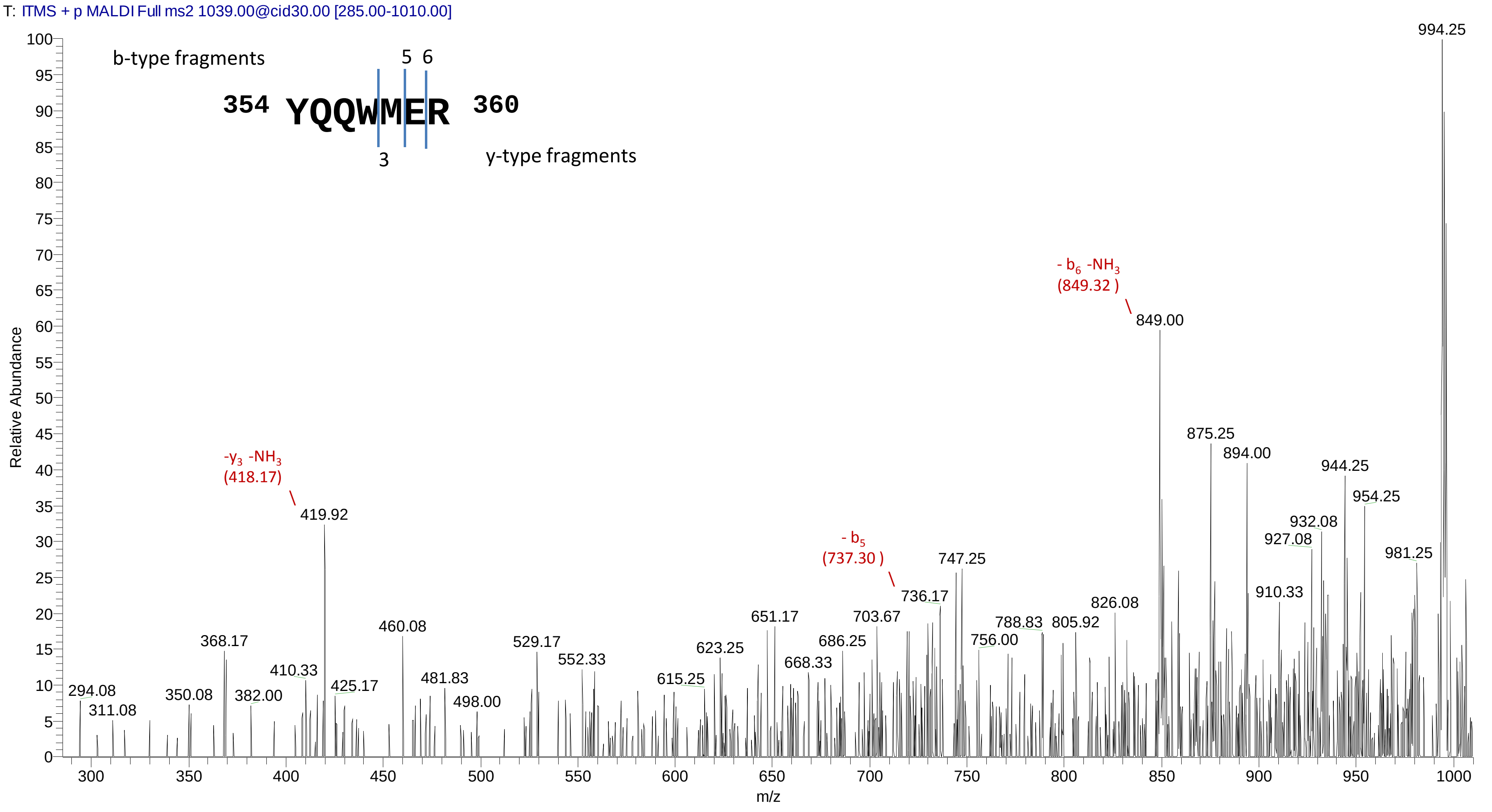

6. MS^2 m/z 1241

Theoretical mass: 1240.56 Da 136-146, *H. sapiens*

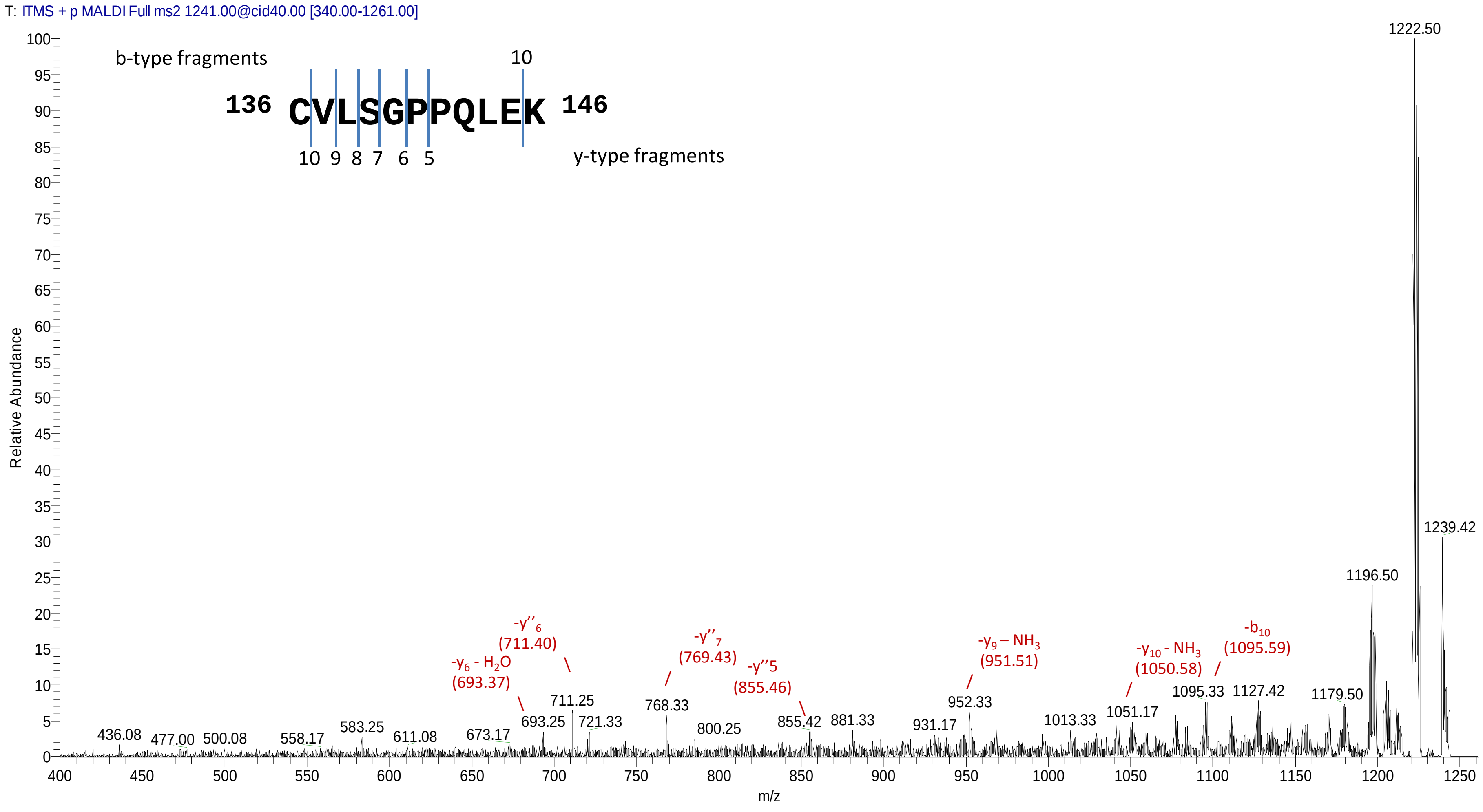

7. MS^2 m/z 1264

Theoretical mass: 1263.65 Da 732-742, *H. sapiens*

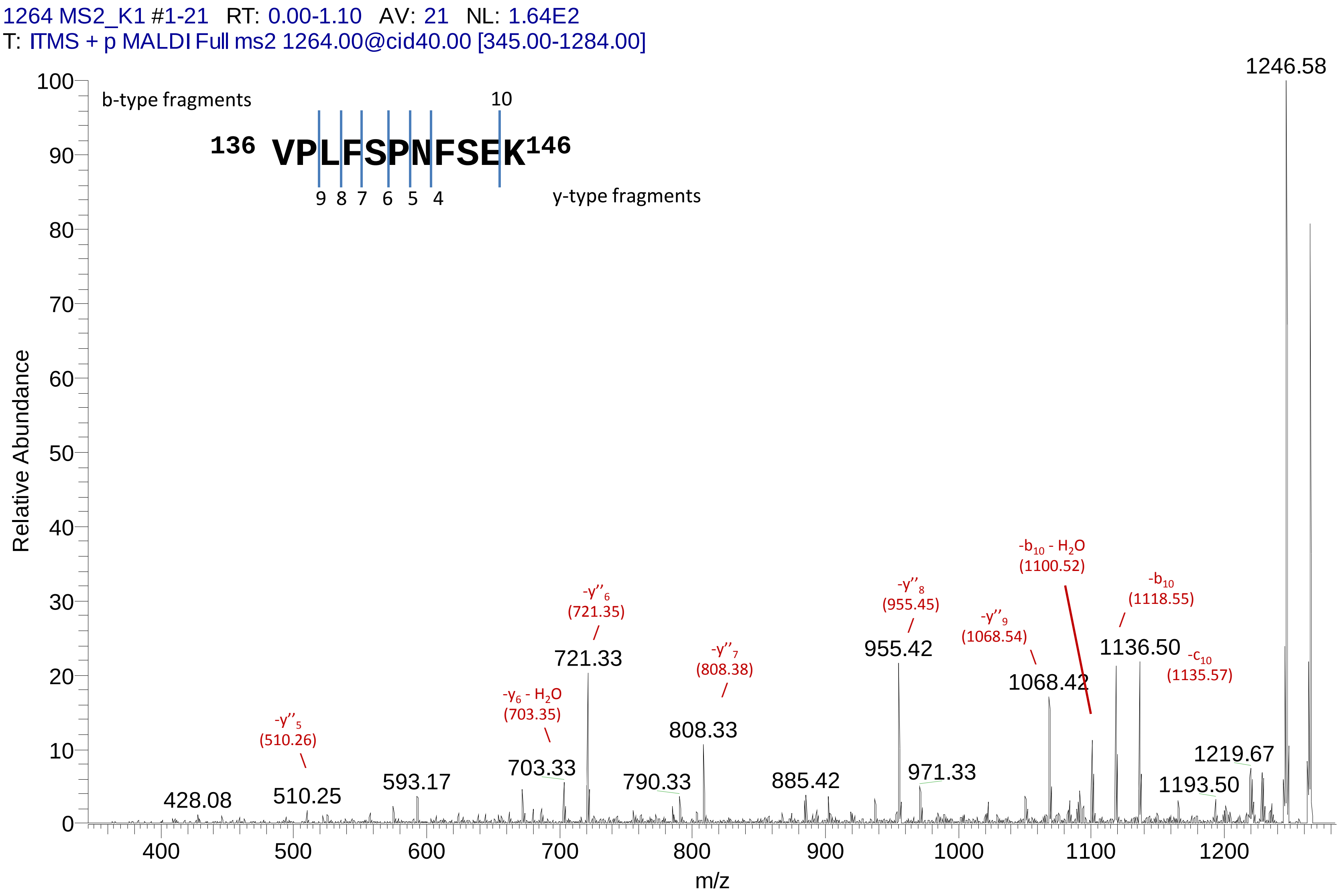

8. MS^2 m/z 1253

Theoretical mass: 1251.65 Da 236-246, *H. sapiens*

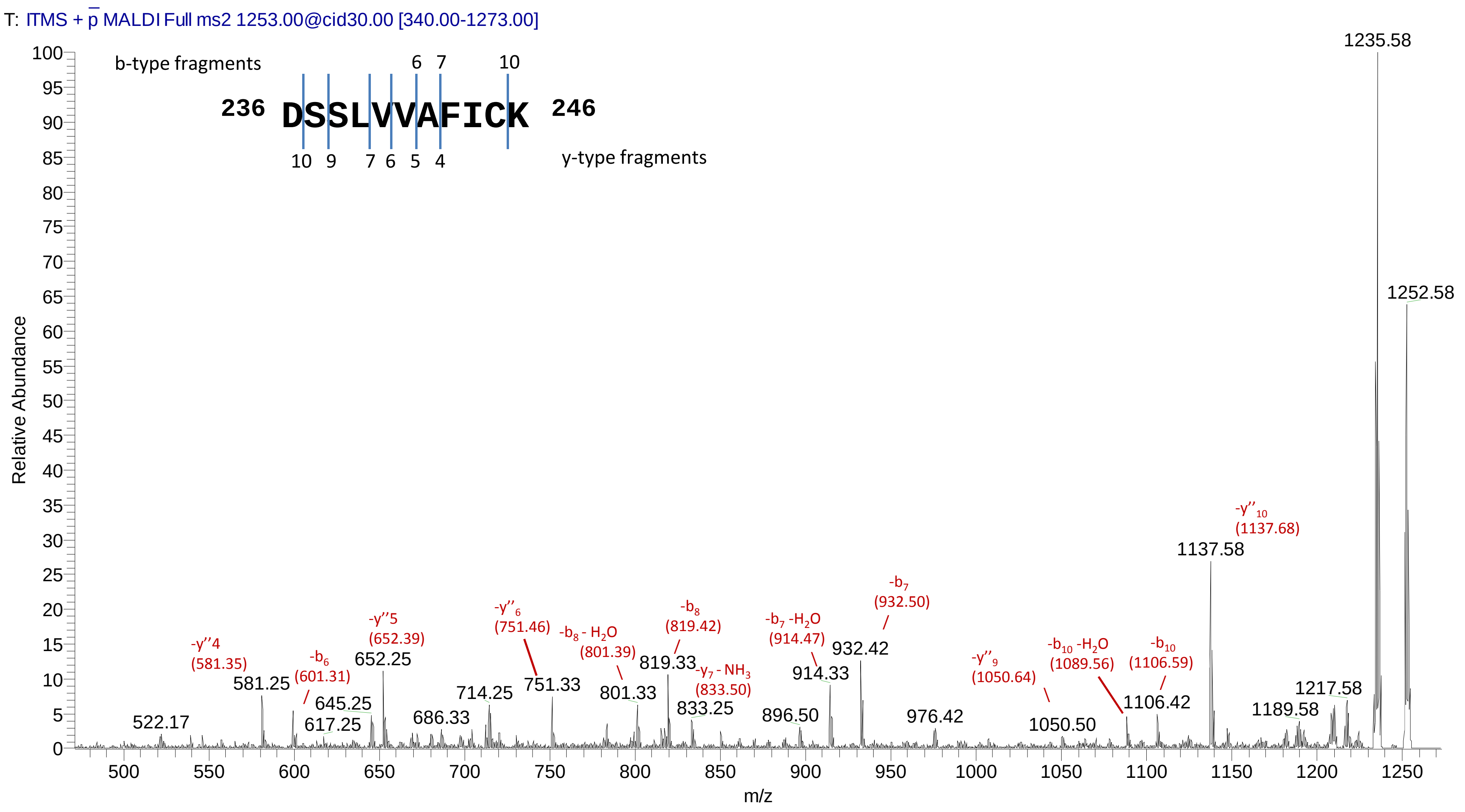

9. MS^2 m/z 1311

Theoretical mass: 1311.60 Da 752-762, *H. sapiens*

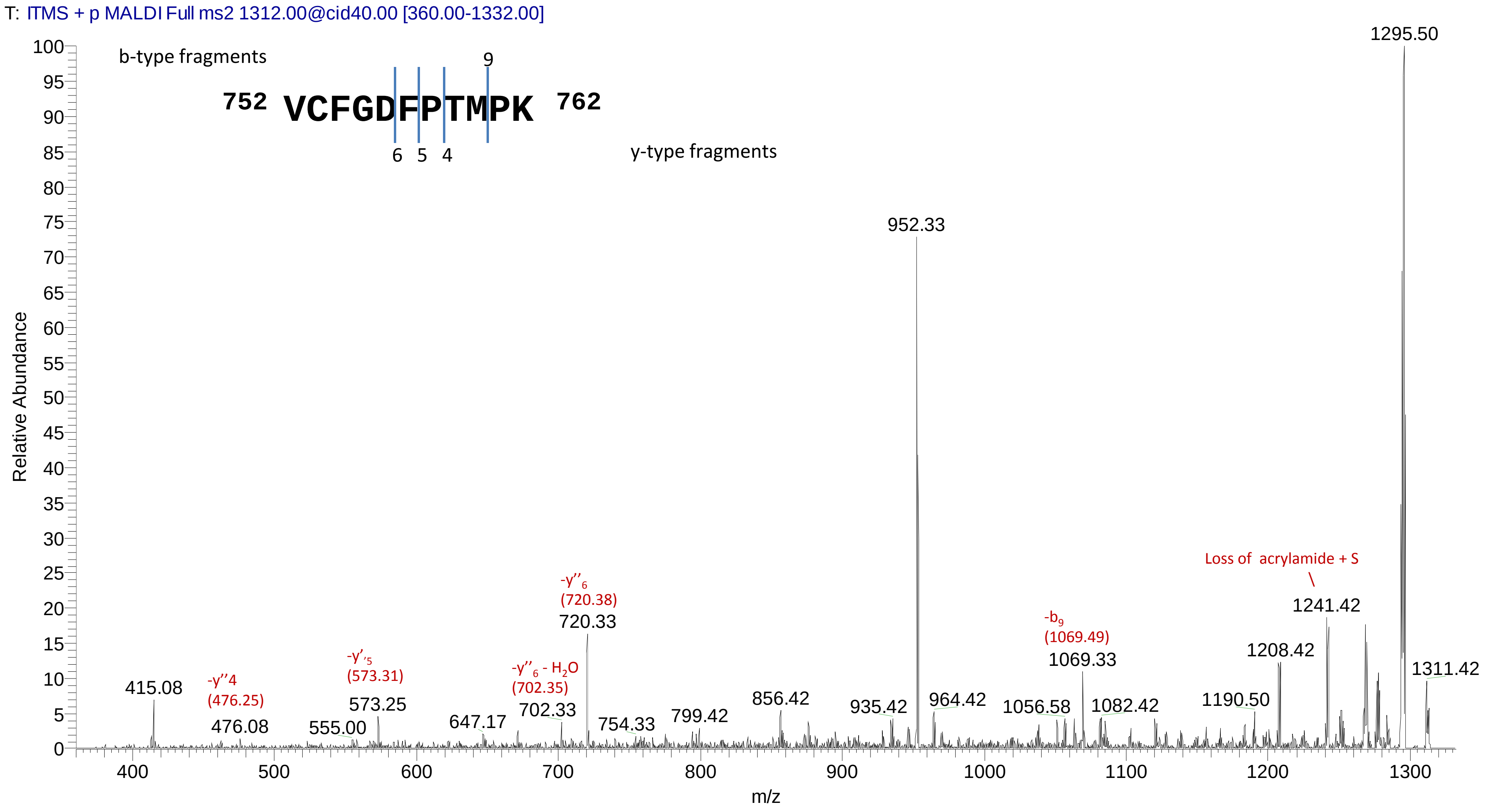

10. MS^2 m/z 1357

Theoretical mass: 1355.63 Da 83-93, *H. sapiens*

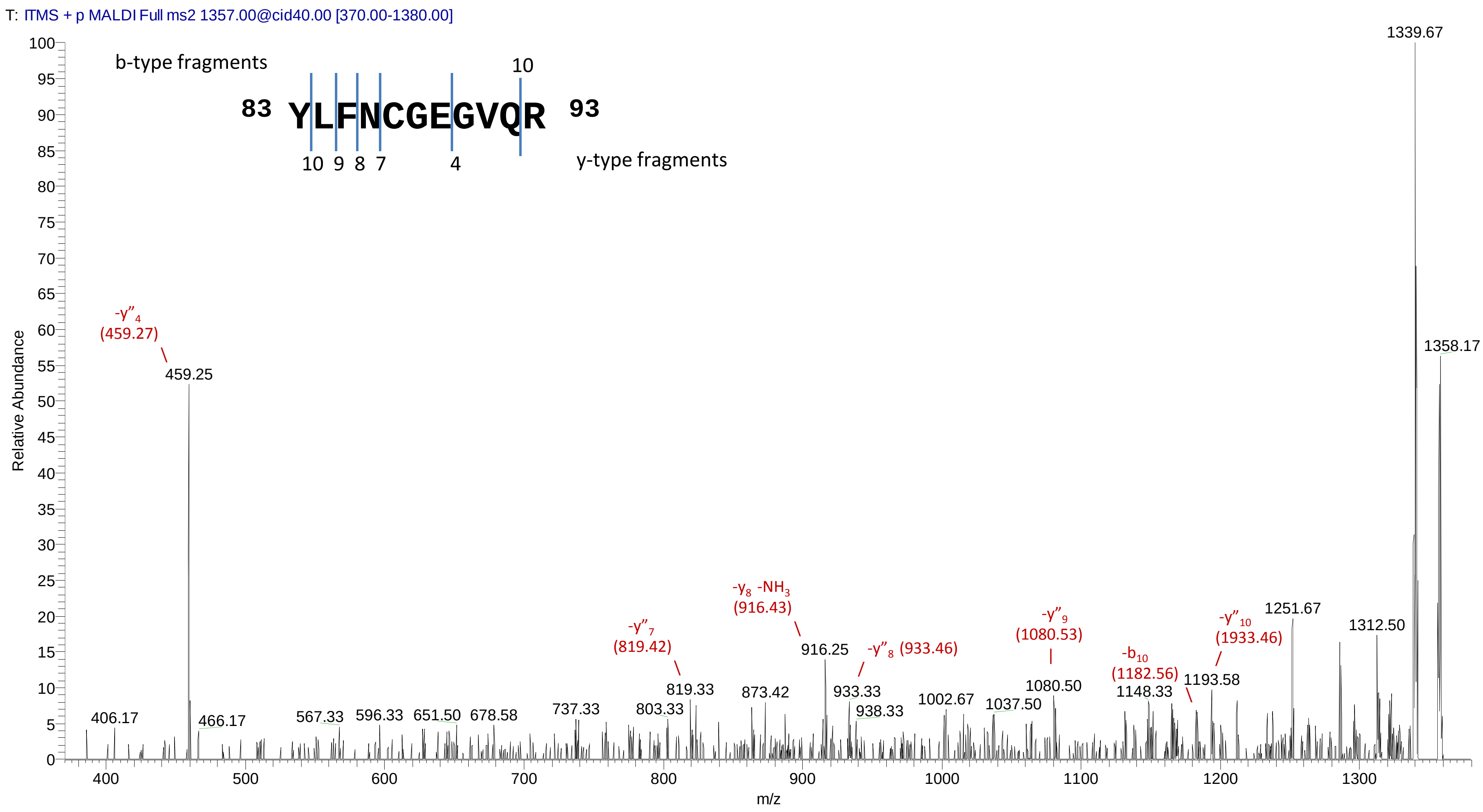

11. MS^2 m/z 1475

Theoretical mass: 1474.72 Da 701-714, *H. sapiens*

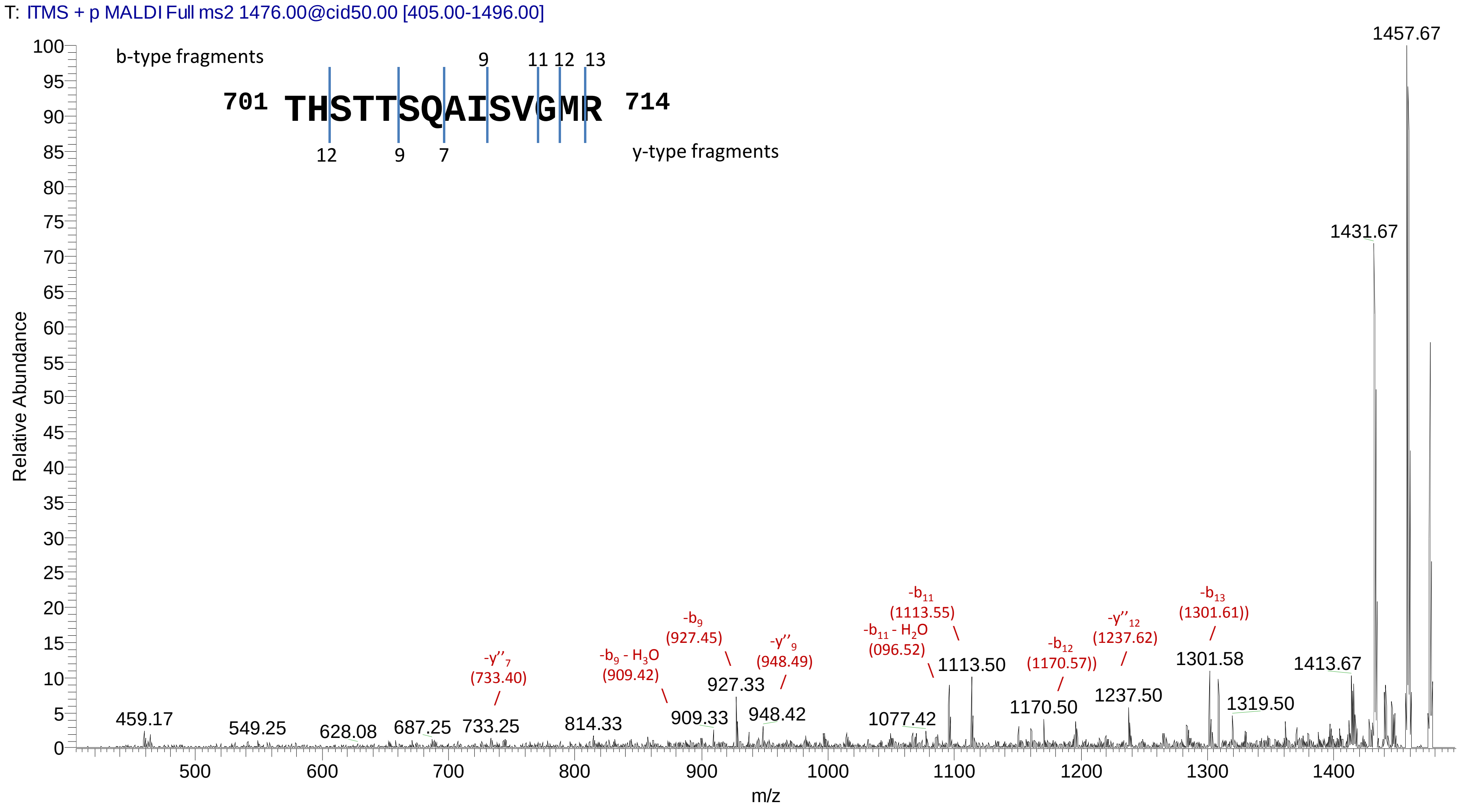

12. MS^2 m/z 1508

Theoretical mass: 1508.68 Da 769-781, *H. sapiens*

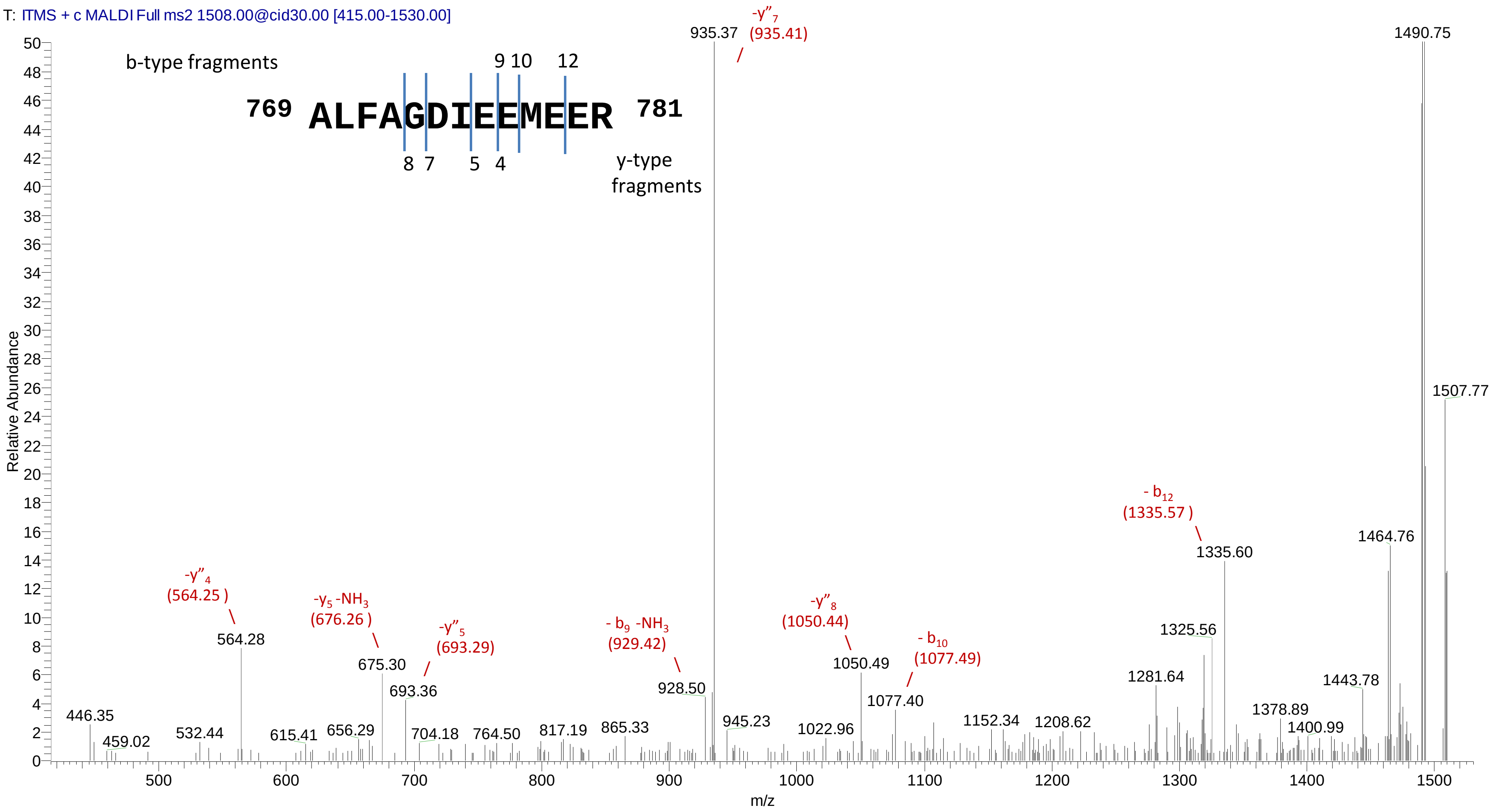

13.MS^2 m/z 1575

Theoretical mass: 1574.74 Da 69-82, *H. sapiens*

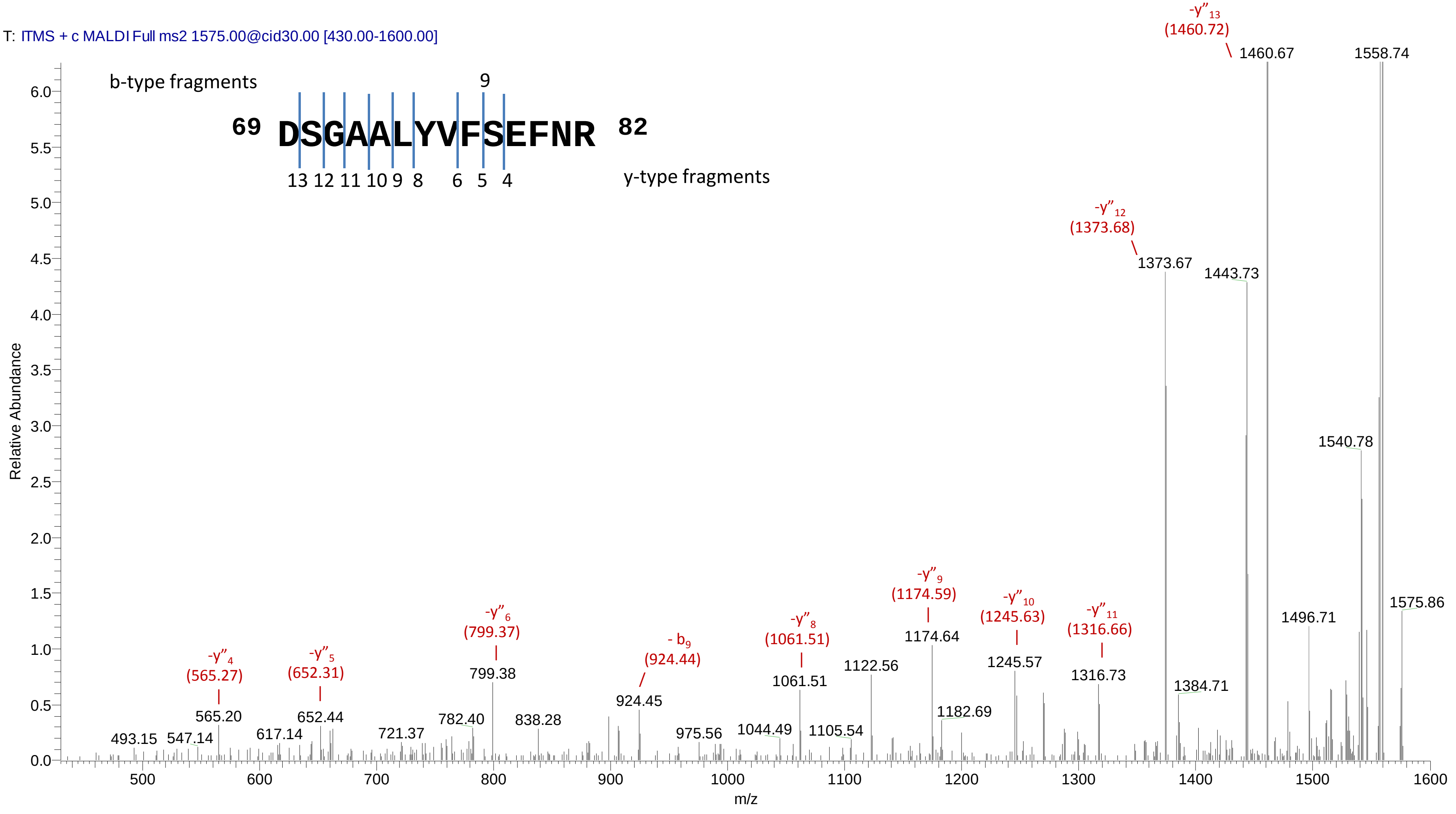

14. MS^2 m/z 1585

Theoretical mass: 1583.81 Da 647-660, *H. sapiens*

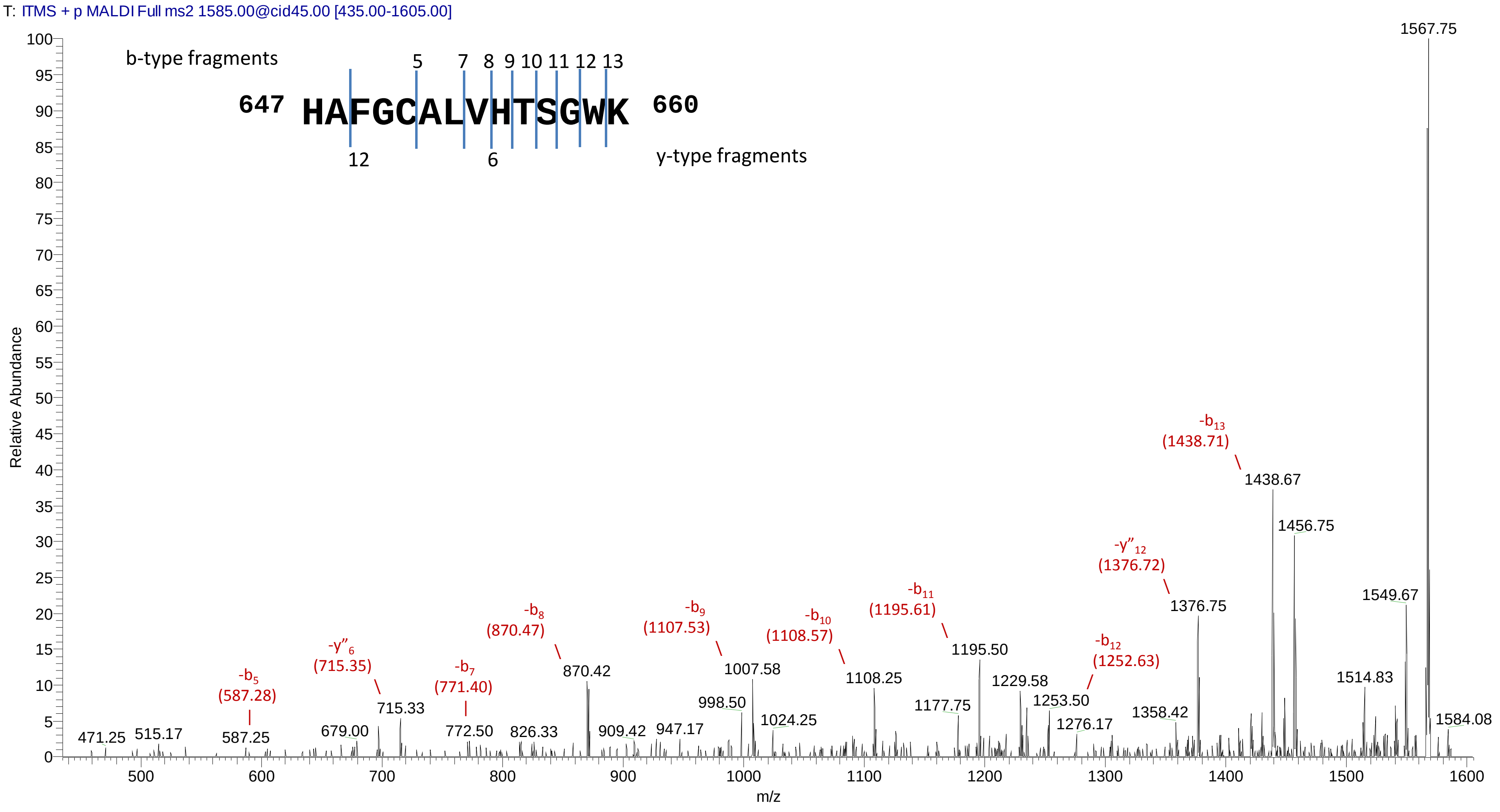

15. MS^2 m/z 1659

Theoretical mass: 1658.84 Da 609-623, *H. sapiens*

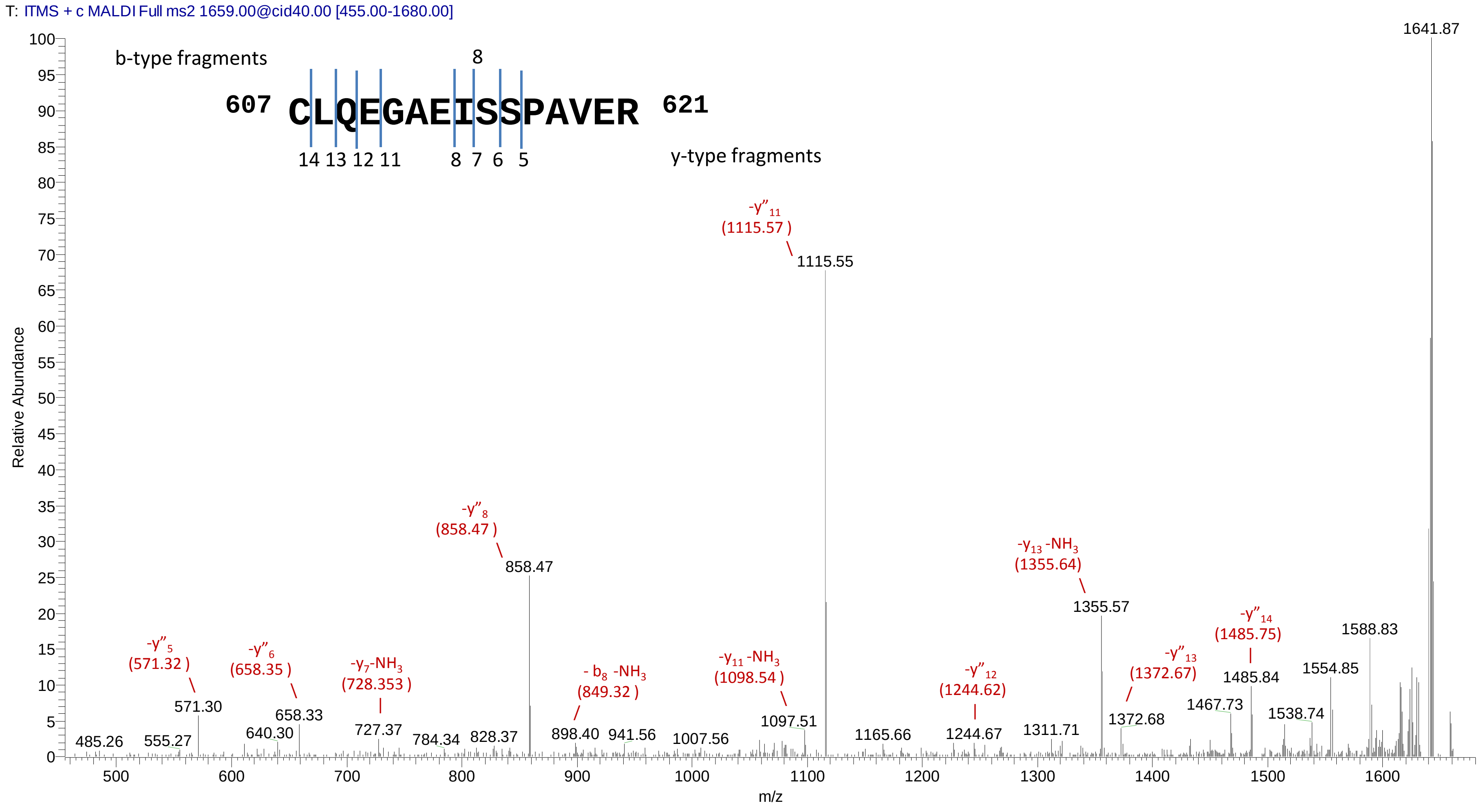

16. MS^2 m/z 1666

Theoretical mass: 1664.78 Da 769-782, *H. sapiens*

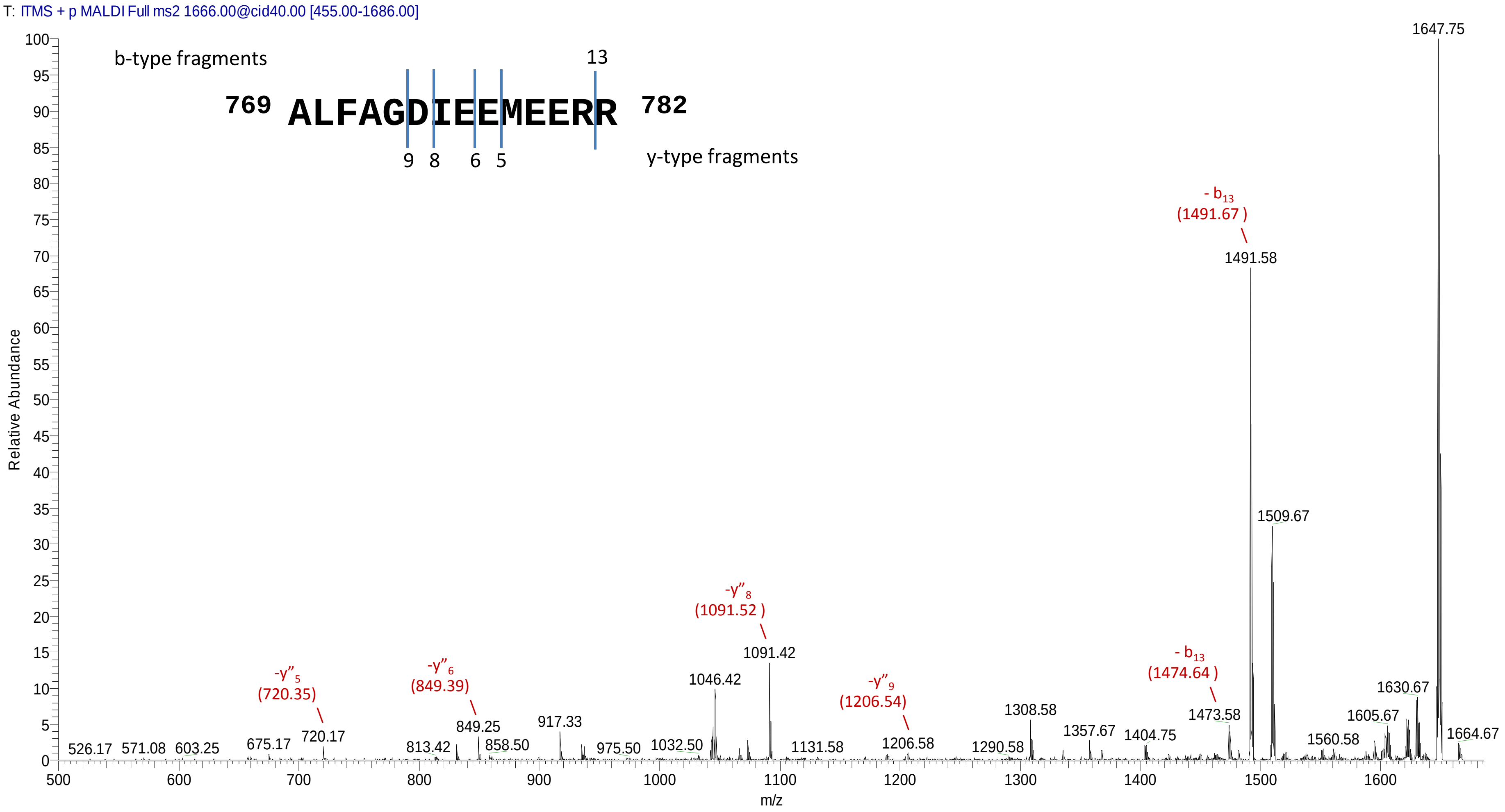

17. MS^2 m/z 1698

Theoretical mass: 1697.78 Da 631-643, *H. sapiens*

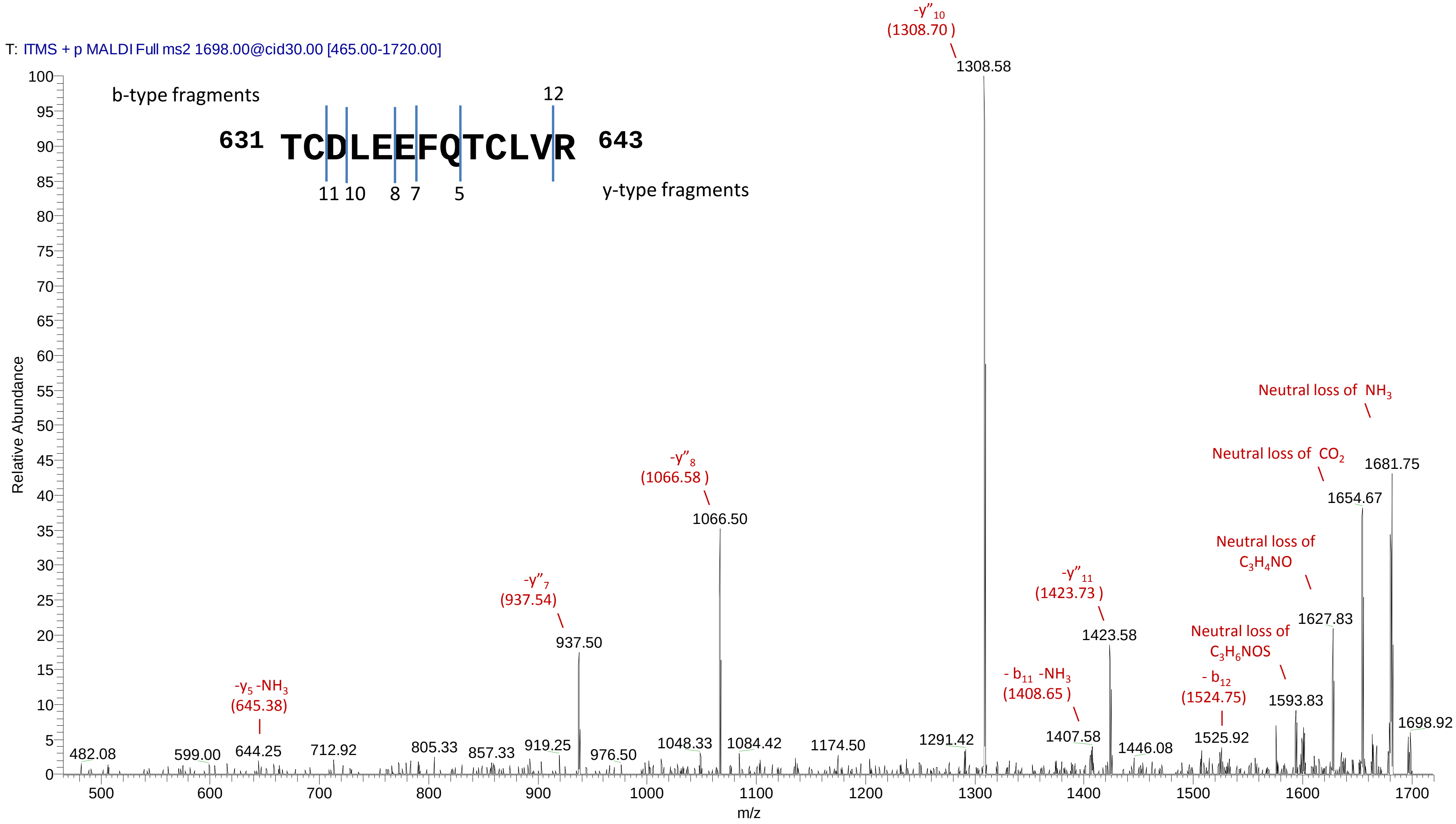

18.MS^2 m/z 1710

Theoretical mass: 1709.81 Da 661-675, *H. sapiens*

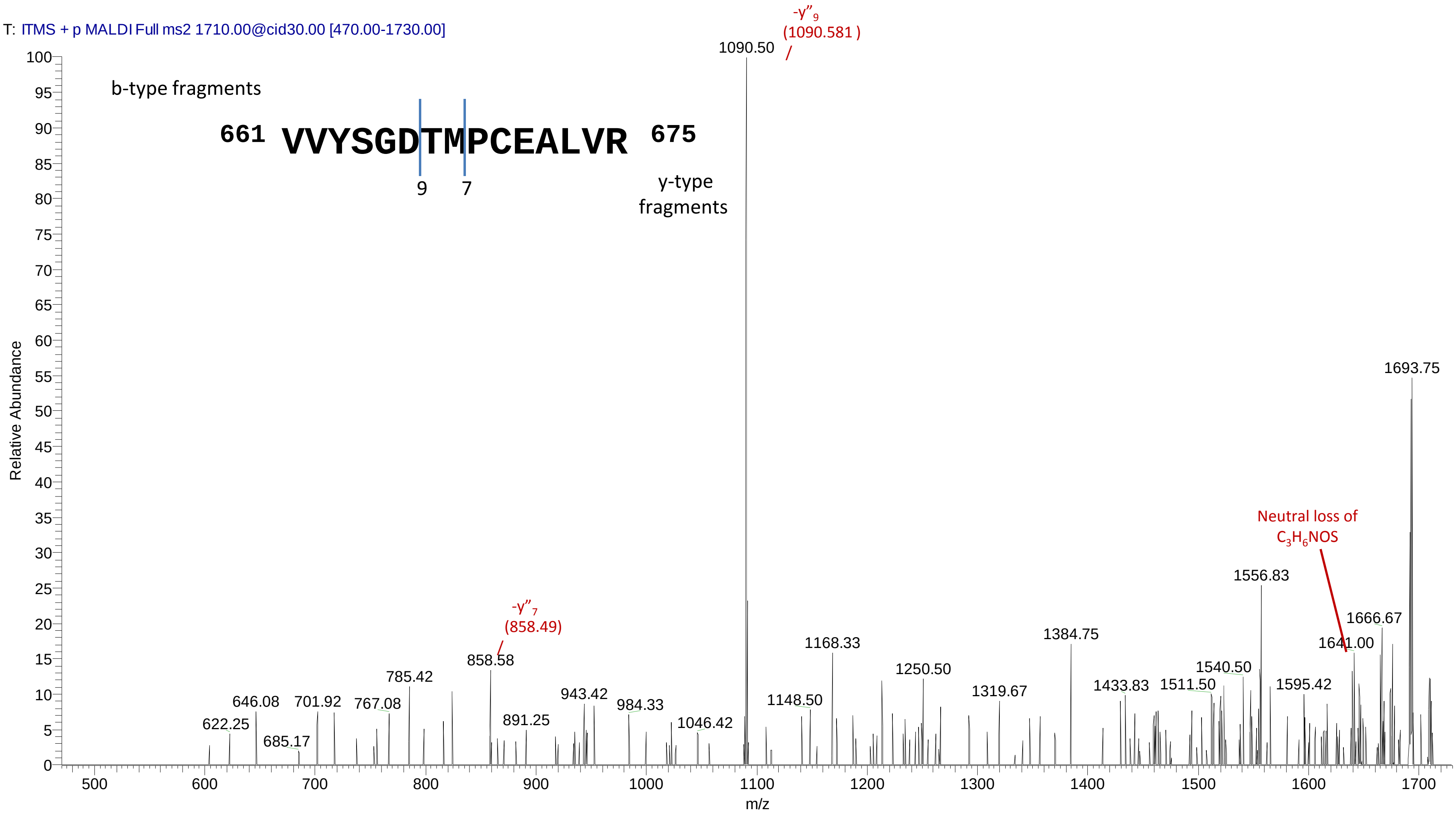

19. MS^2 m/z 1738

Theoretical mass: 1736.81 Da 715-728, *H. sapiens*

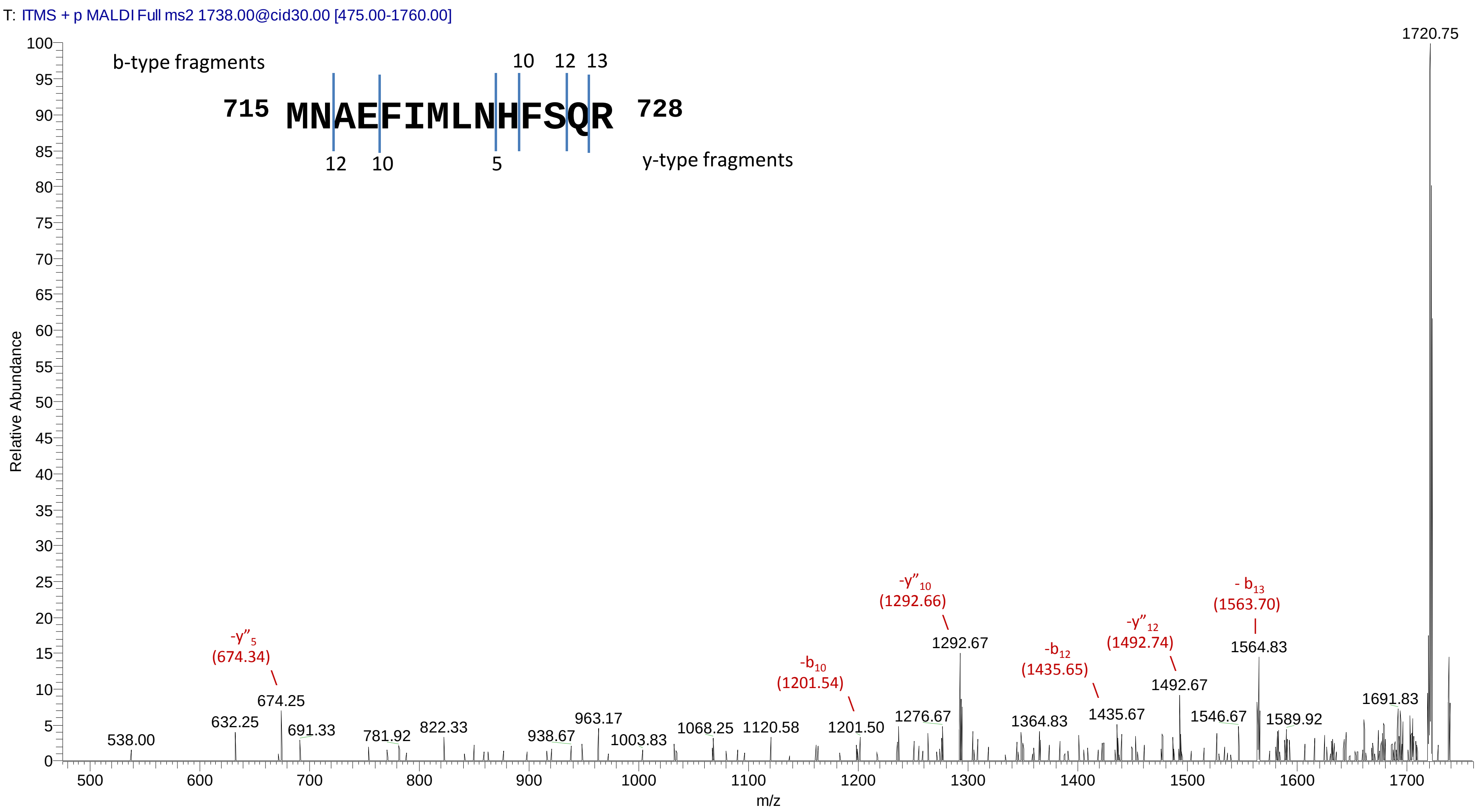

20. MS^2 m/z 1822

Theoretical mass: 1821.04 Da 261-279, *H. sapiens*

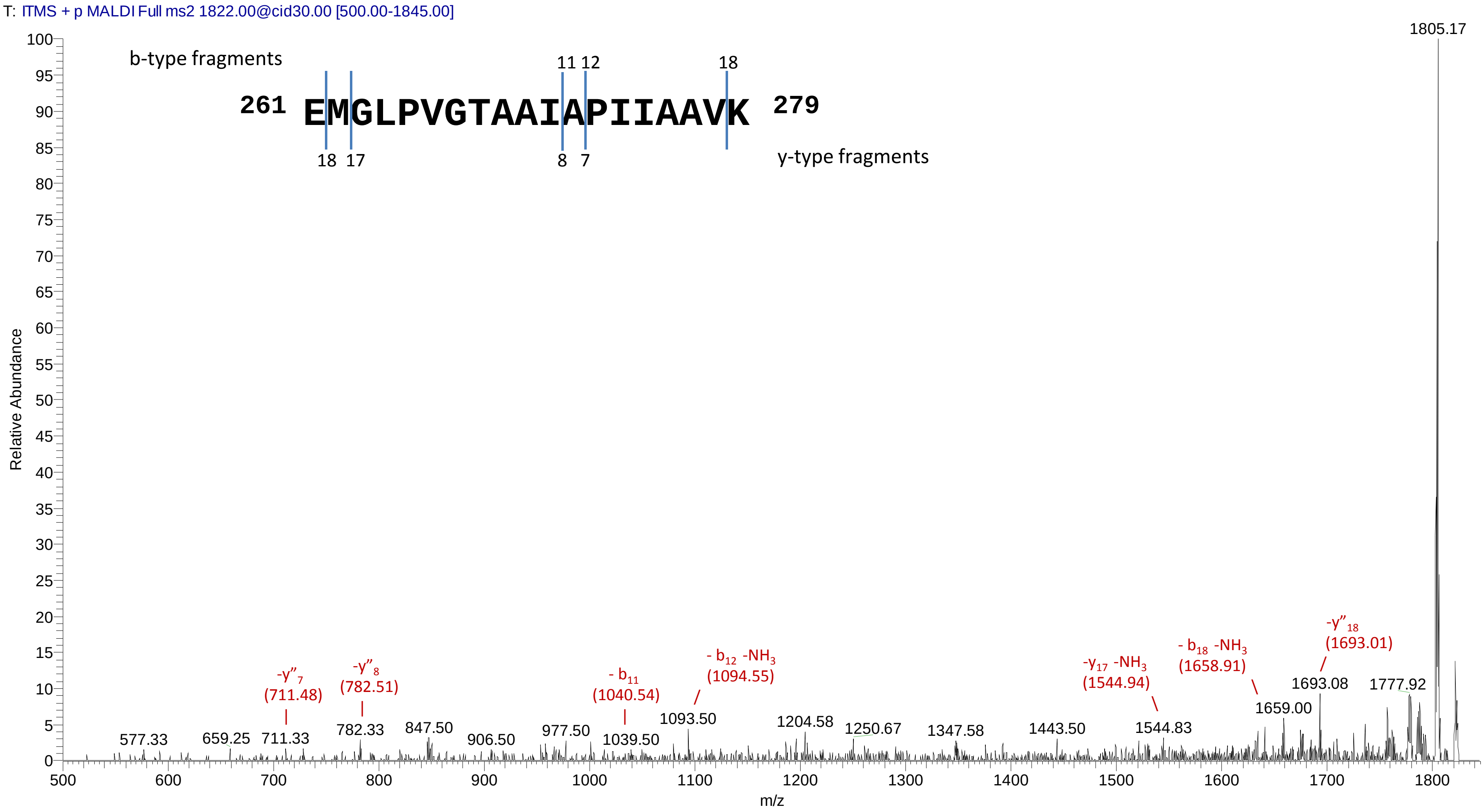

21. MS^2 m/z 1845

Theoretical mass: 1842.95 Da 113-129, *H. sapiens*

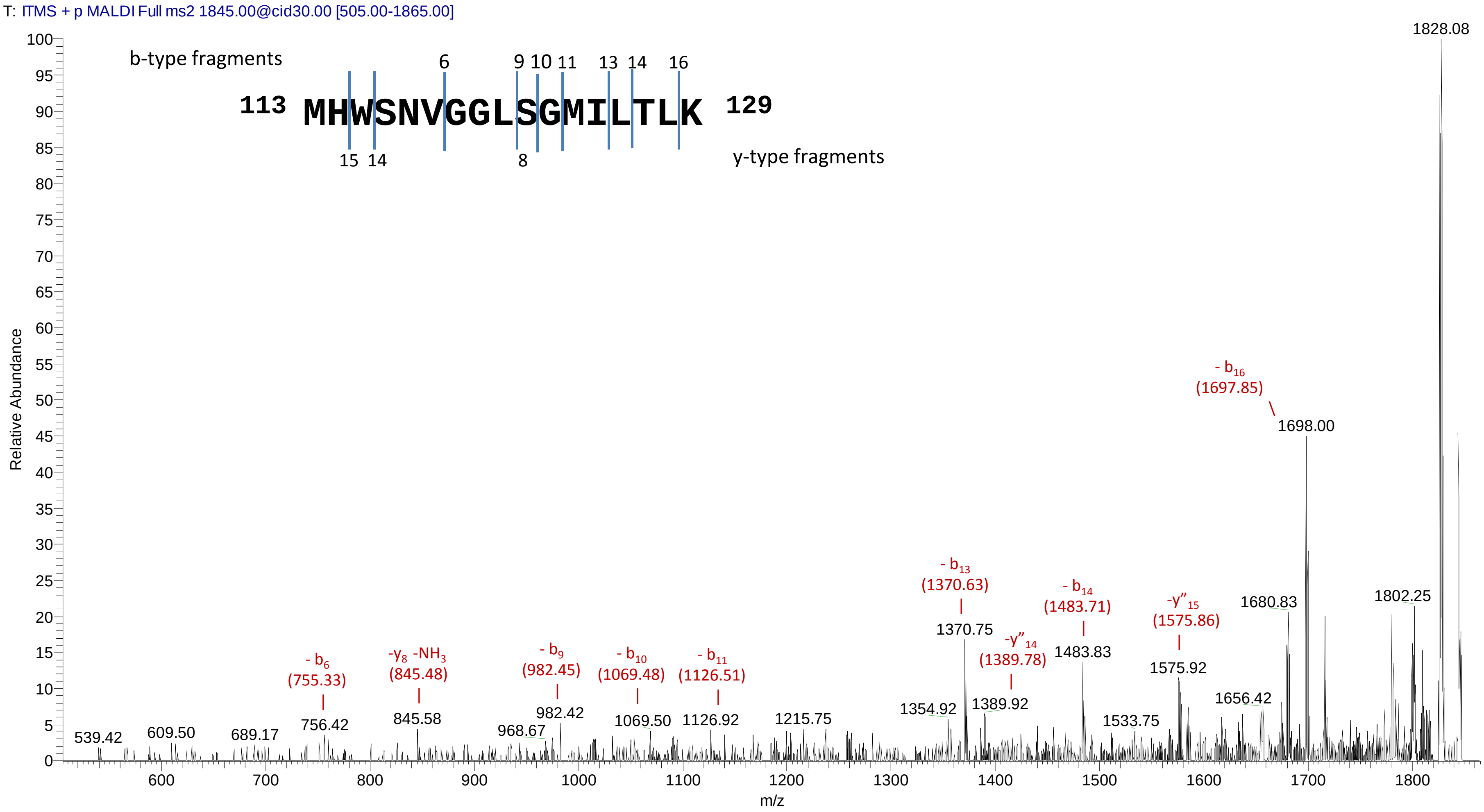

22. MS^2 m/z 1873. Y-axis amplified 5x from m/z 520-1750

Theoretical mass: 1870.95 Da 408-424, *H. sapiens*

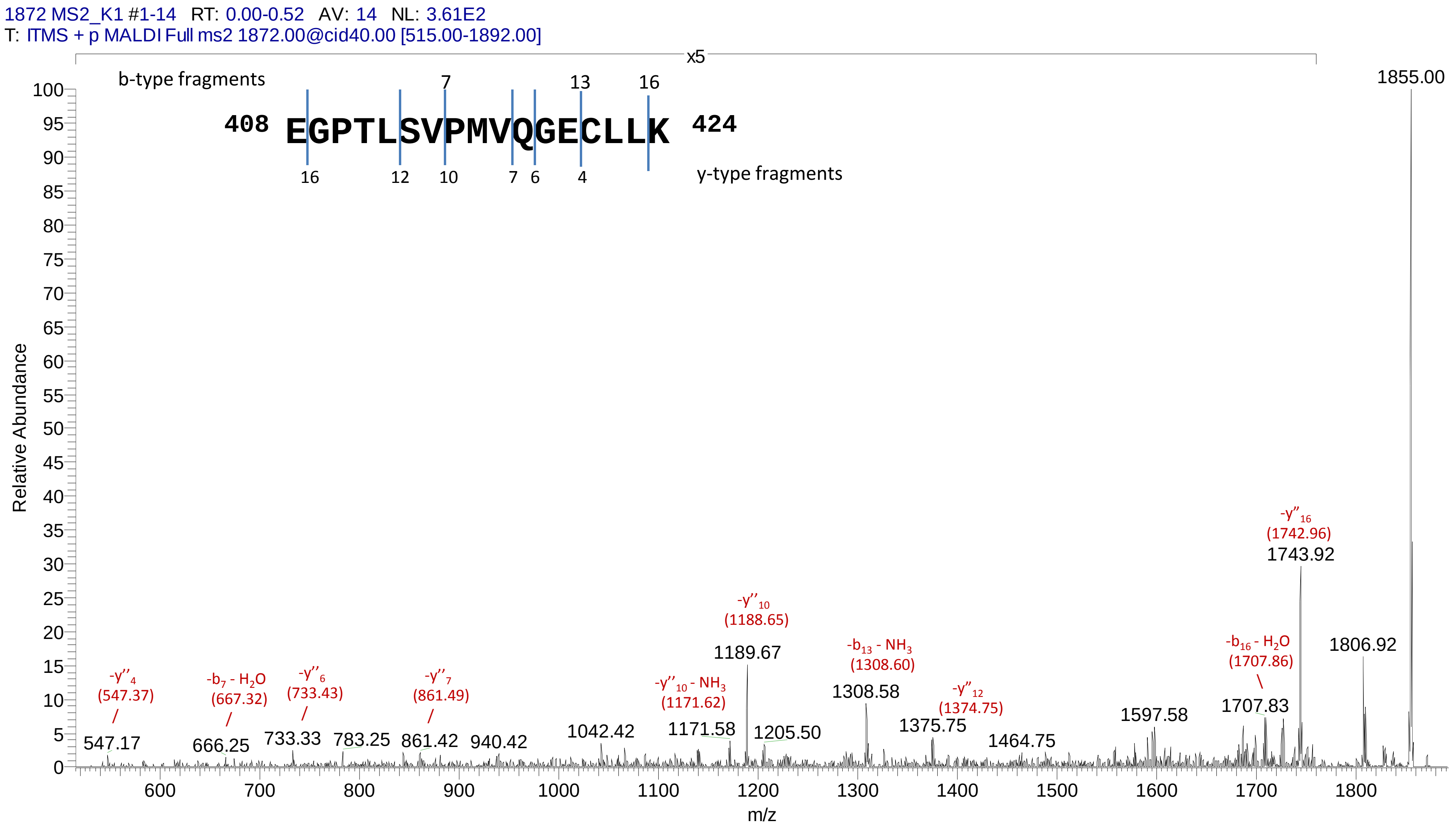

23. MS^2 m/z 1952

Theoretical mass: 1951.01 Da 478-495, *H. sapiens*

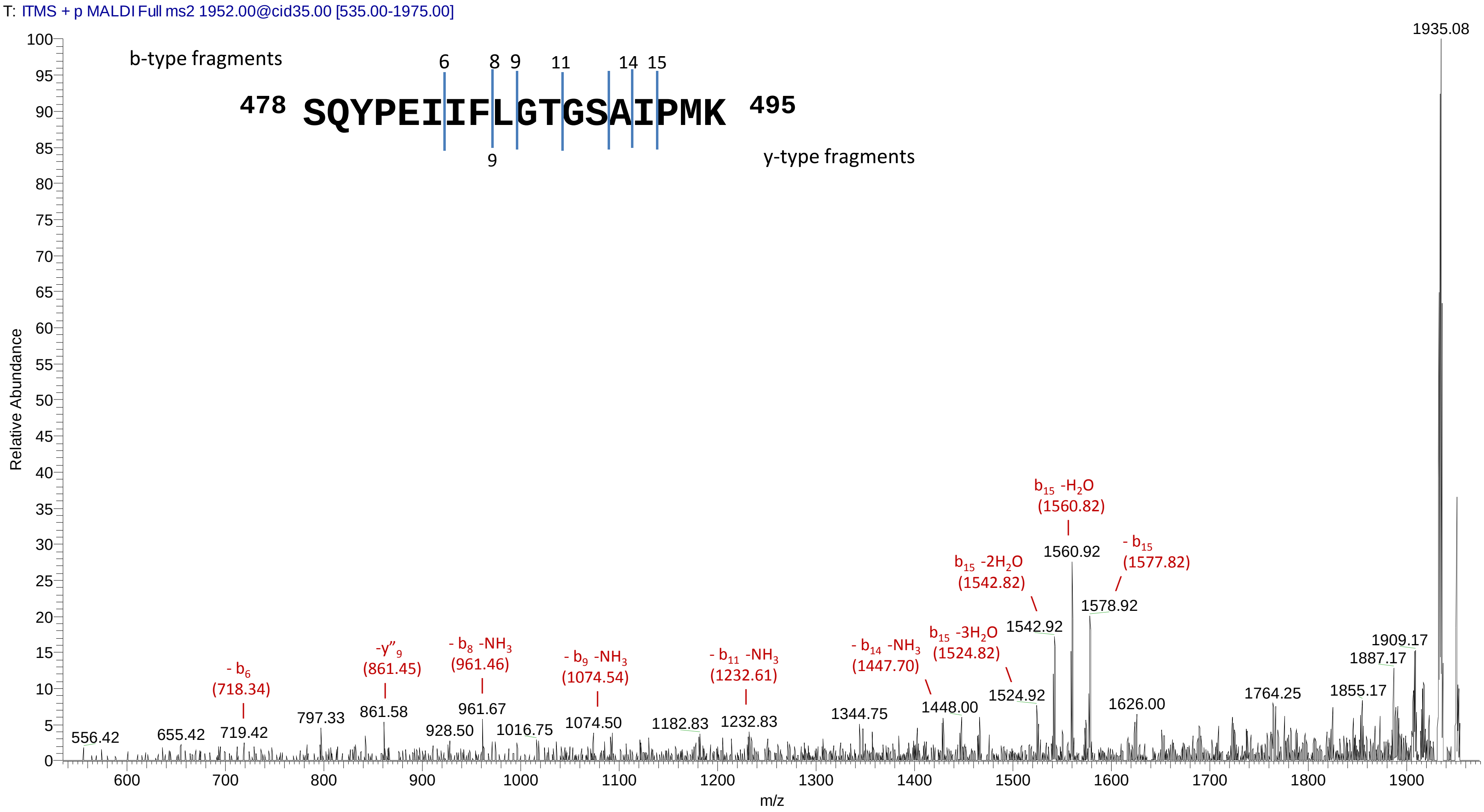

24. MS^2 m/z 2119 Y-axis amplified 25x from m/z 600-1650

Theoretical mass: 2117.03 Da 333-353, *H. sapiens*

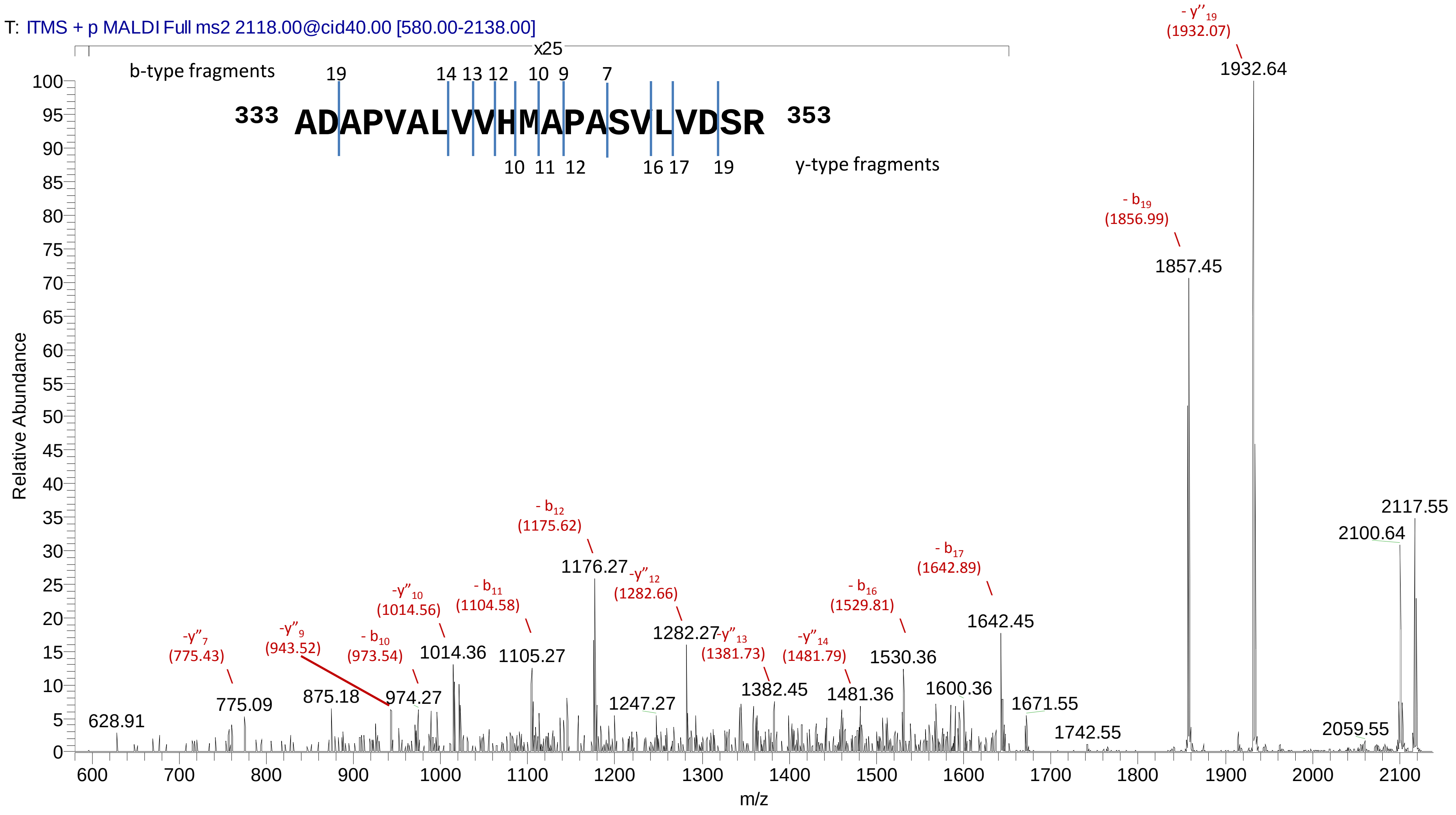

25. MS² m/z 2367

Theoretical mass: 2365.32 Da 385-404, *H. sapiens*

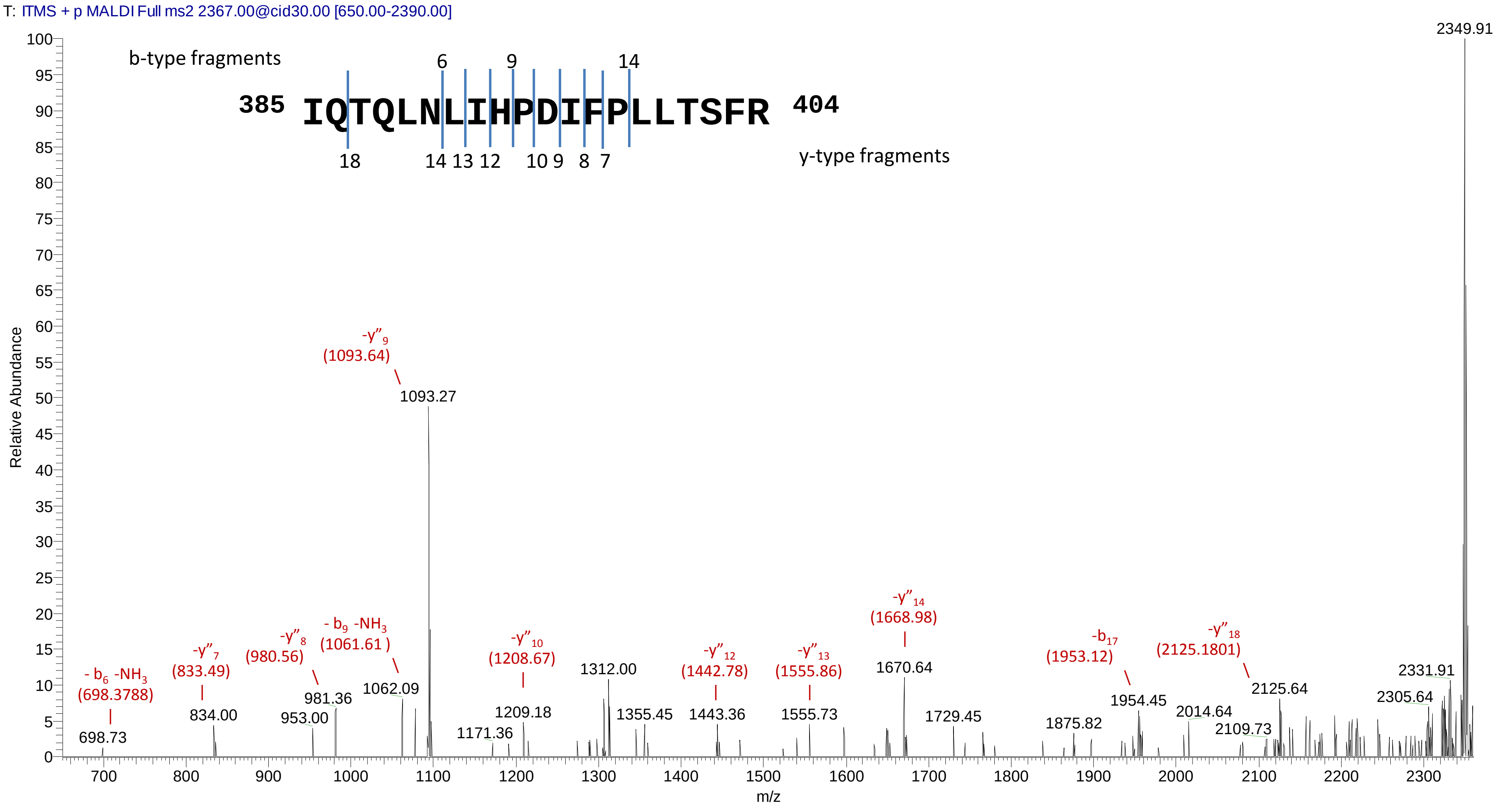

26. MS^2 m/z 2425

Theoretical mass: 2424.19 Da 679-700, *H. sapiens*

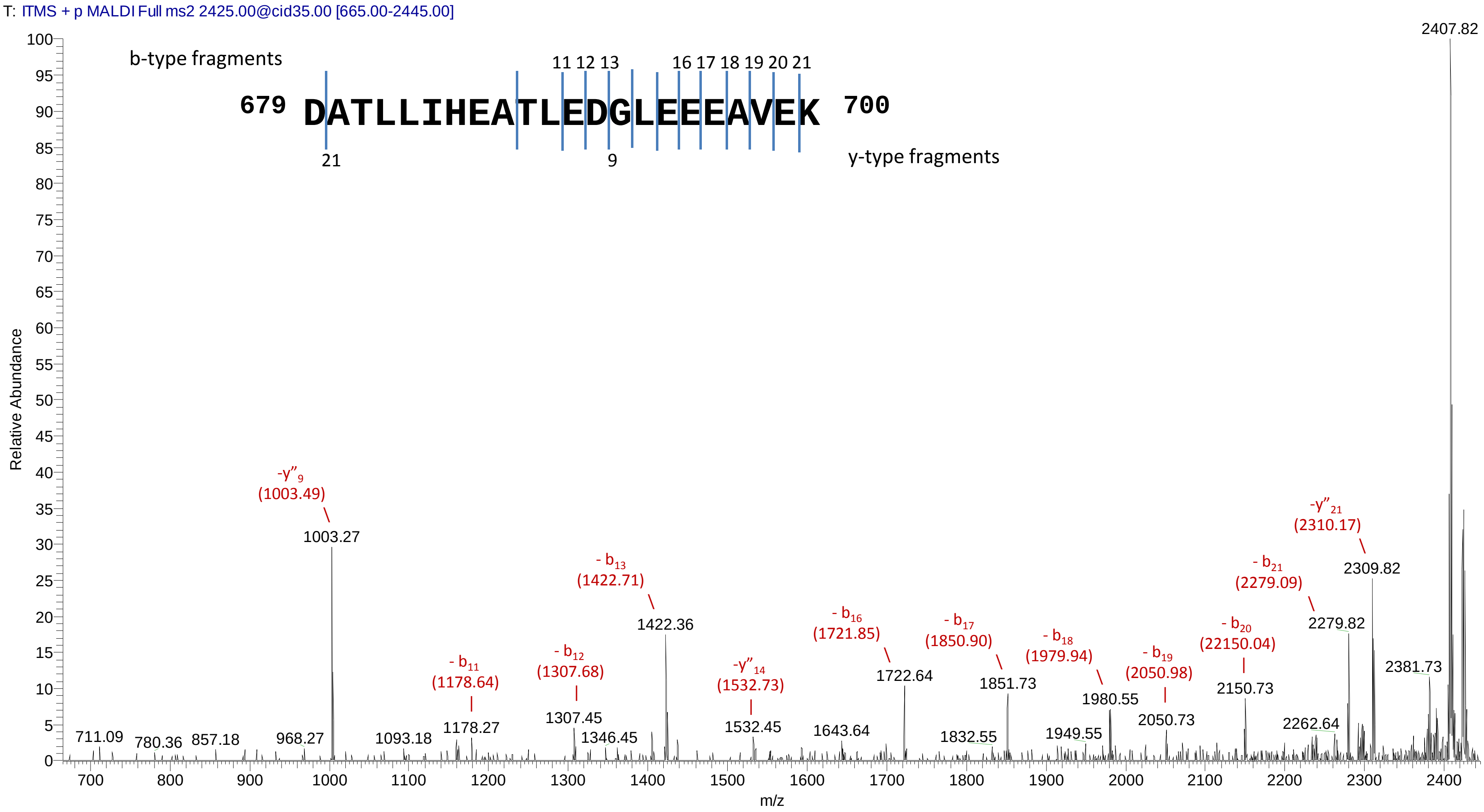

27. MS^2 m/z 2436

Theoretical mass: 2434.18 Da 361-381, *H. sapiens*

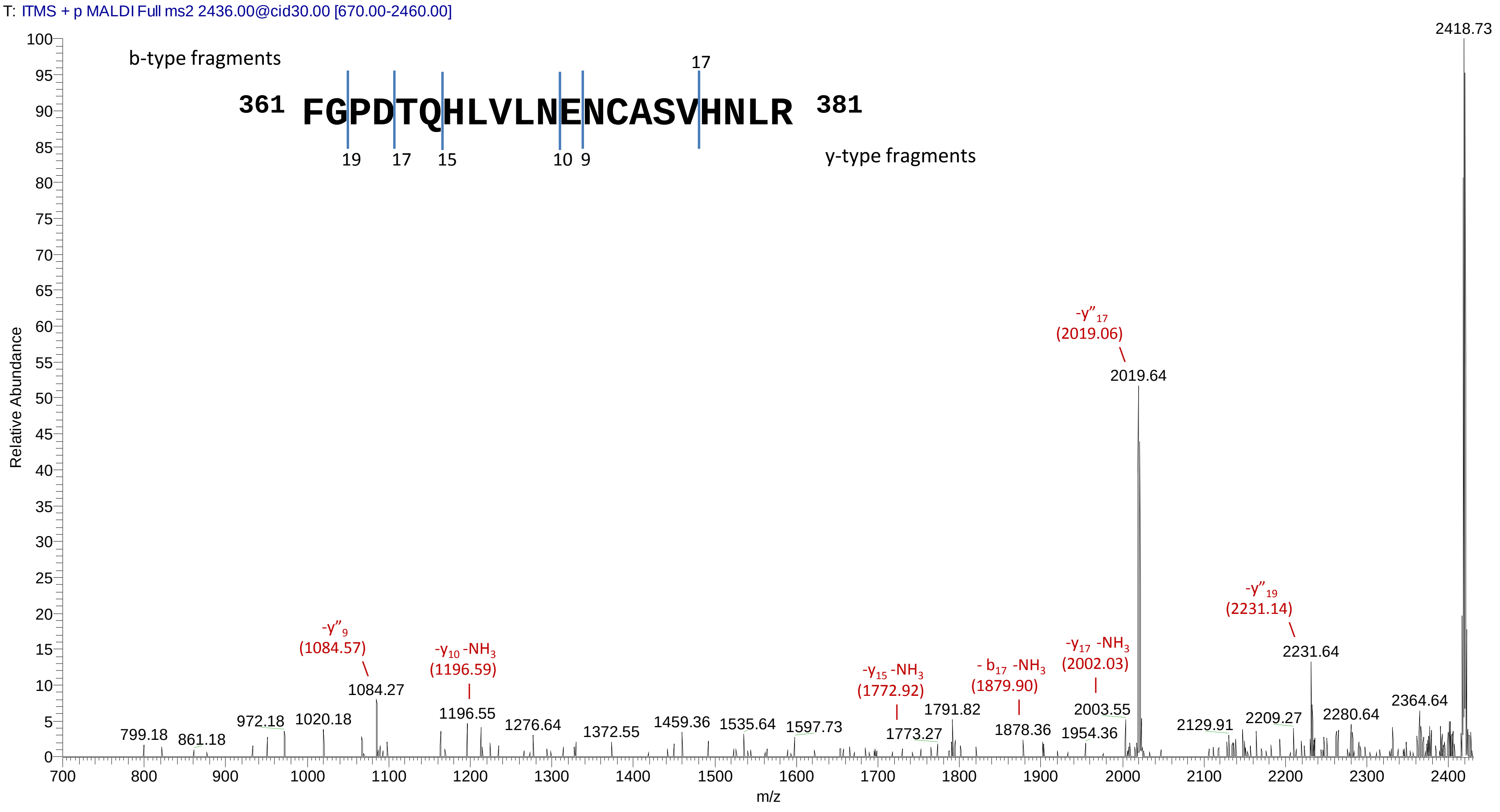

28. MS^2 m/z 2719

Theoretical mass: 2717.50 Da 382-404, *H. sapiens*

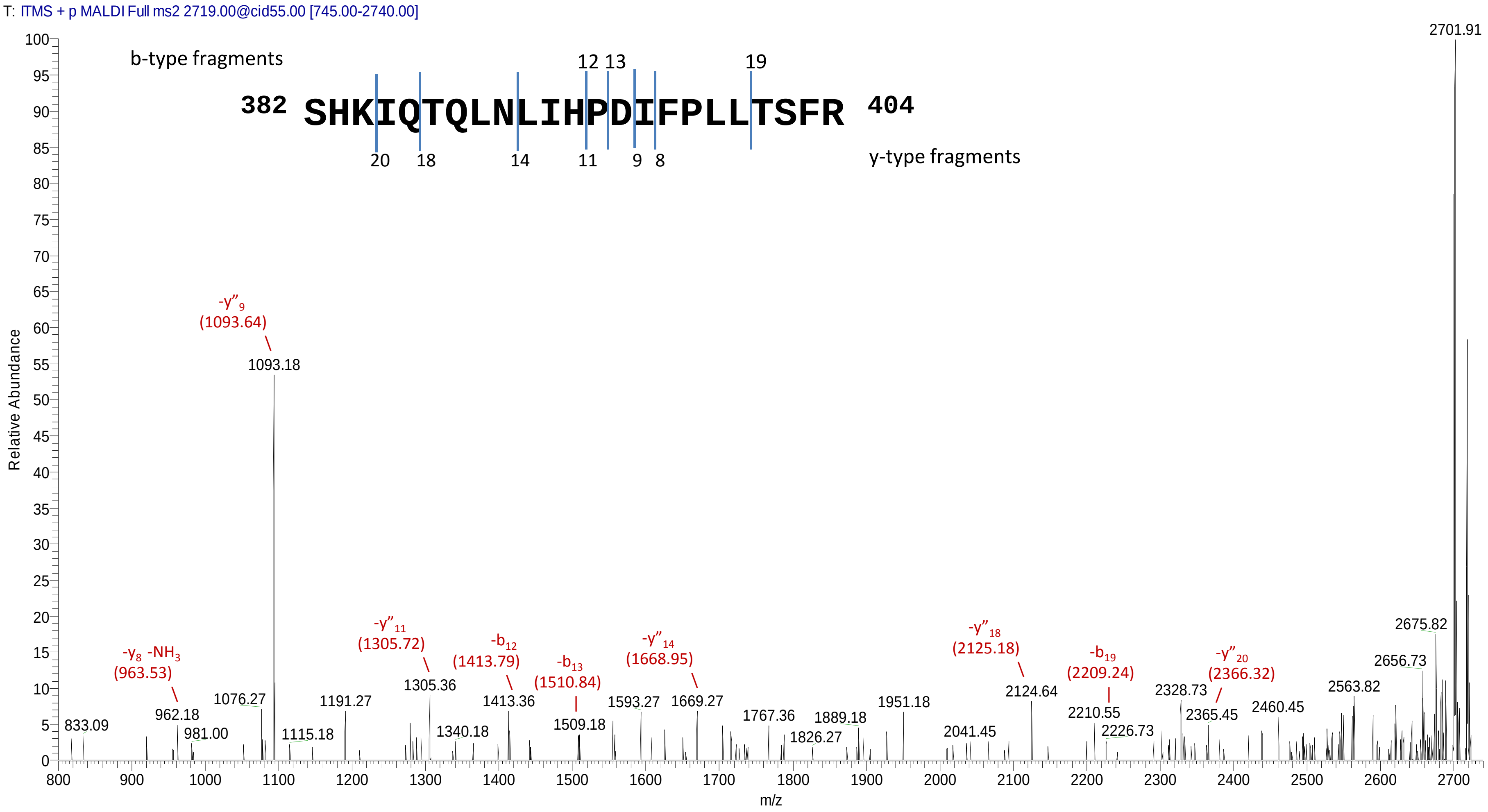

29. MS^2 m/z 2846

Theoretical mass: 2844.40 Da 586-608, *H. sapiens*

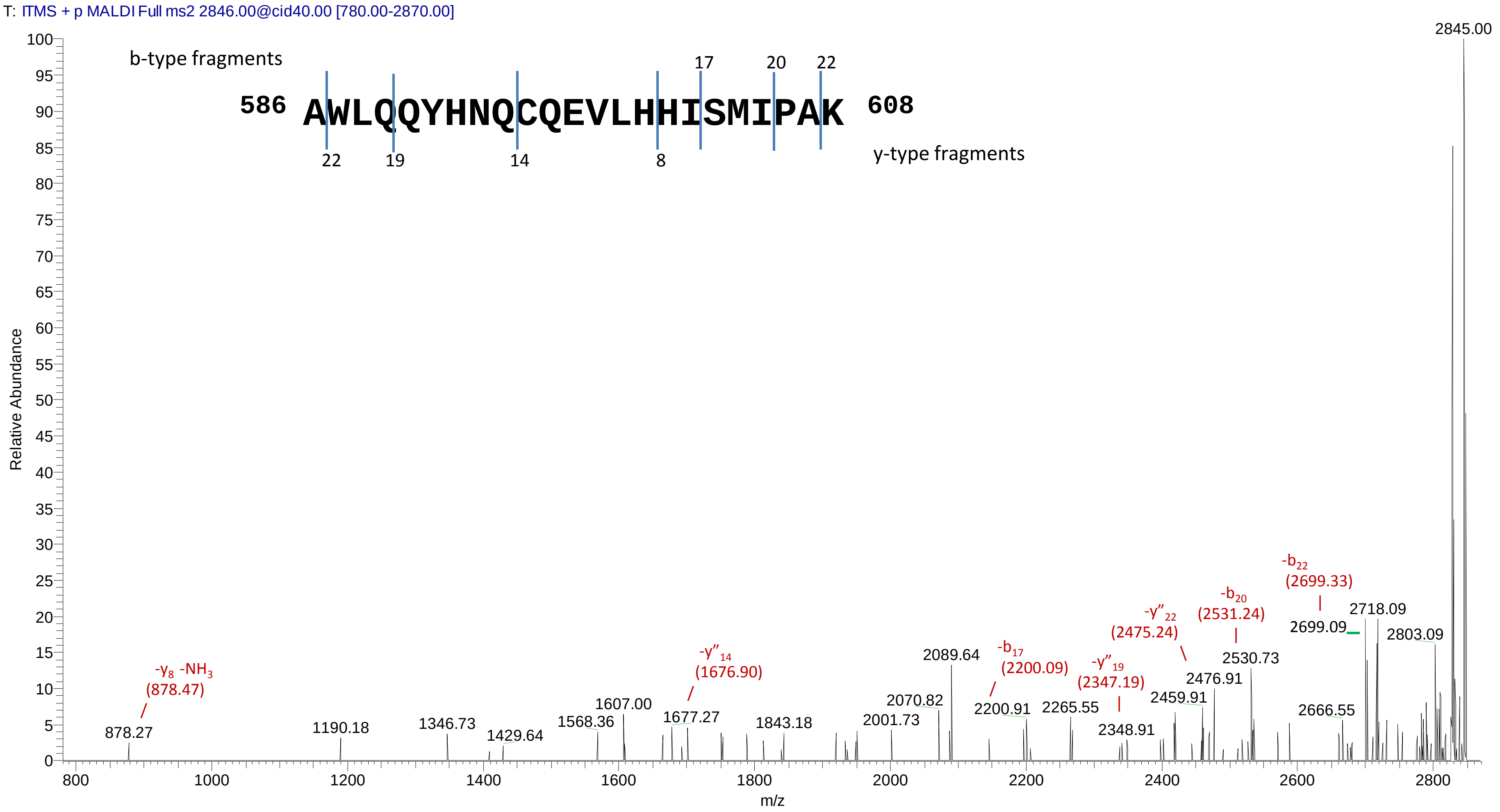

30. MS^2 m/z 3001

Theoretical mass: 3001.66 Da 535-562, *H. sapiens*

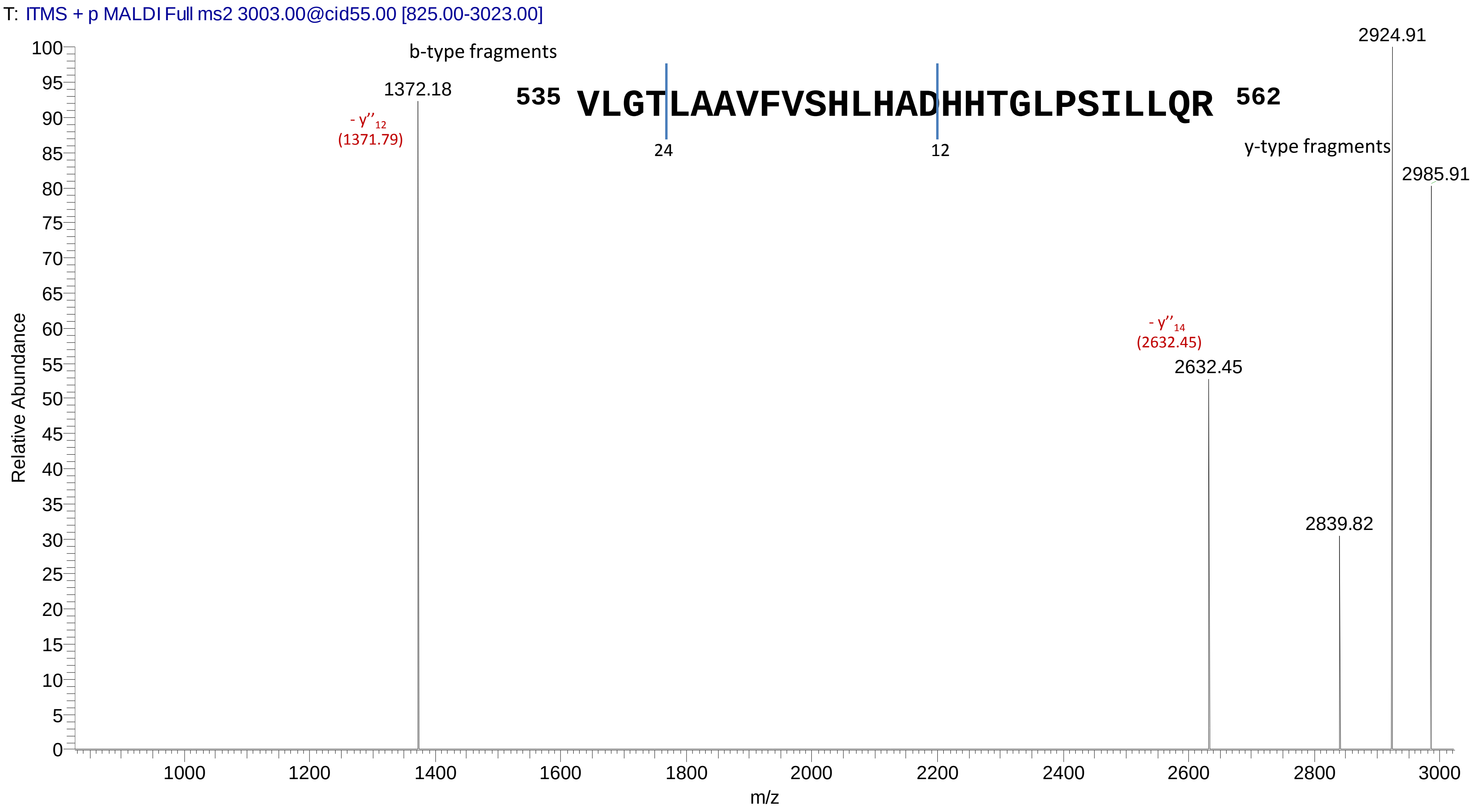

31. MS^2 m/z 3166

Theoretical mass: 3164.69 Da 498-526, *H. sapiens*

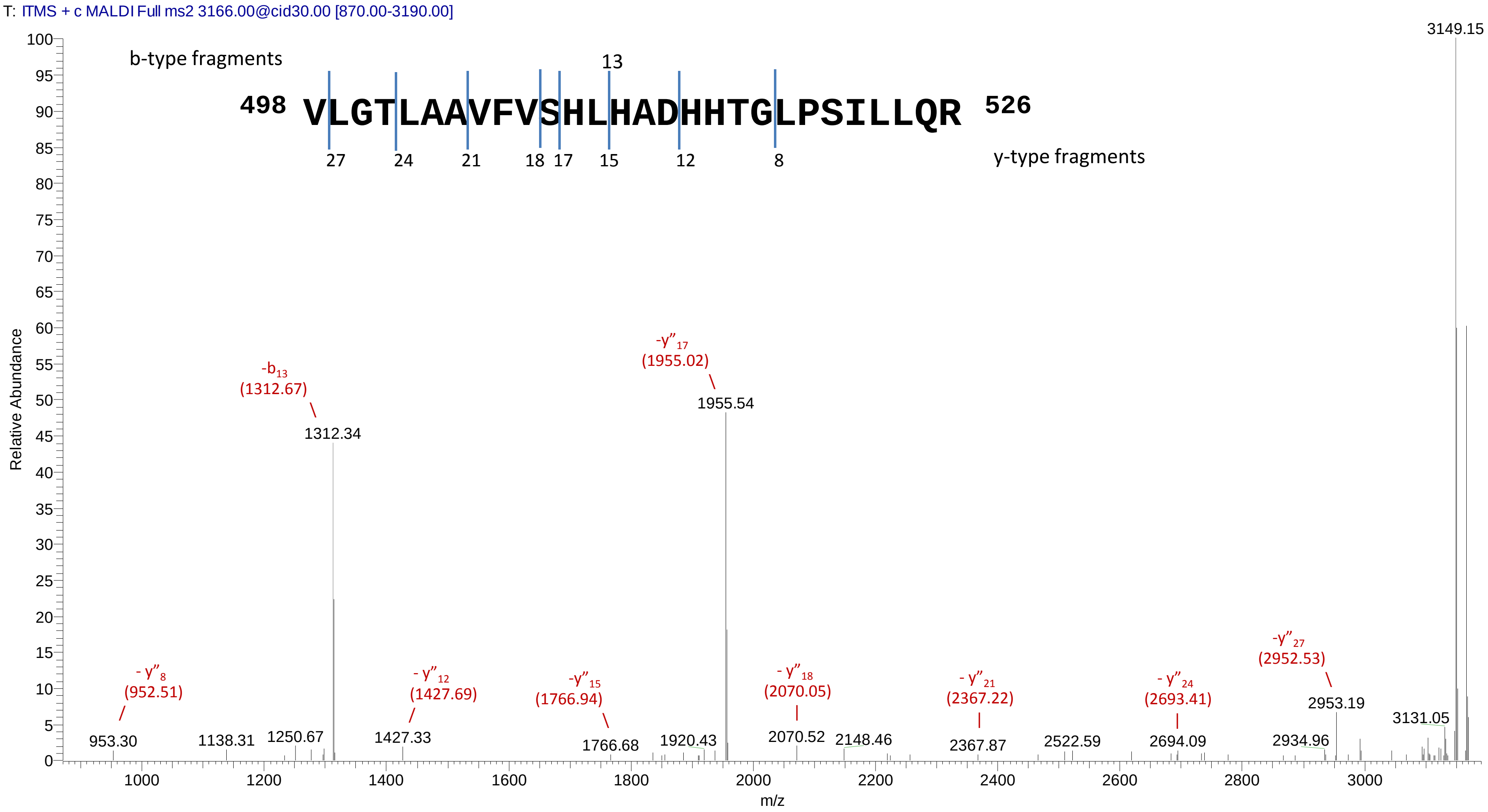

32. MS^2 m/z 3465

Theoretical mass: 3464.69 Da 436-464, *H. sapiens*

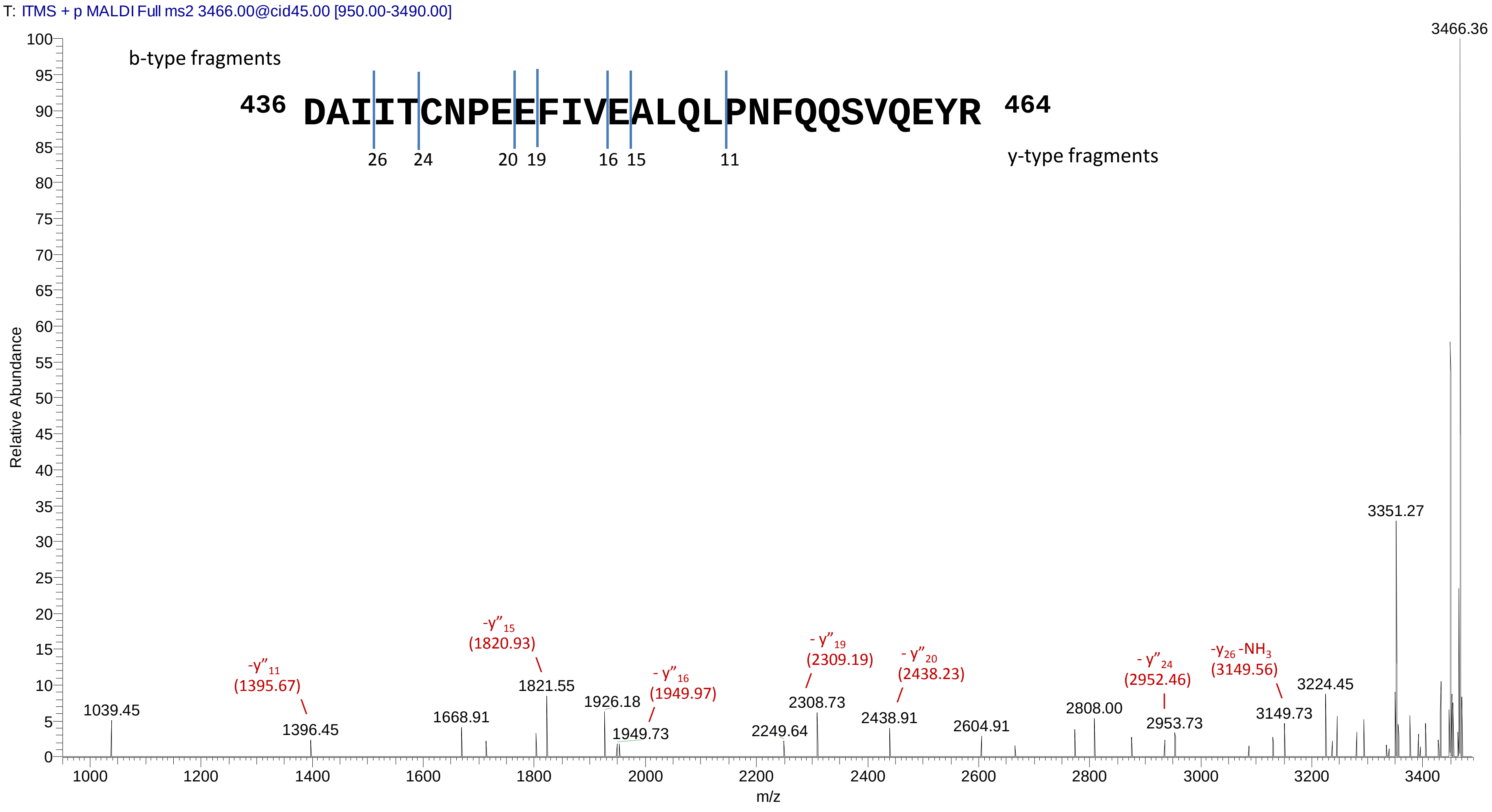

33. MS^2 m/z 3596

Theoretical mass: 3594.74 Da 160-190, *H. sapiens*

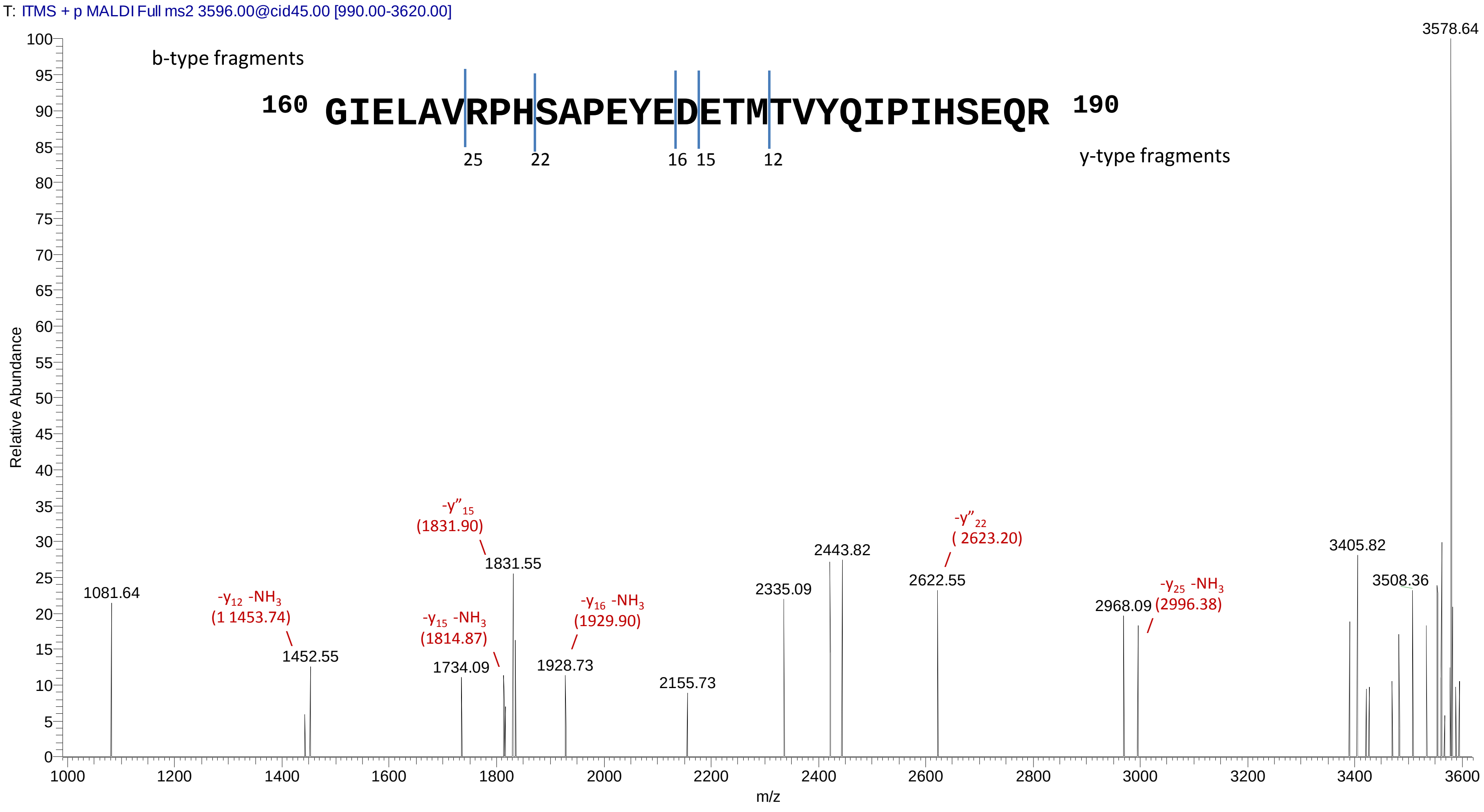

Supplement: Appendix S2 — MALDI-Ion trap MS/MS spectra from H. sapiens tRNAseZ. (PDF) [file pone.0066942.s002.pdf]
