# Supplementary material for: Tethered Domains and Flexible Regions in tRNase ZL, the Long Form of tRNase Z
Source: PLoS One. 2013 Jul 17;8(7):e66942. doi: 10.1371/journal.pone.0066942 (PMC3714273; doi:10.1371/journal.pone.0066942)
Supplement: File S1 — Contains supplemental figures SF1 – SF7, and supplemental tables ST1- ST9 and acc:ompanying text: Figure SF1. tRNA in a complex with tRNase ZS. Figure SF2. Full length tRNase ZL alignments. Table ST1. Wild Type D. melanogaster tRNase Z – Trypsin MALDI-ToF based on spectra in Figure 3B. Table ST2. Predicted members of D. melanogaster tRNase Z Cdom2 family. Figure SF3. D. melanogaster tRNase Z – Trypsin Full Length Coverage. Table ST3. Compilation of D. melanogaster tRNase Z peptide MALDI-Ion trap. Figure SF4. D. melanogaster tRNase Z ΔFA & L187A MALDI-ToF. Tables ST4, ST5. Polypeptide Tables based on spectra in Supplemental Figure SF4. Figure SF5. Detail views of tRNase Z FA free and bound to tRNA. Figure SF6. D. melanogaster tRNase Z– LysC & GluC MALDI-ToF. Tables ST6, ST7. Polypeptide Tables based on spectra in Supplemental Figure SF6. Table ST8. H. sapiens tRNase ZL – Trypsin MALDI-ToF Table. Figure SF7. H. sapiens tRNase ZL – Trypsin Full Length Coverage. Table ST9. Compilation of H. sapiens tRNase ZL peptide MALDI-Ion trap. (DOCX) [file pone.0066942.s003.docx]

Supplement to Wilson et al., 2013

Order of Supplemental figures, tables and appendices

Figure SF1. **tRNA in a complex with tRNase ZS**

Figure SF2. **Full length tRNase ZL alignments**

Table ST1. **Wild Type *D. melanogaster* tRNase Z - Trypsin MALDI-ToF based on spectra in Figure 3B**

Table ST2. **Predicted members of *D. melanogaster* tRNase Z Cdom2 family**

Figure SF3. ***D. melanogaster* tRNase Z – Trypsin Full Length Coverage**

Table ST3. **Compilation of *D. melanogaster* tRNase Z peptide MALDI-Ion trap**

Appendix 1. **MS/MS from Supplemental Table ST3 above**

Figure SF4. ***D. melanogaster* tRNase Z ΔFA & L187A MALDI-ToF**

Tables ST4, ST5. **Polypeptide Tables based on spectra in Supplemental Figure SF4**

Figure SF5. **Detail views of tRNase Z FA free and bound to tRNA**

Figure SF6. ***D. melanogaster* tRNase Z– LysC & GluC MALDI-ToF**

Tables ST6, ST7. **Polypeptide Tables based on spectra in Supplemental Figure SF6**

Table ST8. ***H. sapiens* tRNase ZL – Trypsin MALDI-ToF Table**

Figure SF7. ***H. sapiens* tRNase ZL – Trypsin Full Length Coverage**

Table ST9. **Compilation of *H. sapiens* tRNase ZL peptide MALDI-Ion trap**

Appendix 2. **MS/MS from Supplemental Table ST9**

**Motifs and other features of tRNase ZL**.The only available structures of tRNase Z complexed with tRNA are from *B. subtilis* tRNase ZS [1, 2]. Two conserved features of the complex, the active site of tRNase Z and interaction of the FA with the elbow of tRNA, are illustrated using the *B. subtilis* structure (Supplemental Figure SF1, adapted from PDB #2FK6).


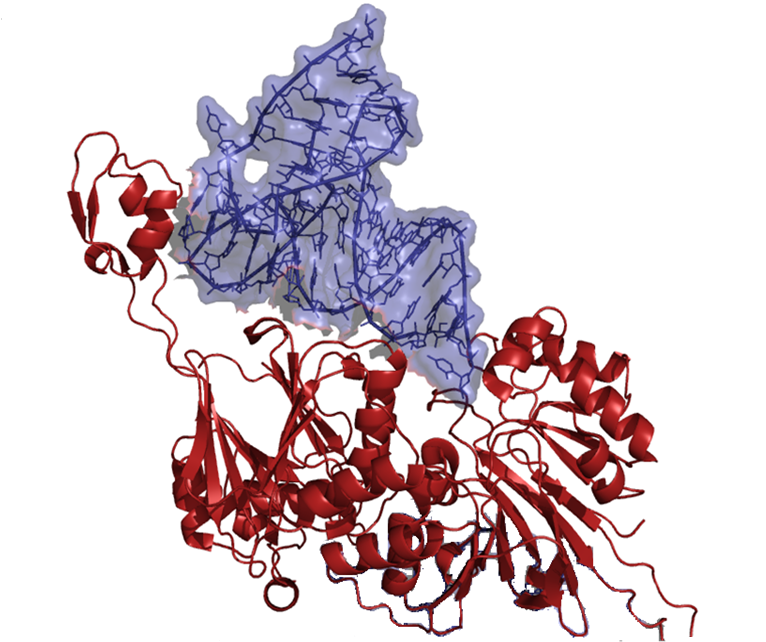


Active

Site

tRNA

Flexible

Arm

tRNase Z

Supplemental Figure 1. ***B. subtilis* tRNase Z complexed with tRNA** [1].

Other characteristics, including the greater size of the eukaryotic FA and additional differences throughout the amino domain, are so diverged from tRNase ZS that tRNase ZL structure cannot be deduced from the tRNase ZS structure. Intriguingly, a tether between the amino and carboxy domains of tRNase ZL is absent from tRNase ZS, a dimer of identical subunits. Structure of the tether and interactions between the amino and carboxy domains of tRNase ZL are thus unknown.

Flexible

Arm

Elements of *D. melanogaster* and *H. sapiens* tRNase ZL secondary structure assigned using PsiPred (a program designed for accurate protein secondary structure prediction; Bloomsbury Centre for Bioinformatics, University College-London) are similar to each other (Figures 4, 7), and also resemble the order, size and spacing of secondary structure elements in *B. subtilis* tRNase Z (a tRNase ZS consisting of 16 β strands interspersed with 9 α helices [1, 3]), compatible with the tandem duplication/adaptation explanation for genesis of tRNase ZL from tRNase ZS. Apart from the FA, secondary structure elements from *B. subtilis* tRNase Z can be accounted for in the carboxy domain of tRNase ZLs except that β16 close to the carboxy end is missing and a long α helix is present instead. The globular FA hand in *B. subtilis* tRNase Z, which consists of α4-GP loop-α5-β10-β11-η, is located between motifs III and IV (β9-β12). As previously described [4], tandem duplication/adaptation led to presence of the FA in the amino domain of tRNase ZL and its absence from the carboxy domain.

Annotated regions enclosed in dashed rectangles in the carboxy domain of tRNase ZL (Figures 4, 7 and Supplemental Figure SF2) were functionally characterized and previously described in detail ([5-7] and references therein); the description is extended here and in [9].

Supplemental Figure SF2. **Full length tRNase ZL alignments**. The long form of tRNase Z from *H. sapiens* (Hsa; Accession # [NP_060597](http://www.ncbi.nlm.nih.gov/protein/145553959)), *D. melanogaster* (Dme; [Q8MKW7](http://www.ncbi.nlm.nih.gov/protein/42559651)), *A. thaliana* (Ath; [AAM51378](http://www.ncbi.nlm.nih.gov/protein/21436181)), *C. elegans* (Cel; O4476) and *S. cereviseae* (Sce; NP013005.1) were aligned using Clustalw. Annotation is described in Supplemental Text.

Motif 2 (HxHxDH, the His cluster) contributes 4 of 7 residues to binding of two divalent metal ions and constitutes the architectural and catalytic core characteristic of the β-lactamase family of metal-dependent hydrolases. The motif 2 aspartate directs an OH- from a water molecule for nucleophilic attack on the scissile phosphodiester bond. The motif 3 and 5 histidines and motif 4 aspartate contribute additional coordination bonds for metal ion binding. The glutamate in the HEAT loop helps histidine of the HST loop to provide a proton to the oxyanion to complete catalysis. The motif 1 aspartate contributes to catalysis from outside the central catalytic core [8]. The PxKxRN loop, which contributes to the tRNase ZS dimer interface, has also been suggested to participate in function of CCA of mature tRNA as a tRNase Z antideterminant [4]. The recently discovered AxD motif [9] following motif 5 interacts with the PxKxRN loop.

The first three of the above functional elements (PxKxRN loop, motif 1, motif 2) have similar architectural elements in the amino domain of tRNase ZL (ψ-PxKxRN loop, ψ-motif 1, ψ-motif 2 [9, 10]). Although none of the functionally important amino acid residues remain, these structural relics may contribute to a related protein fold. The functional FA follows this structural beginning of the amino domain. While the FA is found in tRNase ZS between motifs 3 and 4, the FA in tRNase ZL follows ψ-motif 2 where ψ-motif 3 would have been expected. The tRNase ZL amino domain has the same pattern of predicted secondary structure elements as in *B. subtilis* tRNase Z up to and including the FA except that where β6-β9 are found in the *B. subtilis* structure, PsiPred predicts three β strands, not four.

After the FA, secondary structure elements can be assigned to the PsiPred tRNase ZL model up to β13 and α8. β14, 15 and 16 and α9 are not assigned; the tether is present instead [11]. After tandem duplication, the amino domain versions of motifs 3-5 apparently diverged.

The end of the carboxy domain is marked by a hydrophilic patch (enclosed in rectangles in Supplemental Figure SF2). Similarly, one or more hydrophilic patches mark the carboxy end of the amino domain (also enclosed in rectangles in the alignment). The last conserved sequence block in the amino domain proximal to tether (NdomTprox) is found on the amino side of these hydrophilic patches.

The boundary between the carboxy end of the tether and the start of the carboxy domain is marked by a conserved proline (***S*** in Figures 4 and 7; identical in 3/5 species in Supplemental Figure SF2 and in 19/26 species in [9]) in *H. sapiens* tRNase ZL and *D. melanogaster* tRNase Z about a dozen residues on the amino side of P in the PxKxRN loop. These conserved sequence features explain the position, boundaries and length of the tether but not its origin.

**Assignment of polypeptides to peaks in MALDI-TOF spectra** (**Supplemental Tables ST1, ST4-ST8)**. Peaks were assigned to polypeptides with the closest possible mass within 750 ppm (Supplemental Tables ST1, ST4-ST8) using both +1 and +2 (when there was a corresponding +1) ionization states. Some peaks are too heterogeneous to be assigned to a single proteolytic polypeptide due to spectral crowding and limited resolution; masses with multiple assignments are enclosed in rectangular borders. Assignments of observed masses to theoretical polypeptides that fit the model in Figure 11 are highlighted in yellow. Approximately 60% to nearly 100% of the assignments are consistent with that model. Theoretical pIs for the polypeptides are in the right-most column.

Some peaks fall into families which change systematically with time. In the spectra for proteolysis of wild type *D. melanogaster* tRNase Z by trypsin (Supplemental Table ST1), for example, Nflex cleavages can be nested in a basic patch (R25KKL), as can cleavages at the carboxy end of Ndom (K349, T350, L352) and at the Cend of Cdom (R736KRK). Trimming at the Cend often extends in the amino direction to the next cluster of basic residues (R726, K729, R730).

Yellow highlighting: polypeptides that fit into families described in text. Red type: peak masses interpretable as either Cdom or Ndom family. Cdom*, Cdom3*: these species were not observed in analysis of 2D gel spots.

Supplemental Table ST1 Polypeptide assignments for *D. melanogaster* tRNase Z – Trypsin MALDI-TOF Peaks based on spectra in Figure 3B

Yellow highlighting: polypeptides that fit into families described in text. Red type: peak masses interpretable as either Cdom or Ndom family. Cdom*, Cdom3*: these species were not observed in analysis of 2d gel spots.

Supplemental Table ST1 (continued)

Polypeptide assignments for *D. melanogaster* tRNase Z – Trypsin MALDI-TOF Peaks

A peak at 80,233 Da is observed in the 1 minute time point, in close agreement with the mass of the tryptic polypeptide formed after cleavage at R25↓ (calculated, 80,184 Da). Subsequent time points contain Nflex peaks that correspond to tryptic cleavage at all three residues in the N-terminal patch, as well as at K729, R730 and several other points in the C-terminal tail. K27-R730 (observed 78,454 Da, calculated 78,455 Da) is first seen at the 10 minute time point. Faint peaks corresponding to L28-R726 and L28-K729 are also observed.

A family of peaks in the 57-62 kDa range (FA region in Figure 3B; note 2.5x magnification on ordinate axis) arises from the cleavage of flexible basic residues in the globular FA hand combined with variable cleavage in the C-terminal tail (Supplemental Table ST1). A shift toward lower mass peaks occurs through the time course with progressive Cend trimming. The most prominent peaks (Figure 3B) correspond to cleavage at C192 and S222 in the FA hand. A peak at 60,612 Da, observed in the 15 minute time point, could arise from the fragment C192-R730 (calc. 60,609 Da), N208­-T743 (calc. 60,603 Da) or a mixture of the two. The latter polypeptide seems less likely because of progressive Cend trimming. C192 is observed at the amino end of other polypeptides while N208 is not. Faint, poorly-defined higher mass peaks at and above 63 kDa are consistent with cleavage at K171 and K181 in the ascending stalk of the flexible arm (for annotation of secondary structure, see Figure 4 and [18], cf [5, 7]), suggesting that the FA stalk is less flexible than the hand (Supplemental Figure SF5 and Discussion).

One mass peak in the 2 min spectrum was assigned to RA184-R730 (observed mass 61,738 Da; calculated mass 61,736 Da) among others may be plausible because the ascending stalk appears to flex nearby at G185 when the free protein is compared to tRNase Z bound to tRNA (Supplemental Figure SF5). The assignment of a single mass peak to multiple polypeptides can be resolved in most cases by reference to the theoretical isoelectric point and the results of 2D gel electrophoresis of the corresponding trypsin digestions (Figure 5). In this instance, with predicted pIs of 5.66, 5.95 and 6.34, relative spot intensities on the 2D gel favor the A184-R730 interpretation (pI 5.95).

Members of the Ndom and Cdom families (highlighted in red text in Supplemental Table ST1) are intriguing examples of data from MALDI-TOF spectra that do not distinguish between polypeptides with similar molecular weights. Two potential matches are observed for a peak at 44,790 Da in the 1 minute spectrum, K27 – K433 (an Ndom polypeptide, calc. 44,818 Da) and T350 – T743 (Cdom1, calc. 44,774 Da). A similar peak centered on 44,752 Da is observed in the 2 minute spectrum. Only one of the best assignments for this peak, T350 – T743, matches the assignment for the corresponding peak in the 1 minute spectrum. A shifted peak at 44,204 Da observed in the 5 minute spectrum matches T350-R738, with the same N-terminal end but a trimmed C-terminal end. The Ndom product can only be assigned from the earliest time point in this region of the spectra while the tether-Cdom assignments from T350 are supported through the time course. Such ambiguities are generally resolved by 2D gel analysis and subsequent mass spectrometry. In this instance the Ndom polypeptides with a carboxy end cleavage site at the far end of tether were not confirmed by peptide analysis of 2D gel spots, suggesting that they are rare and short-lived.

An Ndom fragment corresponding to cleavage at R25 in Nflex (R25KKL) and K349 (35,445 Da observed, 35,429 Da calc.) first appears at 1 minute and persists throughout the time course.The full Ndom­, from the N-terminus of tRNase Z to the 348-351 patch, with masses in the 38 kD region, was incompletely resolved from doubly protonated near-full length Nflex ions around 79 kD. A prominent band slightly smaller than 40 kD is clearly seen in the protein gel (Figure 3A), however, and a shoulder consistent with the fragment Nend-K348 (observed, ~38.8 kDa, calc, 38,787 Da), noticeable in the 5 minute time point, persists in an intense but heterogeneous, unresolved group of peaks in the 10 and 15 minute time points. A peak corresponding to an additional Ndom product, K26-K418 (43,208 Da observed, 43,213 Da calc; Supplemental Table ST1), would arise from cleavage close to the carboxy end of the tether. It may be present in the crowded ~43 kDa region of the spectrum from the 5 minute time point. A major flexible site in the tether, R384KG with variable Cend trimming, ranging in size from ~39 – 42 kDa, is largely obscured by the mass of +2Z and +2Z trimmed species. Prominence of this flexible region is substantiated by detailed analysis of the 2D gel spots (Figure 5) and of the ΔFA variant (Supplemental Figure SF3A and Supplemental Table ST4).

While bands and peaks are observed below 30 kD in the polyacrylamide gel and spectra (data not shown), smaller predicted products (e.g. Nend-Nflex, Nflex-FA or FA-tether) were not amenable to analysis; interpretation of smaller mass peaks in the spectra becomes impractical when they overlap with peaks due to multiple charge states, and artifacts would be due to progressive unfolding with secondary and tertiary cleavages.

**Predicted members of the *D. melanogaster* tRNase Z Cdom2 family**. The family observed on the 2D gel with the largest number of spots, designated Cdom2 in Figure 5, was modeled (Supplemental Table ST2) based on the results of limited proteolysis of *D. melanogaster* tRNase Z with trypsin and MALDI-ToF mass spectrometry (Figure 3), the relative molecular weights and relative pIs from the 2D gel separation (Figure 5) and theoretical pIs (rightmost column in Supplemental Table ST1). Combinatorial cleavage is presented for two contiguous basic residues in the tether (R384KG) and at two clusters of basic residues close to the carboxy end, (K729RELK)QE(R736KRK)LAET743. The table thus includes 16 predicted polypeptides, enough to account for the ~10 spots observed in the Cdom2 family on the 2D gel. Predicted molecular weight decreases with pI. Predicted mass for the family ranges from 38,964-40,860 Da and theoretical pI ranges from 5.55-5.99. Consistent with this interpretation of the Cdom2 family, peaks in the MALDI-TOF spectra can be assigned to five of the polypeptides (Supplemental Table ST1, highlighted in yellow in Supplemental Table ST2).

Supplemental Table ST2

Predicted members of *D. melanogaster* tRNase Z Cdom2 family

**Coverage of full length *D. melanogaster* tRNase Z and** **compilation of *D. melanogaster* tRNase Z peptide MALDI-Trap**. Full length *D. melanogaster* tRNase Z spots from 2D gels (Figure 5) were manually excised and trypsinized; resulting peptides were analyzed using MALDI-ion trap single stage and tandem mass spectrometry. Peptides detected from the exhaustive tryptic digestion are highlighted in yellow (Supplemental Figure SF3). Coverage is approximately 78%, and most of the missing peptides are either too small or too large for the methodology.

Complete peptide results from the families of spots identified in Figure 5 are compiled in Supplemental Table ST3; sequences are shown using one-letter amino acid abbreviations and peptides detected are indicated by their first and last residue numbers. N indicates the N-terminus of the recombinant protein after removal of the His-tag using TEV protease, and includes a non-genomically encoded 7 residue leader. All but one of the peptides (marked with an asterisk) were manually confirmed by MS2 (Supplemental Appendix 1).

Limited proteolysis fragments fall into major categories designated in columns in Supplemental Tables ST3 and ST8 as in the rows of Tables 2 and 3. Color coding is consistent with that in Figure 10: the blue background indicates tryptic peptides that arise from the N-terminal domain, with the flexible arm emphasized in light blue, polypeptides from tether in grey and polypeptides from the C-terminal domain in red.

The presence of a particular peptide within representative spectra for a gel spot is noted with ‘Y’ (Yes) in Supplemental Table ST3. Boundaries are established by tracking peaks that are absent from the spectrum of a polypeptide which are present in spectra from higher molecular weight or full length species (given a sufficient mass spectral response when present). This information is combined with relative pI from the first (IEF) dimension and relative molecular weight from the second (SDS-PAGE) dimension of the 2D gels (Figure 5, Supplemental Tables ST1 and ST2) to identify the source polypeptide, and further supported by MALDI-TOF spectra of the limited proteolysis mixtures (Figure 3 and Supplemental Table ST1). For example, complementation between Ndom and Cdom species (Supplemental Table ST3) shows that the amino and carboxy domains are most prominently demarcated by flexible regions within tether (starting with R384) and at the amino end of tether (starting with K348).

Supplemental Table ST3 ***D. melanogaster* tRNase Z gel spots by MS/MS**

Supplemental Figure SF3 ***D. melanogaster* – Trypsin tRNase Z Full Length Coverage**

**MS/MS spectra**. Limited proteolysis products of recombinant wild-type *D. melanogaster* tRNase Z and wild-type *H. sapiens* tRNase ZL separated by 2D electrophoresis were exhaustively proteolyzed with trypsin by in-gel treatment and subjected to MALDI-ion trap collisionally induced dissociation tandem mass spectrometry. Representative spectra are collated in Supplemental Appendices 1 and 2, respectively. The only post-translational modification observed was propanamide modification of cysteines arising from *in situ* reaction with acrylamide monomer under IEF conditions. Peptide 542 – 570 from *D. melanogaster* tRNase Z is observed both with and without cyclization of the N-terminal Gln residue to pyrrolidone carboxylic acid (pyroQ), confirmed by MS2 (Supplemental Appendix 1). Non-stoichiometric methionine oxidation which may have occurred *in situ* is also occasionally observed (spectra not shown).


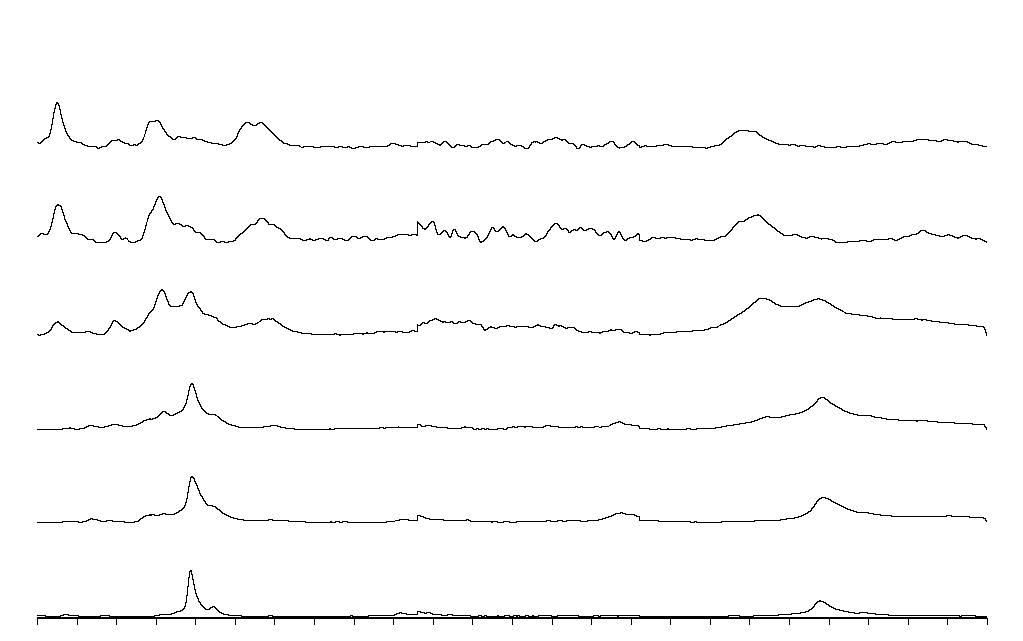


0

min

1

2

5

10

15

Ndom, Cdom

Supplemental Figure SF4A

Nflex/C end

*D. melanogaster* ΔFA-Trypsin

30

40

50

60

70

80

90k

35

45

55

75

65

85

m/z

+1 Z

79,420

74,174

\

44,154

/

34,811

\

31,245

/

+2 Z

75,752

/

44,247

/

43,293

\

34,936

\

35,120

\

34,960

/


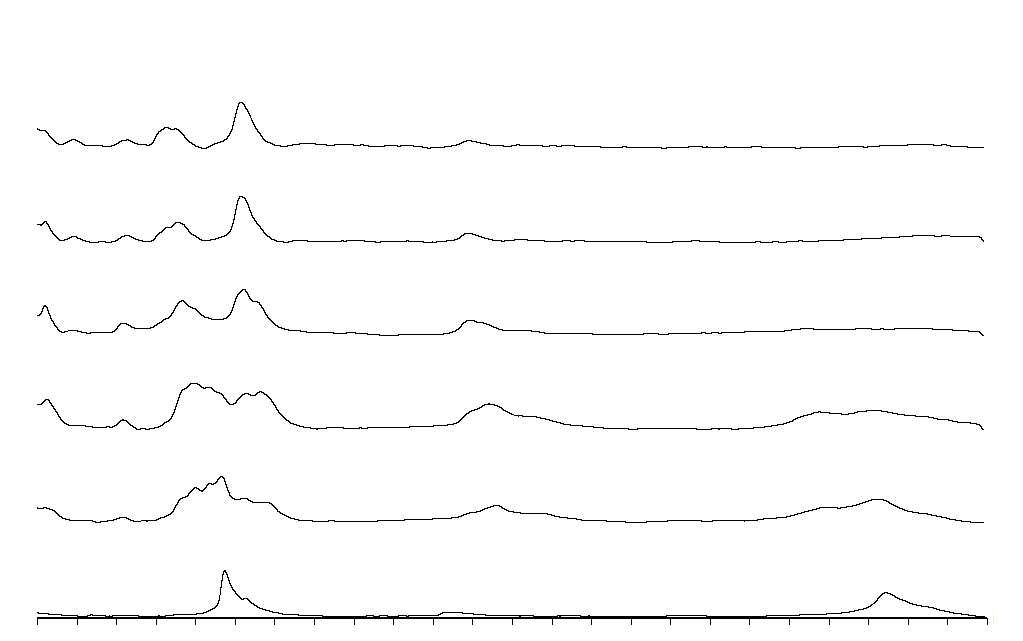


Supplemental Figure SF4B

30

40

50

60

70

80

90k

35

45

55

75

65

85

m/z

0

min

1

2

5

10

15

FA/C end

83,670

Nflex/C end

+2 Z

+1 Z

79,322

61,826

60,602

58,995

58,514

57,371

44,066

43,058

35,698

38,172

*D. melanogaster* L187A-Trypsin

\

/

\

/

/

/

/

/

\

/

43,178

\

35,746

\

35,385

\

32,201

/

Ndom, Cdom

Certain tryptic peptides (for example, D10-R16), particularly in the lower molecular weight region where MALDI spectra are commonly cluttered with matrix-ion cluster peaks and other background, are observed only in spectra from more intense spots. Absence of these peaks from the spectra of less intense spots does not substantially detract from the overall interpretation of domain structure and protease-accessible regions. In the case of polypeptides in the *D. melanogaster* Ndom1 region (Figure 5, Table 2, Supplemental Table ST3), for example, the presence of D10-R16 cannot be ruled out, therefore the Y17-R25 boundary peptide at the amino end is not firm. Indeed, a match to D10-K402 is found in the 1D MALDI-TOF with observed mass of 43,208 and calculated mass of 43,225 Da.

**FA variants of *D. melanogaster* tRNase Z**. Two variants of *D. melanogaster*tRNase Z, deletion of the globular hand of the flexible arm (ΔFA; see Figure 4 and Supplemental Figure SF2 for annotation) and substitution of a single leucine with alanine at the ascending stalk/hand boundary (L187A, a high *K*M variant) were analyzed by limited proteolysis with trypsin and mass spectrometry as in Figure 3B (Supplemental Figure SF4; Supplemental Tables ST4, ST5). ΔFA tRNase Z is the 42 residue deletion described previously [7] (Δ183/226 retains R183 and T226 with 42 residues deleted from A184 to V225).

Supplemental Table ST4, ST5. Polypeptide assignments for *D. melanogaster* tRNase Z

ΔFA and L187A – Trypsin MALDI-ToF Peaks based on spectra in Supplemental Figure SF4

Consistent with the model presented in Figure 10, ΔFA tRNase Z retains cleavages similar to wild type in the tether, Nflex and Cend regions. L187A tRNase Z is more rapidly digested with trypsin than wild type tRNase Z, and the FA is especially susceptible.

A stable form of *D. melanogaster* tRNase Z with the flexible arm deleted (ΔFA) displays close to a 100-fold increased *K*M for pre-tRNA processing with practically no effect on *k*cat [7], demonstrating that the FA functions principally in pre-tRNA binding, as previously suggested [1, 3, 4, 13, 14]. ΔFA tRNase Z is 4,264 D smaller than full length tRNase Z (Δ183/226 retains R183 and T226 with 42 residues deleted from A184 to V225; see Figure 4 for annotation). Purified ΔFA tRNase Z was treated with trypsin as with wild type tRNase Z. The measured mass of ΔFA is 79,420 Da (calc., 79,407 Da) and three of the four main flexible regions, excluding the deleted FA, are observed essentially as in wild type (Supplemental Figure SF4A and Supplemental Table ST4; numbering of residues is the same as for wild type tRNase Z). Prominent peaks correspond to tryptic fragments produced from cleavage in the Nflex basic patch (R25KKL) and in the 348-351 patch at the Ndom-tether boundary, combined with variable Cend cleavage. FA site cleavages in wild-type tRNase Z that give rise to peaks in the 60 kDa region of the spectra are, as expected, missing from ΔFA tRNase Z (Supplemental Figure SF4A, magnified region). The similarity of flexible regions (except in the FA) in wild type and ΔFA tRNase Z supports a model of the FA as an independently folding domain.

Based on this reasoning, the 43/44 kDa peaks in wild type tRNase Z are mainly Cdom, since the same peaks are prominently observed in ΔFA tRNase Z, with the advantage that reduced mass of the +2 region lessens clutter around 44 kDa.  Although the 44,154 Da peak in the ΔFA 15 min spectrum can be interpreted as N-K433 (an Ndom polypeptide), a corresponding predicted 48,431 Da peak was not observed in the spectra with wild type tRNase Z. The 35,416 Da (2 min), 35,506 Da (5 min), 35,416 Da (10 min) and 35,508 Da (15 min) peaks in the wild type tRNase Z spectra (Figure 3B, Supplemental Table ST1) mainly come from Ndom since there is a corresponding ΔFA Ndom peak at 31,245 Da (Supplemental Figure SF3A; Supplemental Table ST4), the mass difference being due to the deleted FA residues (4,282 Da reduced mass). ΔFA Ndom peaks at 34,811 Da (2 min), 34,936 Da (5 min) and 34,960 Da (15 min) interpreted as arising mainly from cleavages at R384 and K385 correspond to the 39 kDa peaks in wild type tRNase Z (Supplemental Table ST1).

L187A, a substitution at the boundary between the ascending stalk and α4 of the *D. melanogaster* tRNase Z FA hand (terminology is from [7, cf 1]; for annotation, see Figure 4), causes *K*M for pre-tRNA processing to increase almost 100-fold with little effect on *k*cat7, similar to deletion of the entire FA hand. Replacing this bulky hydrophobic residue apparently renders the FA virtually invisible to substrate. It was therefore of interest to determine the effect of the L187A substitution on flexibility of tRNase Z.

The L187A variant of tRNase Z was subjected to limited proteolysis with trypsin (Supplemental Figure SF4B and Supplemental Table ST5). Qualitatively, L187A cleavages are similar to those observed with wild type tRNase Z, including cleavage at the Nflex patch, in the FA at K191, K207, and K221, the 348-351 patch adjacent to the tether, and in the C-terminal tail. Cleavage at K418 toward the carboxy end of the tether, regarded as a possible site in wild type and ΔFA tRNase Z, is clearly observed in L187A.

The rate of appearance and persistence of individual peaks in the time course are strikingly different in wild type tRNase Z and the L187A variant. First, the full length and near-full length Nflex peaks larger than 78 kDa disappear more rapidly for L187A than for wild type; for wild type tRNase Z, these families of peaks dominate the spectra at 2 minutes, and are still clearly present at 15 minutes, whereas for L187A they are less intense than FA and Cdom peaks, and virtually undetectable by 5 minutes. Second, for wild type tRNase Z, the FA peaks are barely noticeable at 1 minute (even with 2.5x magnification of peak amplitude in this region), then increase in intensity and persist until 15 minutes; for L187A, FA peaks are clearly present at 1 minute without magnification, then quickly decrease in relative intensity from 5 minutes onward. Third, for wild type tRNase Z, the major Ndom3 peak at 35.4 kDa is more substantial and homogeneous than the corresponding peak in L187A.

In summary, the L187A variant is proteolyzed faster overall than wild type tRNase Z. Increasing the rate of cleavage in FA depletes the yield of Ndom polypeptides, and FA cleavage combined with cleavage in tether would leave Cdom as the only stable polypeptide. Nevertheless, stability of L187A tRNase Z to intracellular proteolysis [7] as well as cleavage results that qualitatively fit the model (Figure 10) suggest that the L187A variant retains a stable fold.

**Movement of the FA could be limited by structure of the FA stalk**. Robust cleavage at K207 in the L187A variant starting from the 1 minute timepoint, which is seldom if ever seen in wild type tRNase Z, and only late in the time course, is another difference between L187A and wild type *D. melanogaster* tRNase Z (Supplemental Figure SF4B, cf Figure 3A, Supplemental Tables ST1, ST5). Endoproteinase Lys-C also does not cleave K207 in wild type tRNase Z (Supplemental Figure SF6A). This residue is conserved, present in 3 out of 5 species in the alignments (Supplemental Figure SF2) and at the same position in *B. subtilis* and *E. coli* tRNase ZS [3,4], and in 19 of 26 species in the metazoan tRNase ZL alignments [9]. K207 may function in conformational changes associated with tRNA binding (Supplemental Figure SF5).

Supplemental Figure SF5. **Structure of *B. subtilis* tRNase Z free and bound to tRNA** (from [1, 2]). A) Blue and red: free and bound tRNase Z, respectively [1, 2] displayed using PyMol [15]. tRNA is not shown. B) The FA is enlarged, including the structured stalk and globular hand. A flexion angle of ~18o is obtained between the two structures with the vertex on the amino side of G185 in the ascending stalk. C) The FA of *B. subtilis* tRNase Z free protein [2]. The hand is shown in Ribbon and the stalk is in ball-and-stick. ↑,↓: ascending and descending strands of the stalk, respectively. Dashed lines: the stalk may be stabilized by a network of H-bonds. Dashed circle encloses the electrostatic bond between the ε-NH2 group of K207 and the backbone carbonyl of G185. D) The tRNase Z FA and elbow of the tRNA from the *B. subtilis* co-crystal structure [1] displayed in Ribbon. The U55, C56 bases are shown in ball-and-stick. K207 is electrostatically bound to the PO4- between U55 and C56 (dashed circle). Viewing angle between (C) and (D) was adjusted to best view the respective K207 electrostatic bonds. (E), (F) illustrate dihedral angles in the K207 side chain in the *B. subtilis* protein-only and co-crystal structures.

The structure offree *B. subtilis* tRNase Z (PDB #1Y44 [3]) is presented in blue and the superimposed protein bound to tRNA (PDB #2FK6 [1]) is in red (Figure SF5A). The enlarged view (Supplemental Figure SF5B) illustrates that the FA moves away from the active site when tRNA is bound (Supplemental Figure SF5A, B; cf [1, 3]) by an 18o angle measured from the vertex on the amino side of G185 in the ascending strand (Supplemental Figure SF5B) where the stalk appears to flex.

The crystal structure of *B. subtilis* tRNase Zs shows the FA hand connected to the body of the protein via a stalk consisting of two antiparallel zigzag polypeptide strands designated neither helix nor sheet (Supplemental Figure SF5C, cf [3]). A network of backbone hydrogen bonds can be modeled between ascending and descending strands of the *B. subtilis* FA stalk (Supplemental Figure SF5C; cf [1, 3]), suggesting that it is relatively inflexible. The same type of cross-strand H-bond network in the FA stalk of *D. melanogaster* tRNase Z (Psipred assigns β structure to both ascending and descending strands of the tRNase ZL FA stalk; Figures 4, 7) would explain why the FA stalk is generally less flexible than the hand. Measurements of Cα bond lengths and angles in the *B. subtilis* and *E. coli* FA stalks and β strands are similar (not shown); FA stalks were probably not designated as β strands [3, 13] because proline and glycine residues, incompatible with classical elements of secondary structure, are present. The *T. maritima* tRNase Z FA stalk consists of long antiparallel β-strands with no Pro or Gly residues [14].

K207 is located in α5 (secondary structure designations in Supplemental Figure SF5C are from the *B. subtilis* tRNase Z structure [2, cf 1]), with its ε-amino group positioned within electrostatic bonding distance of the backbone carbonyl group of G185 in the ascending stalk (circled in Supplemental Figure SF5C). In the structure of tRNAse Z bound to tRNA, however,

this bond is replaced by a protein-tRNA contact (Supplemental Figure SF4D, cf C) due to rotation of the ascending stalk and α4 away from the body of tRNase Z (α4 and η rotate away from the active site by up to 5 Ǻ in the co-crystal structure compared to the protein-only structure [1-3]). The K207 side chain rotates away from the stalk to contact tRNA; the dihedral angle between the γ and δ carbons in the K207 side chain changes from the anti-periplanar (-180o) to the gauche (-60o) configuration (panels E, F in Supplemental Figure SF4). Correspondingly, the distance from the ε-NH2 group of K207 to the G185 backbone carbonyl increases to 4.6 Ǻ and is replaced by the 3.4 Ǻ contact between K207 and the 5’-PO4- of nt C56 in the T loop (D in Supplemental Figure SF5).

K207 could organize FA structure and control dynamics of the transition to tRNA-bound tRNase Z by setting the angle between the closed FA hand and stalk in free tRNase Z and opening the structure for substrate binding. K207 becomes accessible in the L187A high KM variant; loss of its ability to bind tRNA correlates with the increase in flexibility which may change the ability of K207 to form appropriate noncovalent bonds. In the co-crystal structure of *B. subtilis* tRNase Z with tRNA [1], a hydrophobic van der Waal’s and stacking contact is observed between L187 at the ascending stalk-FA hand boundary (L158 in the *B. subtilis* sequence) and C56 in the T-loop of tRNA. The L187A mutation, perhaps because the smaller alanine side chain does not provide sufficient bulk for a robust hydrophobic core in the globular, already flexible hand, appears to structurally destabilize the FA hand so as to render it invisible to substrate.

An inverse correlation between FA flexibility and catalytic efficiency is observed between the wild type and L187A variant of *D. melanogaster* tRNase Z (Supplemental Figure SF4B, cf Figure 3B). Increased FA flexibility is clearly seen in L187A relative to wild type *D. melanogaster* tRNase Z; fragments corresponding to FA cleavage appear early and disappear quickly, suggesting faster initial cleavage in the FA hand, followed by rapid secondary cleavage to Cdom and low molecular weight peptides. L187A tRNase Z has a 1-2 order of magnitude lower catalytic efficiency than wild type tRNase Z due to increased *K*M.

In a related instance, *H. sapiens* tRNase ZL appears to have a less flexible FA hand relative to other sites than *D. melanogaster* tRNaze Z; FA peaks appear later than tether peaks for *H. sapiens* tRNase ZL, but at about the same time for *D. melanogaster* tRNase Z. *H. sapiens* tRNase ZL has between 1-2 orders of magnitude higher catalytic efficiency than *D. melanogaster* tRNase Z (from comparisons between [4, 5, 13, 14]). A too flexible FA can thus be catastrophic for catalysis (*D. melanogaster* L187A vs wild type tRNase Z) and a less flexible FA is associated with higher catalytic efficiency (*H. sapiens* tRNase ZL vs *D. melanogaster* tRNase Z). A less flexible FA thus correlates with higher catalytic efficiency, although other differences could contribute to the greater catalytic efficiency of *H. sapiens* tRNase ZL.

**Limited proteolysis with alternative enzymes.** In addition to trypsin, wild type *D. melanogaster* tRNase Z was subjected to limited proteolysis time courses with endoproteinases LysC and GluC (Supplemental Figure SF6). LysC cleaves on the carboxy side of lysine but not arginine, and GluC cleaves on the carboxy side of glutamate. The three enzymes interrogate a chemical space that is both overlapping (LysC/trypsin) and orthogonal (GluC).

Supplemental Figure SF6. **Endo LysC and GluC cleavages support the trypsin assignments**. A) Details are the same as in Figure 3B except that *D. melanogaster* tRNase Z was digested with LysC instead of trypsin. Reduced heterogeneity in the spectra is due to the absence of arginine cleavages. The complete table is presented in Supplementary Table T3. B) GluC, which cleaves on the C-side of flexible glutamates, was used instead of trypsin. There are 57 glutamates in *D. melanogaster* tRNase Z. The complete table is presented in Supplementary Table ST4.

LysC proteolytic products are similar to those observed with trypsin, supporting the overall interpretation (Figure 10). Major protease accessible regions are still observed at lysines in the Nflex and 348-351 basic patches as well as in the FA hand and the C-terminal tail; LysC also cleaves at various points in tether (Supplemental Figure SF6A). There are three noteworthy differences between the LysC and trypsin experiments. First, the near full-length Nflex fragment around 78 kDa observed in the trypsin time course (produced by trypsin cleavage at the Nflex patch and at R730, which is not sampled by LysC) is instead seen at 80,039 Da (K27-T743; MW calc 80,056 Da). LysC cleavage at K729 is observed in smaller fragments. Second, differences are observed in the FA region. The most prominent site seems to be K191, which coincides with a prominent trypsin-accessible FA cleavage. An additional cleavage is seen at K157 (V158-K729, observed 64,108 Da; calc. 64,145 Da), close to the boundary

Supplemental Tables ST6, ST7. Polypeptide assignments for *D. melanogaster* tRNase Z

– LysC and GluC MALDI-ToF Peaks based on spectra in Figure SF6

with the ascending stalk of the FA. Results with *H. sapiens* tRNase ZL (Figures 8, 9) also suggest the presence of a flexible site close to the boundary between the body and the ascending stalk of the FA. Third, while trypsin cleaves heavily at R384K385, LysC samples lysines elsewhere in tether does not at K385.

Proteolysis by GluC (Supplemental Figure SF5B, Supplemental Table ST7) also yields products that support the domain model for tRNase Z flexibility generated by the trypsin data. Initial GluC products that appear around 80 kDa arise from cleavage at E722 and E724 in the C-terminal tail, a little further from the C-terminus than for trypsin. An early cleavage seen at E18 (*e.g.* R19-T743, observed 81,049 Da; calc. 81,049 Da) corresponds approximately to the Nflex basic patch recognized by trypsin and LysC. There are no glutamates from residue 20-66; E18 is thus the only glutamate not adjacent to proline close to the trypsin R25Nflex patch. Fragments cleaved at both E18 and in the C-terminal tail are also present (Supplemental Table ST7).

There is only one glutamate in the FA region; a strong, more homogeneous FA peak is observed in the GluC spectra than with trypsin and LysC, corresponding to Q195- E722 (observed, 59,333 Da; calc, 59, 296 Da).

A low-intensity peak corresponding to the Nflex-Ndom1 fragment R19 - E412 is consistently observed, starting at 5 minutes (observed 43,375 Da, calc. 43,377 Da). Additionally, numerous peaks are found in a region around 34 – 37 kDa, starting at 5 minutes. Although difficult to interpret due to spectra crowding and the presence of doubly protonated ion peaks, most of these peaks indicate cleavages around residue 392–421 toward the carboxy end of the tether, with variable cleavage in the C-terminal tail, corresponding to Cdom. The 30 minute time point is less crowded and yields two peaks, suggesting proteolytically stable Cdom2 fragments of L413-E731 (the major peak - observed, 36,228 Da; calc 36,208 Da) and Y421-E731 (an intermediate amplitude peak - observed, 35,249 Da; calc 35,282 Da). Data generated with LysC and GluC therefore support a model of flexibility in the Nflex, C-terminal tail, FA hand, and tether regions (Figure 11).

**Stable domains and flexible regions in *H. sapiens* tRNase ZL**. The methods used to analyze *D. melanogaster* tRNase Z (described in detail above: Supplemental Tables ST1, ST2, Supplemental Figure SF3, Supplemental Table ST3, Supplemental Appendix 1) were applied to*H. sapiens* tRNase ZL. Polypeptides assigned to the peaks in 1D MALDI-TOF spectra of *H. sapiens* tRNase ZL digested with trypsin are presented in Supplemental Table ST8.

Virtually all of the polypeptides can be assigned to cleavages that fit the model for stable domains and flexible regions (Figure 10; highlighted in yellow in Supplemental Table ST8). Coverage obtained from full length *H. sapiens* tRNase ZL extracted from 2D gels is presented with yellow highlighting in Supplemental Figure SF7. Peptides detected from polypeptides extracted from the 2D gel spots exhaustively digested with trypsin are shown in Supplemental Table ST9 and the peptide analysis by MS-MS is collated in Supplemental Appendix 2.

Supplemental Figure SF9 **Coverage of *H. sapiens* tRNase ZL**

Supplemental Table ST9 ***H. sapiens* tRNase ZL Polypeptides**

REFERENCES

1. Li de la Sierra-Gallay, I., Mathy, N., Pellegrini, O., and Condon, C. 2006. Structure of the ubiquitous 3’ processing enzyme RNase Z bound to transfer RNA. *Nat Struct Mol Biol*. **13**:376-377.
2. Pellegrini O, Li de la Sierra-Gallay I, Piton J, Gilet L, Condon C. 2012. Activation of tRNA maturation by downstream uracil residues in B. subtilis. *Structure*. **20**:1769-77.
3. Li de la Sierra-Gallay, I., Pellegrini, O., and Condon, C. 2005. Structural basis for substrate binding, cleavage and allostery in the tRNA maturase RNase Z. *Nature* **433**, 657-661.
4. Schilling, O., Späth, B., Kostelecky, B., Marchfelder A., Meyer-Klaucke, W. Et al. 2005. Exosite modules guide substrate recognition in the ZiPD/ElaC protein family. *J Biol Chem*. **280**, 17857-17862.
5. Zareen, N., Hopkinson, A., Levinger, L. 2006. Residues in two homology blocks on the amino side of the tRNase Z His domain contribute unexpectedly to pre-tRNA 3’ end processing. *RNA* **12**: 1104-1115.
6. Karkashon, S., Hopkinson, A., and Levinger, L. 2007. tRNase Z Catalysis and Conserved Residues on the Carboxy Side of the His Cluster. *Biochemistry* **46**:9380-9387.
7. Levinger, L., Hopkinson, A., Desetty, R., and Wilson, C. 2009. Effect of changes in the flexible arm on tRNase Z processing kinetics. *J. Biol. Chem.* **284**:15685-15691.
8. Minagawa, A., Takaku, H., Takagi, M., and Nashimoto, M. 2004. A novel endonucleolytic mechanism to generate the CCA 3' termini of tRNA molecules in Thermotoga maritima. *J Biol Chem*. **279**, 15688-15697.
9. Wang Z, Zheng J, Zhang X, Peng J, Liu J, et al. 2012. Identification and sequence analysis of metazoan tRNA 3'-end processing enzymes tRNase Zs. **PLoS One**.;7(9):e44264. doi: 10.1371/journal.pone.0044264. Epub 2012 Sep 4.
10. Tavtigian SV, Simard J, Teng DHF, Abtin V, Baumgard M, et al (2001) A candidate prostate cancer susceptibility gene at chromosome 17p. Nature Genet 27:172-180.
11. Redko Y, Li de la Sierra-Gallay I, Condon C. 2007. When all's zed and done: the structure and function of RNase Z in prokaryotes. *Nat Rev Microbiol*. **5**:278-286.
12. Aravind, L. 1999. An evolutionary classification of the metallo-beta-lactamase fold proteins. *In Silico Biol.* **1**: 69-91.
13. Dominski, Z. 2007. Nucleases of the metallo-β-lactamase family and their role in DNA and RNA metabolism. *Crit. Rev. Biol. & Mol. Biol* **42**:67-93.
14. Kostelecky, B., Pohl, E., Vogel, A., Schilling, O., and Meyer-Klaucke, W. 2006. The crystal structure of the zinc phosphodiesterase from *Escherichia coli* provides insight into function and cooperativity of tRNase Z family proteins. *J. Bacteriol*. **188**, 1607-1614.
15. Ishii R, Minagawa A, Takaku H, Takagi M, Nashimoto M, et al. 2007. The structure of the flexible arm of Thermotoga maritima tRNase Z differs from those of homologous enzymes. *Acta Crystallogr*. **63**: 637-641.
16. DeLano, W.L. 2002. The PyMOL Molecular Graphics System on World Wide Web [**http://www.pymol.org**](http://www.pymol.org)
17. Yan, H., Zareen, N., and Levinger, L. 2006. Naturally occurring mutations in human mitochondrial pre-tRNASer(UCN) can affect the tRNase Z cleavage site, processing kinetics and substrate secondary structure. *J. Biol. Chem*. **281**: 3926-3935.
18. Hopkinson, A., and Levinger, L. 2008. Effects of conserved D/T loop substitutions in the pre-tRNA substrate on tRNase Z catalysis. *RNA Biol*. **5**:104-111.
